# Supplementary material for: METTL3/MYCN cooperation drives neural crest differentiation and provides therapeutic vulnerability in neuroblastoma
Source: EMBO J. 2024 Nov 11;43(24):6310–35. doi: 10.1038/s44318-024-00299-8 (PMC11649786; doi:10.1038/s44318-024-00299-8)
Supplement: Supplementary file 1 — Appendix [file 44318_2024_299_MOESM1_ESM.pdf]

# Appendix

## METTL3/MYCN cooperation drives neural crest differentiation and provides therapeutic vulnerability in neuroblastoma

Thombare, Vaid, et al

*Corresponding author: Tanmoy Mondal, tanmoy.mondal@gu.se

## Table of Contents

Appendix Figure S1. ........................................ 1

Appendix Figure S2. ........................................ 6

Appendix Figure S3. ........................................ 9

Appendix Figure S4. ........................................ 11

Appendix Figure S5. ........................................ 14

Appendix Figure S6. ........................................ 18

Appendix Supplementary Methods ........................................ 22

References ........................................ 32

## Appendix Figure S1

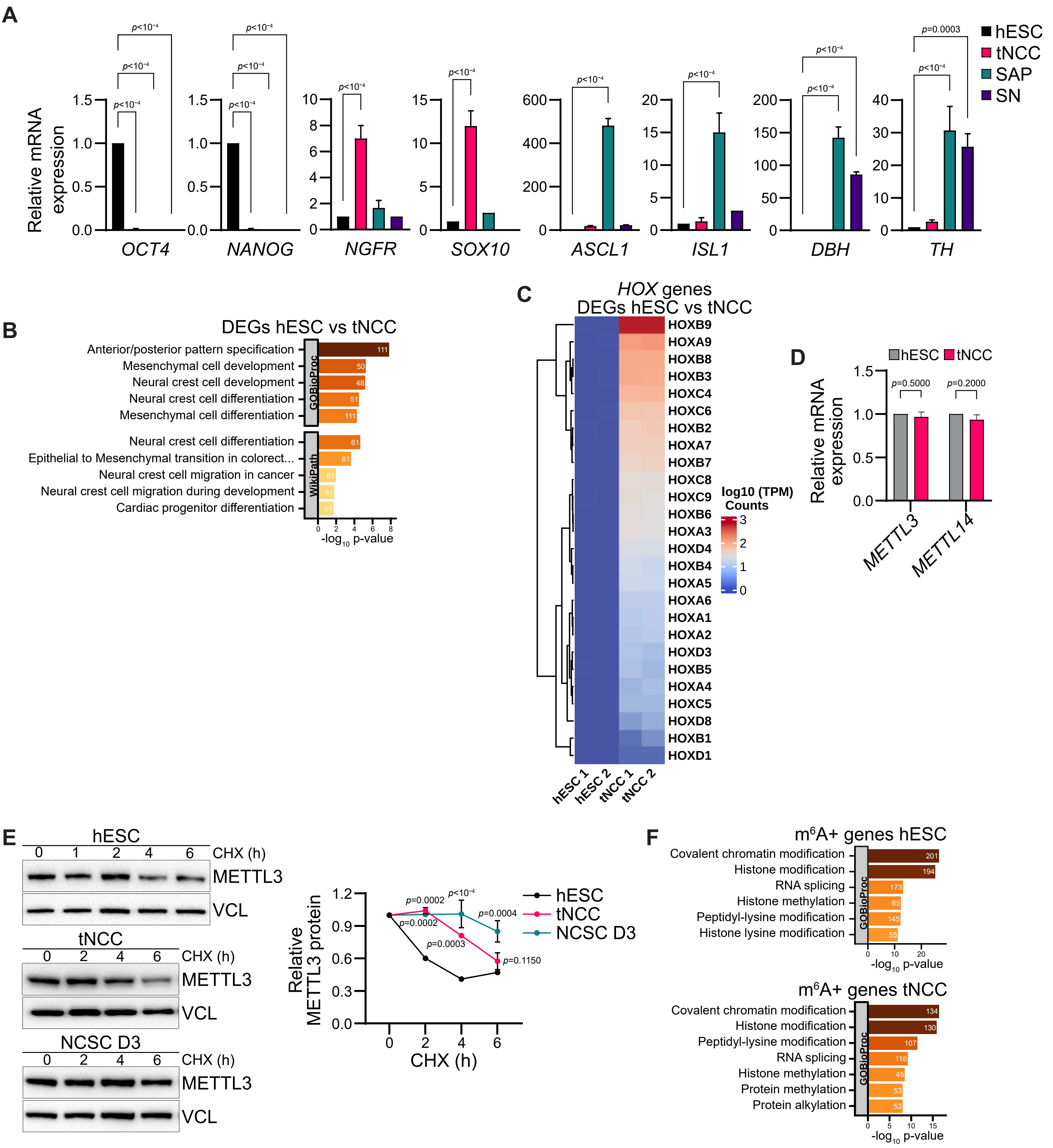

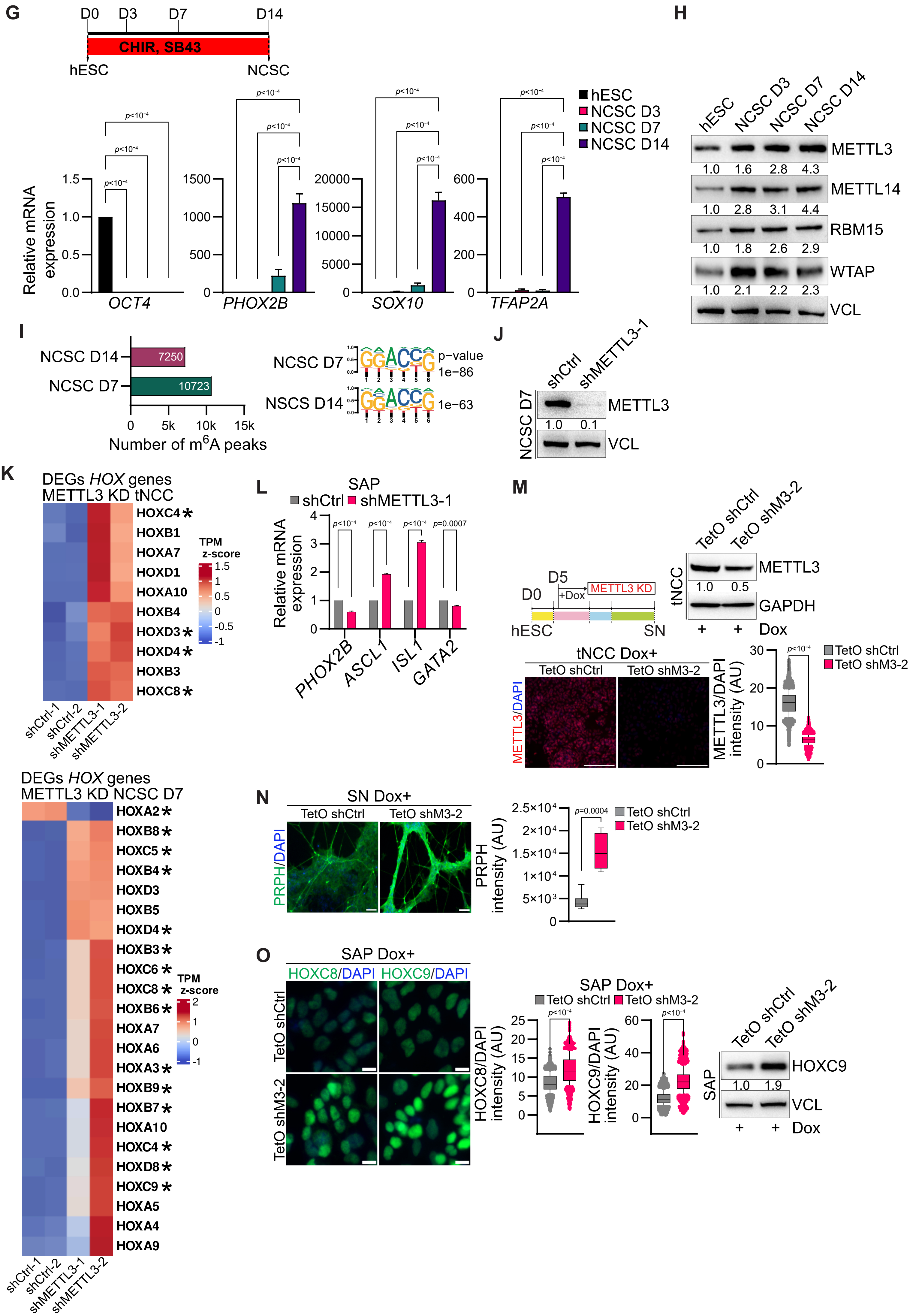

G
D0 D3 D7 D14
CHIR, SB43
hESC NCSC
Relative mRNA expression
OCT4 PHOX2B SOX10 TFAP2A
hESC
NCSC D3
NCSC D7
NCSC D14
H
hESC NCSC D3 NCSC D7 NCSC D14
METTL3 1.0 1.6 2.8 4.3
METTL14 1.0 2.8 3.1 4.4
RBM15 1.0 1.8 2.6 2.9
WTAP 1.0 2.1 2.2 2.3
VCL
I
NCSC D14 7250
NCSC D7 10723
Number of m6A peaks
NCSC D7 p-value 1e−86
NSCS D14 1e−63
J
shCtrl shMETTL3-1
NCSC D7
METTL3 1.0 0.1
VCL
K
DEGs HOX genes METTL3 KD tNCC
HOXC4* HOXB1 HOXA7 HOXD1 HOXA10 HOXB4 HOXD3* HOXD4* HOXB3 HOXC8*
TPM z-score
shCtrl-1 shCtrl-2 shMETTL3-1 shMETTL3-2
DEGs HOX genes METTL3 KD NCSC D7
HOXA2* HOXB8* HOXC5* HOXB4* HOXD3 HOXB5 HOXD4* HOXB3* HOXC6* HOXC8* HOXB6* HOXA7 HOXA6 HOXA3* HOXB9* HOXB7* HOXA10 HOXC4* HOXD8* HOXC9* HOXA5 HOXA4 HOXA9
L
SAP
shCtrl shMETTL3-1
Relative mRNA expression
p=0.0007
PHOX2B ASCL1 ISL1 GATA2
M
D0 D5 +Dox METTL3 KD
hESC SN
TetO shCtrl TetO shM3-2
tNCC
METTL3 1.0 0.5
GAPDH
+ + Dox
tNCC Dox+
TetO shCtrl TetO shM3-2
METTL3/DAPI
METTL3/DAPI intensity (AU)
N
SN Dox+
TetO shCtrl TetO shM3-2
PRPH/DAPI
PRPH intensity (AU)
p=0.0004
O
SAP Dox+
HOXC8/DAPI HOXC9/DAPI
TetO shCtrl
TetO shM3-2
HOXC8/DAPI intensity (AU)
HOXC9/DAPI intensity (AU)
SAP
HOXC9 1.0 1.9
VCL
+ + Dox

**Appendix Figure S1. Lineage marker expression and METTL3 functionality in tNCC differentiation.**

(**A**) Bar plots depict the relative mRNA expression levels of lineage markers at different stages, including human embryonic stem cells (hESC), trunk neural crest cells (tNCC), followed by their further differentiation into sympathoadrenal progenitors (SAP), and, ultimately, into sympathetic neurons (SN). Pluripotency markers *NANOG* and *OCT4*, tNCC markers *NGFR* and SOX10, SAP markers *ASCL1* and *ISL1*, and SAP/SN markers *DBH* and TH were quantified. *GAPDH* served as a reference for qPCR data normalization. The presented data represents the mean±SD from three independent biological replicates. Statistical analysis was conducted using one-way ANOVA with Dunnett's multiple comparisons test. (**B**) Top enriched terms associated with DEGs (hESC vs. tNCC) were identified using enrichGO, with *p*-values obtained through Fisher's exact test. (**C**) Heatmap demonstrating the top differentially expressed HOX genes between hESC and tNCC. (**D**) Relative mRNA expression of *METTL3* and *METTL1*4 in hESC and tNCC as determined by RT-qPCR. *GAPDH* served as the normalization reference for qPCR data. The results are presented as mean±SD from three independent biological replicates. Statistical analysis was conducted using two-way ANOVA with Šídák's multiple comparisons test. (**E**) Immunoblot displays the levels of METTL3 in hESC, tNCC, and neural crest stem cells (NCSC) at day 3 following cycloheximide (CHX) chase for specified time intervals. Vinculin was utilized as the loading control. Line plots presenting the quantification of METTL3 levels after CHX chase at the indicated time points. The experiments were replicated three times, and the data are presented as mean±SD in the graph. Statistical analysis was performed using two-way ANOVA with Dunnett's multiple comparisons test. (**F**) Top enriched terms associated with the $m^6A$ positive ($m^6A$ +) genes in hESC (top) and tNCC (bottom) were identified using enrichGO, with *p*-values obtained through Fisher's exact test. (**G**) The schematic diagram delineates the critical stages in the differentiation of hESC into neural crest stem cells (NCSC). In the bottom panel, the relative mRNA expression levels of lineage markers at the hESC and NCSC stages are presented. *OCT4* serves as a pluripotency marker, while *PHOX2B*, *SOX10*, and *TFAP2A* are employed as markers for NCSC. *GAPDH* is utilized for normalizing the qPCR data. The data is represented as mean±SD of three independent biological replicates, and statistical analysis was conducted using two-way ANOVA with Dunnet's multiple comparisons test. (**H**) Representative immunoblot shows the expression levels of METTL3, METTL14, RBM15, and WTAP in hESC and at various stages during NCSC differentiation. Vinculin serves as the loading control, and the values below the blots indicate the fold change in the levels of METTL3 and METTL14. The experiments were repeated three times. (**I**) The

left panel displays the total number of $m^6A$ peaks in day 7 NCSC progenitors and day 14 NCSC, while the right panel presents the motifs identified through *de novo* motif analysis in the $m^6A$ peaks, and *p*-values were obtained using the HOMER tool. (**J**) Immunoblot shows METTL3 protein levels after shRNA-mediated METTL3 KD in hESC. Vinculin was used as a loading control. The values below indicate the fold change in levels of METTL3. The experiments were repeated three times. (**K**) Heatmap summarizes the top differentially expressed *HOX* genes between control and METTL3 KD tNCC and in NCSC. The '*' indicates the presence of $m^6A$ peaks in the gene. (**L**) Relative mRNA expression of *PHOX2B*, *ASCL1*, *ISL1*, and *GATA2* in SAP following METTL3 KD (shMETTL3-1) as determined by RT-qPCR. *GAPDH* served as the normalization reference for qPCR data. The results are presented as mean±SEM from three independent biological replicates. Statistical analysis was conducted using two-way ANOVA with Šídák's multiple comparisons test. (**M**) In the top panel, a schematic diagram and a representative immunoblot of METTL3 KD in Dox dependent manner during tNCC differentiation. The bottom panel displays representative immunofluorescence (IF) images showing METTL3 (red) expression in METTL3 KD tNCC following Dox induction. Quantification of METTL3 signal intensity normalized to DAPI after METTL3 KD is presented in the box-whisker plots. The median is indicated by a horizontal line, the boxes represent the $25^{th}$ to $75^{th}$ percentiles, the whiskers show the $10^{th}$ to $90^{th}$ percentiles, and any outliers beyond this range are displayed as individual dots. This analysis was conducted across three independent biological replicates, with signal intensity measurements taken from over 3000 cells. Statistical significance was determined using a two-tailed unpaired *t*-test. Scale bar represents 100 µm. (**N**) Dox induced TetO shCtrl and TetO shM3-2 tNCC were differentiated to SN and IF was performed with PRPH (green) antibody. Box-whisker plots show PRPH signal intensity. The median is indicated by a horizontal line, the boxes represent the $25^{th}$ to $75^{th}$ percentiles, the whiskers show the $10^{th}$ to $90^{th}$ percentiles, and any outliers beyond this range are displayed as individual dots. Experiments were performed in three independent biological replicates. Two-tailed unpaired *t*-test was used. Scale bar represents 100 µm. (**O**) Left: TetO shCtrl and TetO shM3-2 tNCC were differentiated to SAP and IF was performed with HOXC8 and HOXC9 antibodies. Scale bar represents 10 µm. Middle: Box-whisker plots illustrate the quantification of the signal intensity for HOXC8 and HOXC9 normalized to DAPI intensity. The median is indicated by a horizontal line, the boxes represent the $25^{th}$ to $75^{th}$ percentiles, the whiskers show the $10^{th}$ to $90^{th}$ percentiles and any outliers beyond this range are displayed as individual dots. Data are from three independent experiments, with signal intensity measurements taken from over 1800 cells. Statistical analysis was performed using a two-tailed unpaired *t*-test. Right: Representative immunoblot

shows the levels of HOXC9, in SAP with either TetO shCtrl or TetO shM3-2. Vinculin was loading control. The values below the blots indicate the fold change (normalized to loading control) in the levels of HOXC9. The experiments were repeated three times.

## Appendix Figure S2

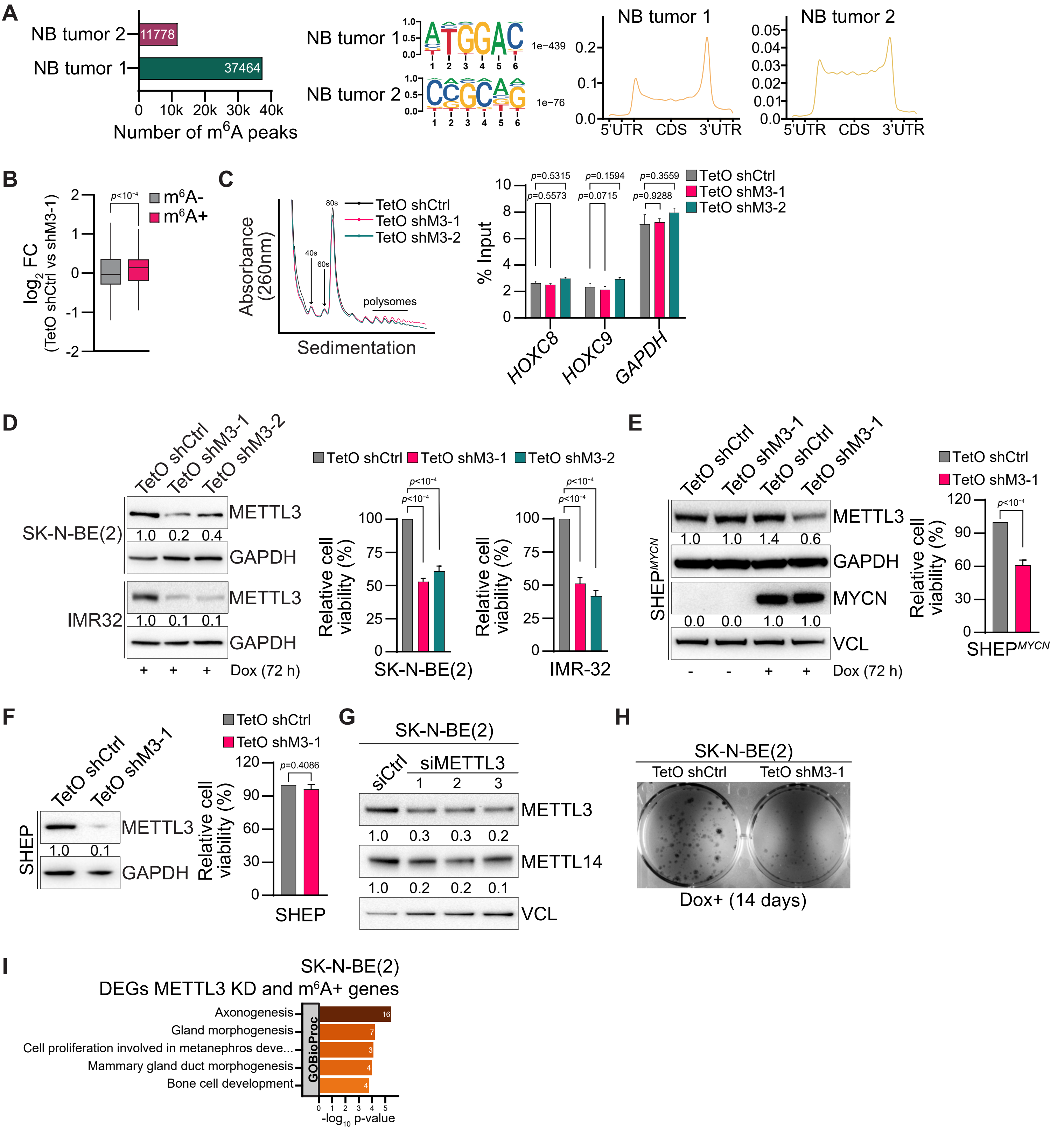

**Appendix Figure S2. m$^6$A profiling and effects of METTL3 KD in MNA NB cells.**

(**A**) Left: The total number of m$^6$A peaks identified, and (middle) identified motifs from *de novo* motif analysis of m$^6$A peaks enriched in MNA NB tumor samples. *P*-values were obtained using the HOMER tool. Right: Metagene analysis showing relative m$^6$A peak density at genes in both MNA NB tumor samples. (**B**) Box plots show fold change in RNA expression after METTL3 KD (TetO shCntrl vs. TetO shM3-1) in SK-N-BE(2) cells. Genes are categorized based on the presence of m$^6$A into m$^6$A+ (n=2687)or genes lacking m$^6$A (m$^6$A-, n=6222) where 'n' is number of genes. Statistical analysis was performed using the Wilcoxon matched-pairs signed rank test. (**C**) Left: Polysome profile in Control and METTL3 KD SK-N-BE(2) cells. Right: Bar plots show enrichment (% Input) of *HOXC8*, *HOXC9*, and *GAPDH* transcripts present in the polysome fraction of 72 h Dox induced control and METTL3 KD SK-N-BE(2) cells. Data are presented as mean±SEM from two independent experiments each with three technical replicates. Statistical analysis was performed using two-way ANOVA with Dunnett's multiple comparisons test. (**D**) Left: Immunoblot showing METTL3 expression in Dox induced (72 h) control and METTL3 KD SK-N-BE(2) and IMR-32 cells. GAPDH was used as a loading control. The values below indicate the fold change in levels of METTL3. The experiments were repeated three times. Right: Bar plots show cell viability of SK-N-BE(2) and IMR-32 cells with METTL3 KD (6 days post Dox induction). Data are presented as mean±SEM from three independent experiments. Statistical significance was determined using a one-way ANOVA with Dunnett's multiple comparisons test. (**E**) Left: Representative immunoblot showing simultaneous MYCN overexpression and METTL3 KD *via* Dox induction in SHEP (SHEP$^{MYCN}$) cells. GAPDH and vinculin were loading controls. The values below indicate the fold change in levels of METTL3 and MYCN. The experiments were repeated three times. Right: Bar plots show the cell viability of SHEP$^{MYCN}$ cells with METTL3 KD (6 days post Dox induction). Data are presented as mean±SEM from three independent experiments. Two-tailed unpaired *t*-test was used. (**F**) Left: Immunoblot showing METTL3 expression in Dox induced control and METTL3 KD SHEP cells. The experiments were repeated three times. Right: Bar plots show the cell viability of SHEP cells with METTL3 KD (6 days post Dox induction). Data are presented as mean±SEM from three independent experiments. Two-tailed unpaired *t*-test was used. (**G**) Representative immunoblot showing METTL3 and METTL14 expression following siRNA-mediated METTL3 KD. Vinculin was used as a loading control. The values below indicate the fold change in levels of METTL3 and METTL14. The experiments were repeated three times. (**H**) Representative images from colony formation assay performed in SK-N-BE(2) cells with METTL3 KD (14 days post Dox induction). The experiments were repeated three times. (**I**) Top

enriched terms associated with DEGs (Ctrl Vs. METTL3 KD) having $m^6A$ peaks ($m^6A$+) in SK-N-BE(2) cells were identified using enrichGO, with *p*-values obtained through Fisher's exact test.

**Appendix Figure S3**

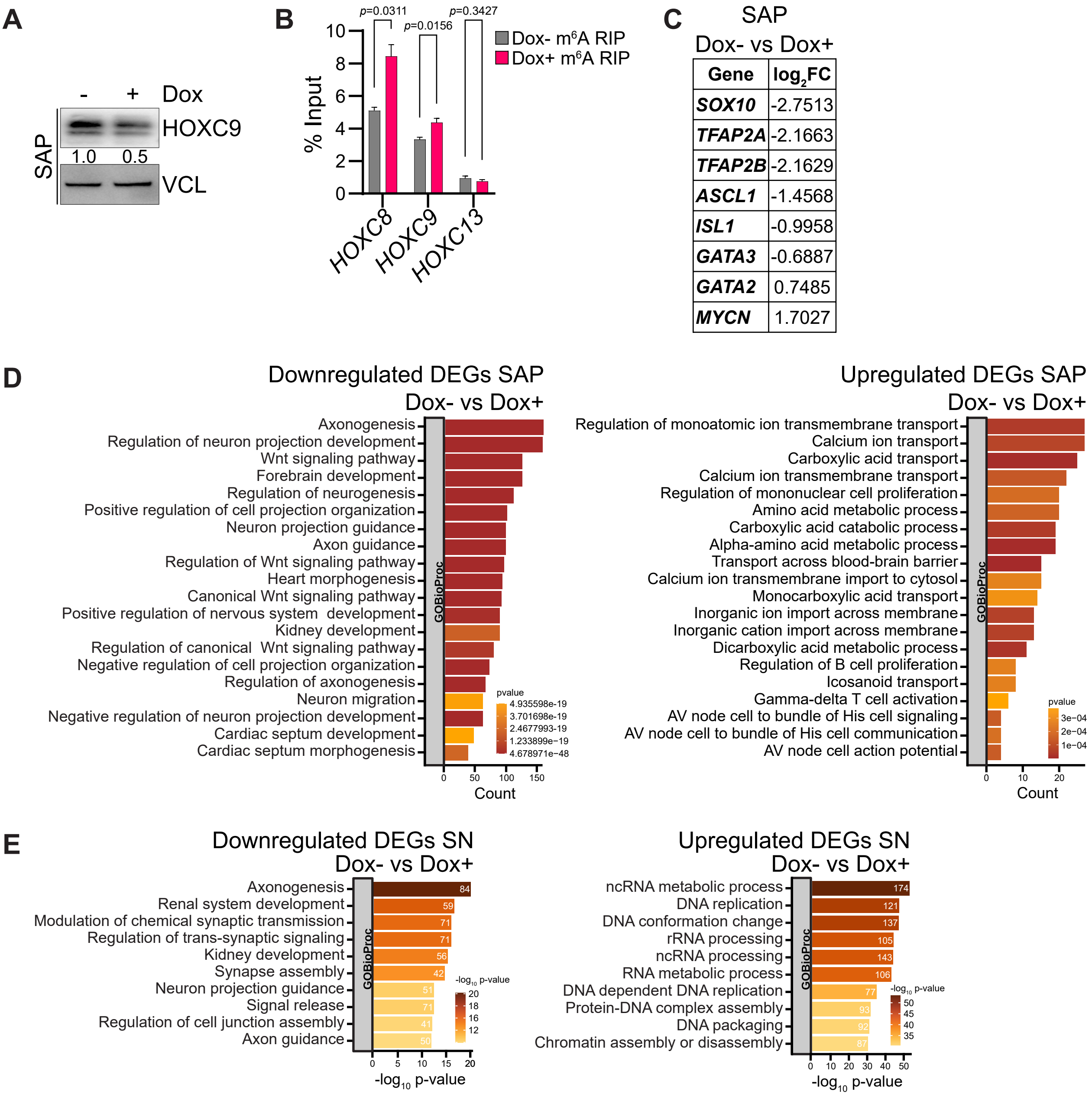

**Appendix Figure S3. Gene expression in MYCN overexpressing SAP cells.**

(**A**) Representative immunoblot showing expression of HOXC9 in SAP following Flag-MYCN overexpression (Dox+, from day 5 onwards) and in control (Dox-). Vinculin was used as a loading control. The values below indicate the fold change in levels of HOXC9. The experiments were repeated three times. (**B**) $m^6A$ RIP-qPCR data showing enrichment of both *HOXC8* and *HOXC9* in MYCN overexpressing (Dox+, from day 5 onwards) and control (Dox-) SAP cells. Data are represented as a percentage of input. *HOXC13* serves as a negative control. Data are presented as mean±SEM from three independent experiments and statistical analysis was conducted using two-way ANOVA with Šídák's multiple comparisons test. (**C**) Differentially expressed genes (DEGs) between control (Dox-) and Flag-MYCN overexpression (Dox +, from day 5 onwards) SAP cells. Genes related to critical role in SAP differentiation and MYCN are presented. The expression values ($log_2$ FC) were determined from RNA-seq data. (**D-E**) Top enriched terms associated with downregulated (left) and upregulated (right) genes in Control (Dox-) vs. Flag-MYCN overexpressed (Dox +, from day 5 onwards) in SAP (D) and SN stage cells (E). Enriched terms were identified using enrichGO, with *p*-values obtained through Fisher's exact test.

# Appendix Figure S4

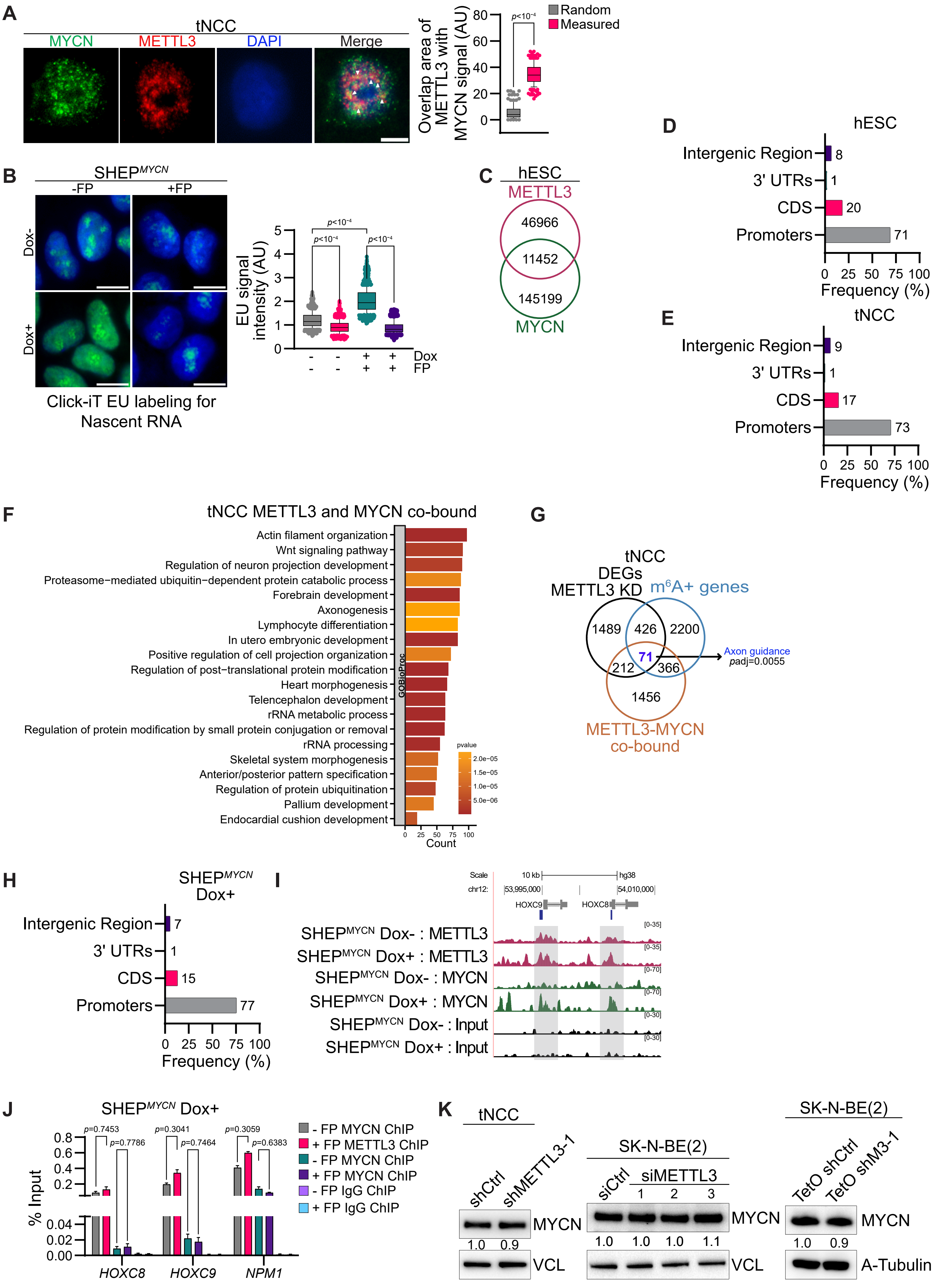

**Appendix Figure S4. MYCN and METTL3 interactions and chromatin binding dynamics.**

(**A**) Left: MYCN (green) and METTL3 (red) IF was performed in tNCC. White arrows indicate the colocalization of METTL3 and MYCN. Right: Box-whisker plots show the overlap area of METTL3 with MYCN. The area of the overlapping METTL3 and MYCN signal was quantified using the Interaction Factor package in ImageJ. The median is indicated by a horizontal line, the boxes represent the 25$^{th}$ to 75$^{th}$ percentiles, the whiskers show the 10$^{th}$ to 90$^{th}$ percentiles and any outliers beyond this range are displayed as individual dots. To assess the significance of the observed overlapped area, the METTL3 signal was randomized for each image. The means of 20 randomizations were then plotted alongside the experimentally observed values. This analysis allowed us to evaluate the statistical significance of the observed overlap between METTL3 and MYCN signals. At least 50 cells were counted from two independent biological replicates. Two-tailed unpaired *t*-test was used. Scale bar represents 5 µm. (**B**) Nascent RNA detection with 5-Ethynyl Uridine (EU) labeling followed by performing "click chemistry" in SHEP$^{MYCN}$ cells treated with either DMSO or Flavopiriol (FP). Box-whisker plots show EU signal intensity. The median is indicated by a horizontal line, the boxes represent the 25$^{th}$ to 75$^{th}$ percentiles, the whiskers show the 10$^{th}$ to 90$^{th}$ percentiles, and any outliers beyond this range are displayed as individual dots. Data are from three independent biological replicates. Statistical significance was determined using a one-way ANOVA with Tukey's multiple comparisons test. Scale bar represents 10 µm. (**C**) Venn diagram comparison of METTL3 and MYCN binding sites determined from ChIP-seq experiments performed in hESC. (**D-E**) Distribution of METTL3 and MYCN co-bound regions by genomic features for hESC (D) and tNCC (E). METTL3 and MYCN co-bound regions were determined using the ChIP-seq experiments. (**F**) Top enriched terms associated with METTL3 and MYCN co-bound genes in tNCC were identified using enrichGO, with *p*-values obtained through Fisher's exact test. (**G**) Three-way Venn diagram comparing DEGs (shCtrl vs. shMETTL3-1), m$^6$A positive genes, and METTL3-MYCN co-bound regions in tNCC. *P*-value was obtained through Fisher's exact test. (**H**) Distribution of METTL3 and MYCN co-bound regions by genomic features for SHEP$^{MYCN}$ cells after Dox induction. METTL3 and MYCN co-bound regions were determined using the ChIP-seq experiments. (**I**) Genome Browser screenshot showing METTL3, MYCN ChIP-seq signals over the *HOXC8* and *HOXC9* gene locus in SHEP$^{MYCN}$ cells before and after Dox induction. METTL3 and MYCN overlapping peak coordinates in SHEP$^{MYCN}$ after Dox induction are indicated by blue bars. (**J**) MYCN, METTL3, IgG ChIP-qPCR data, represented as percentage input over selected genes in Dox induced SHEP$^{MYCN}$ cells treated with or without FP. Data are shown as mean±SEM from three independent biological replicates. Statistical analysis was conducted using

two-way ANOVA with Šídák's multiple comparisons test. (**K**) Immunoblot showing MYCN expression following METTL3 KD in tNCC, SK-N-BE(2) cells. The values below indicate the fold change in levels of MYCN. The experiments were repeated three times. METTL3 KD blots are presented in **Fig. 1I**, and Appendix Fig. S2G, E respectively.

# Appendix Figure S5

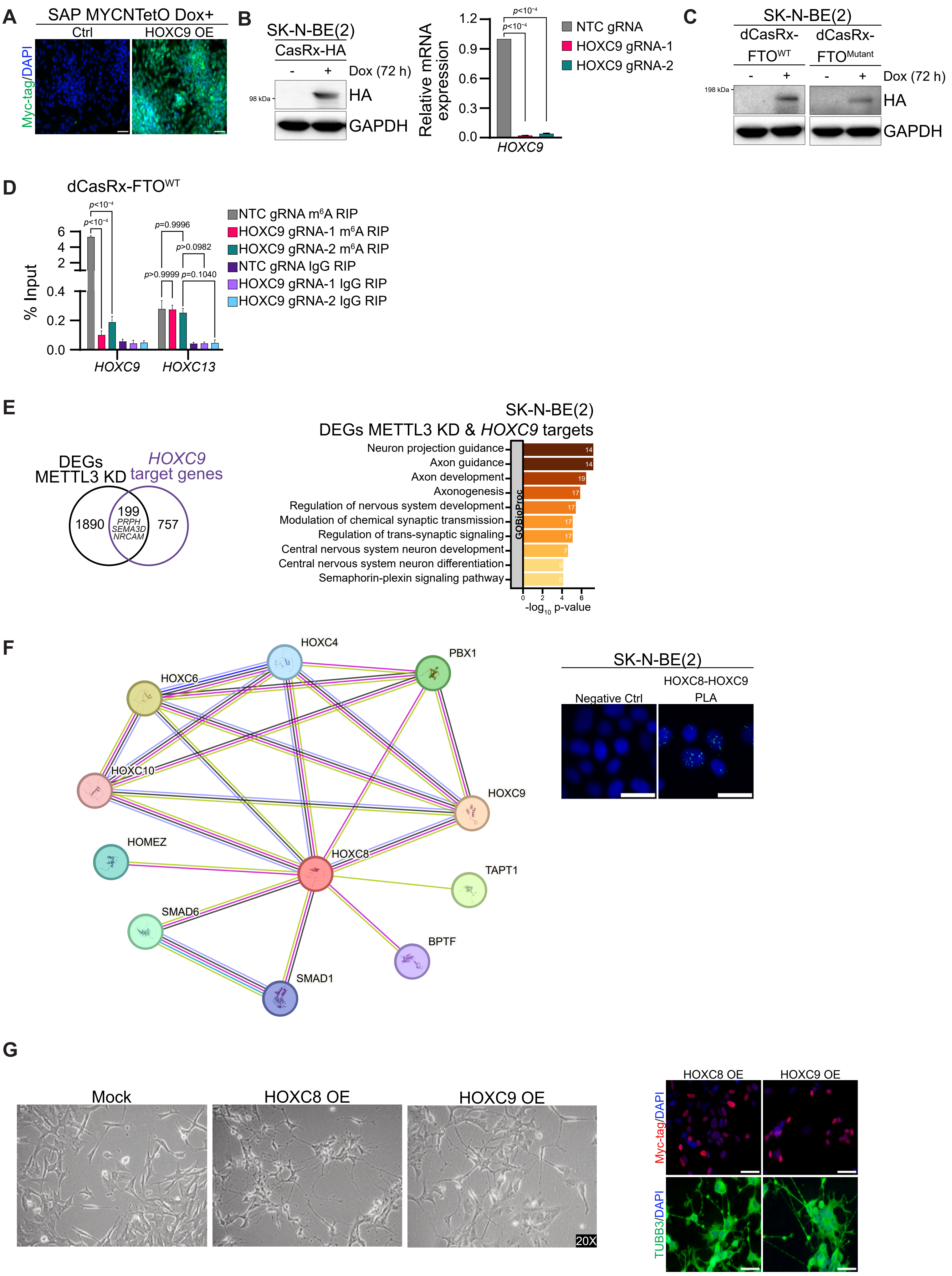

**H**

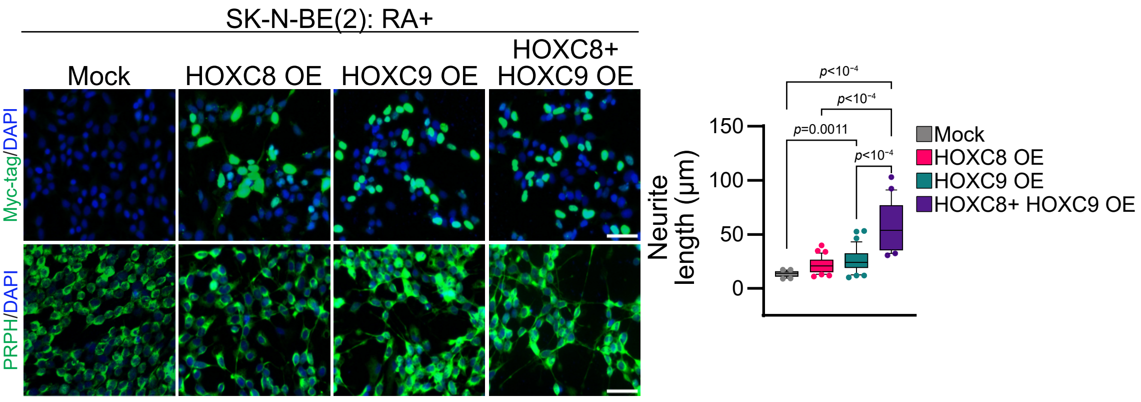

SK-N-BE(2): RA+
Mock
HOXC8 OE
HOXC9 OE
HOXC8+
HOXC9 OE
Myc-tag/DAPI
PRPH/DAPI
Neurite length (µm)
150
100
50
0
p<10⁻⁴
p<10⁻⁴
p=0.0011
p<10⁻⁴
Mock
HOXC8 OE
HOXC9 OE
HOXC8+ HOXC9 OE


**I**

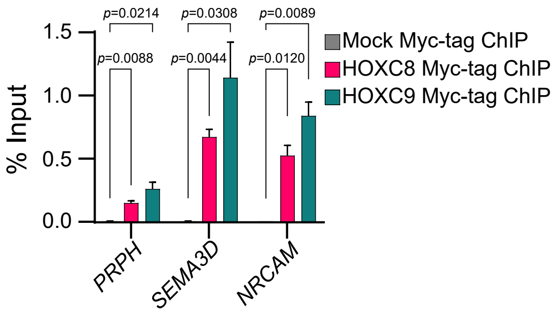

% Input
1.5
1.0
0.5
0.0
p=0.0214
p=0.0308
p=0.0089
p=0.0088
p=0.0044
p=0.0120
Mock Myc-tag ChIP
HOXC8 Myc-tag ChIP
HOXC9 Myc-tag ChIP
PRPH
SEMA3D
NRCAM

**Appendix Figure S5. Functional analysis of HOXC9 in SK-N-BE(2) cells.**

(**A**) IF showing expression of HOXC9 detected by Myc-tag (green) in Flag-MYCN (Dox+, from day 5 onwards) SAP with either control (Ctrl) or HOXC9 overexpression (OE). HOXC9 overexpression was performed from D9 of differentiation. The experiments were repeated three times. Scale bar represents 100 µm. (**B**) Left: Immunoblot to verify induction of HA-tagged Cas-Rx in SK-N-BE(2) cells. Right: Relative mRNA expression of *HOXC9* in SK-N-BE(2) cells expressing HA-CasRx with either non-template control (NTC gRNA) or HOXC9 guide RNAs (HOXC9 gRNA-1, HOXC9 gRNA-2. Data are presented as mean±SEM from three independent experiments and statistical significance was determined using a one-way ANOVA with Dunnett's multiple comparisons test. (**C**) Immunoblot to verify induction of HA-tagged dCasRx-FTO$^{WT}$ (wild-type) and dCasRx-FTO$^{Mutant}$ (catalytically dead-H231A and D233A mutant) in SK-N-BE(2) cells. GAPDH was used as a loading control. The experiments were repeated three times. (**D**) m$^6$A RIP-qPCR data showing enrichment of HOXC9 and HOXC13 (control) in SK-N-BE(2) cells expressing dCasRx-FTO$^{WT}$ with either NTC gRNA or HOXC9 guide RNAs (HOXC9 gRNA-1, HOXC9 gRNA-2). Data are presented as bar plots showing the mean±SEM from three independent biological replicates, expressed as a percentage of input. IgG served as a negative control. Statistical analysis was performed using two-way ANOVA followed by Šídák's multiple comparisons test. (**E**) Left: Venn diagram comparison of HOXC9 target genes (Wang *et al*, 2013) [genes with HOXC9 ChIP-seq peak and 1.5 fold change in expression between control vs HOXC9 overexpression] and DEGs (Control Vs. METTL3 KD) in SK-N-BE(2) cells. Right: Top enriched terms associated with the overlapping genes from the left panel, identified using enrichGO, with *p*-values obtained through Fisher's exact test. (**F**) Left: Interaction network of HOXC8 obtained using STRING-db with default parameters. Right: Proximity ligation assay (PLA) showing the HOXC8 and HOXC9 PLA signal (green) between in SK-N-BE(2) cell nucleus (marked by DAPI). The negative control shows PLA with only the HOXC8 antibody. The experiments were repeated three times. Scale bar represents 50 µm. (**G**) Left: Brightfield images of SK-N-BE(2) cells with stable overexpression of HOXC8 and HOXC9. Right: Representative IF showing TUBB3 (green) and overexpression of MYC-tagged HOXC8 and HOXC9 (red) in SK-N- BE(2) cells. Scale bar represents 50 µm. (**H**) Left: Representative IF showing staining for PRPH and MYC-tagged HOXC8 and HOXC9 in SK-N-BE(2) cell transiently overexpressing HOXC8 and HOXC9 individually or in combination followed by retinoic acid (RA) mediated differentiation for 3 days. Scale bar represents 50 µm. Right: Box-whisker plots show the quantification of the average neurite branch length. The median is indicated by a horizontal line, the boxes represent the 25$^{th}$ to 75$^{th}$ percentiles, the whiskers show the 10$^{th}$

to 90$^{th}$ percentiles, and any outliers beyond this range are displayed as individual dots. Data are from three independent experiments. Statistical analysis was performed using one-way ANOVA with Tukey's *post hoc* test. (**I**) Myc-tag ChIP qPCR data, represented as percentage input over selected genes in SK-N-BE(2) overexpressing either Myc-tagged HOXC8, HOXC9, or mock. Data are represented as bar plots and shown as mean±SD from three independent biological replicates. Statistical analysis was performed using two-way ANOVA with Dunnett's multiple comparisons test.

# Appendix Figure S6

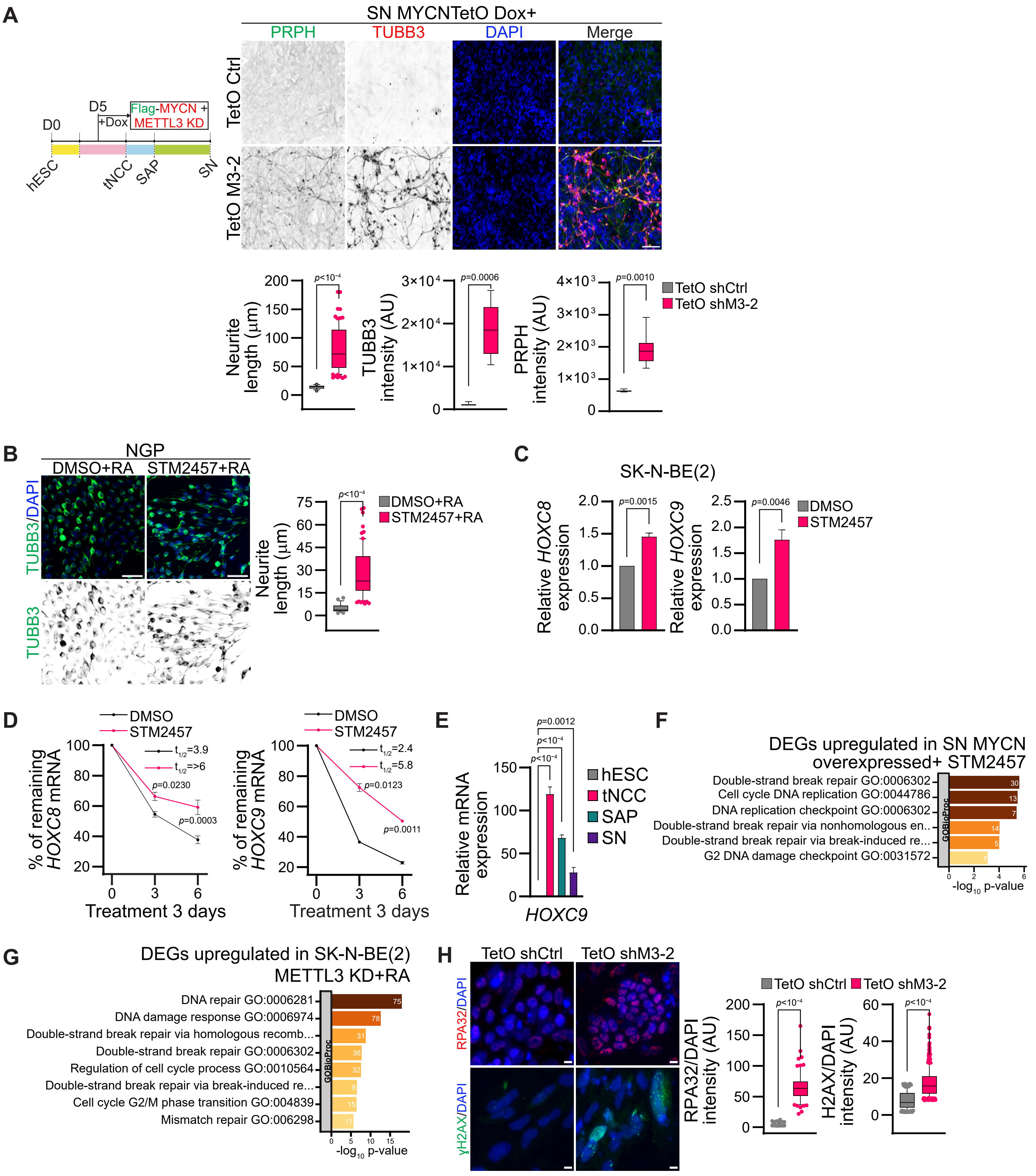

I

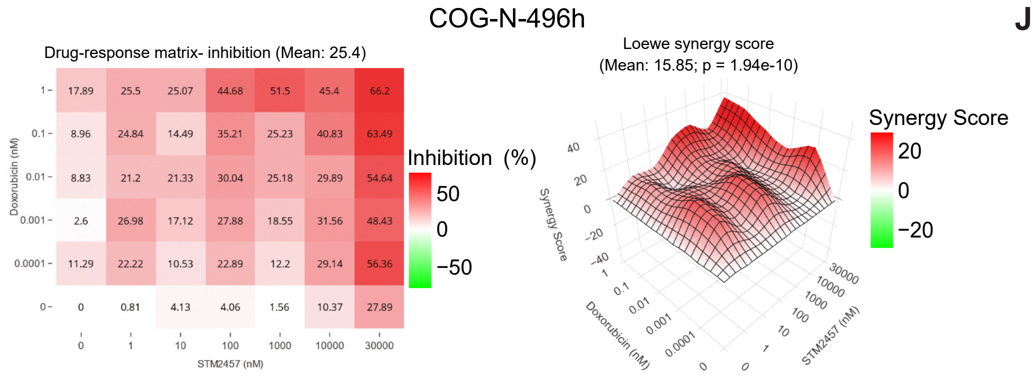

COG-N-496h
Drug-response matrix- inhibition (Mean: 25.4)
Doxorubicin (nM)
STM2457 (nM)
1 17.89 25.5 25.07 44.68 51.5 45.4 66.2
0.1 8.96 24.84 14.49 35.21 25.23 40.83 63.49
0.01 8.83 21.2 21.33 30.04 25.18 29.89 54.64
0.001 2.6 26.98 17.12 27.88 18.55 31.56 48.43
0.0001 11.29 22.22 10.53 22.89 12.2 29.14 56.36
0 0 0.81 4.13 4.06 1.56 10.37 27.89
0 1 10 100 1000 10000 30000
Inhibition (%)
50
0
−50
Loewe synergy score
(Mean: 15.85; p = 1.94e-10)
Synergy Score
Doxorubicin (nM)
STM2457 (nM)
Synergy Score
20
0
−20


J

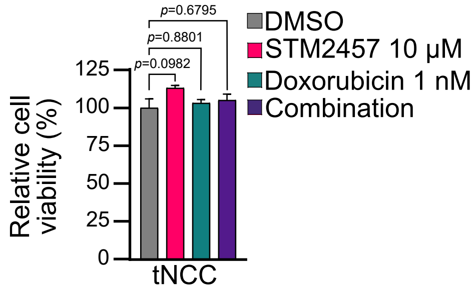

p=0.6795
p=0.8801
p=0.0982
Relative cell viability (%)
125
100
75
50
25
0
tNCC
DMSO
STM2457 10 μM
Doxorubicin 1 nM
Combination


K

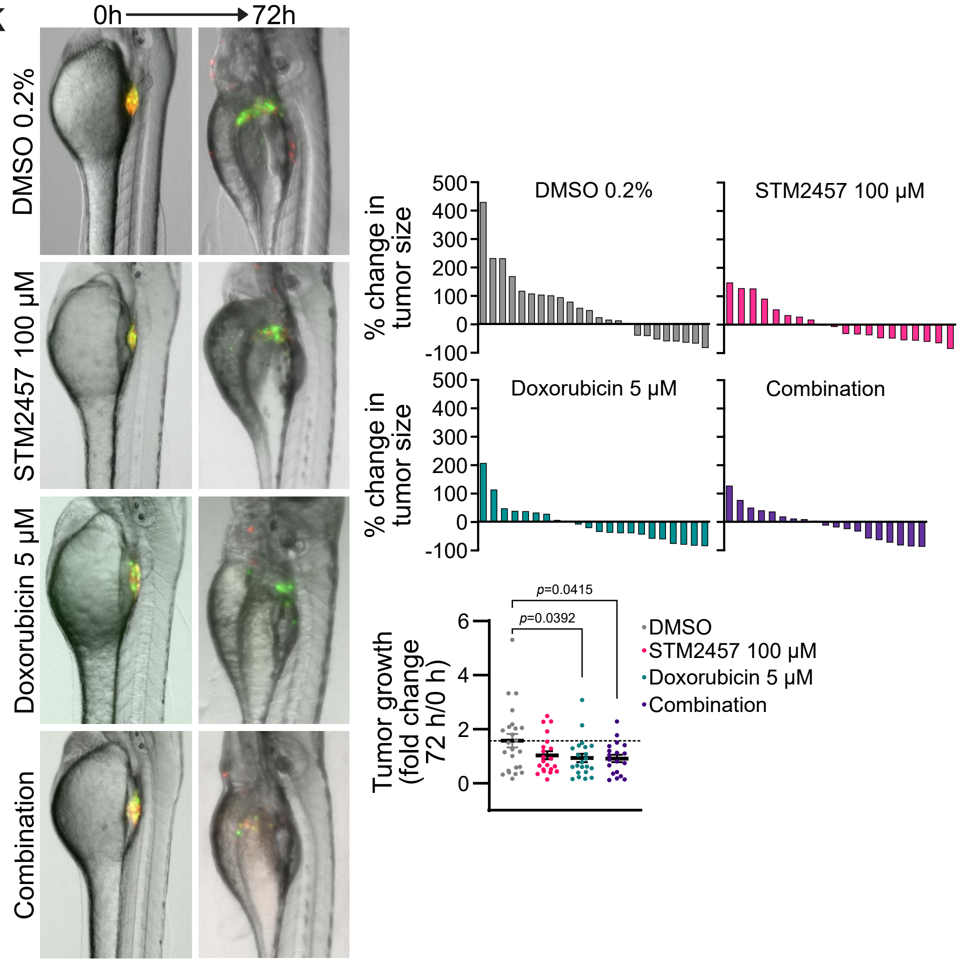

0h
72h
DMSO 0.2%
STM2457 100 μM
Doxorubicin 5 μM
Combination
% change in tumor size
500
400
300
200
100
0
-100
DMSO 0.2%
STM2457 100 μM
Doxorubicin 5 μM
Combination
Tumor growth (fold change 72 h/0 h)
6
4
2
0
p=0.0415
p=0.0392
DMSO
STM2457 100 μM
Doxorubicin 5 μM
Combination


L

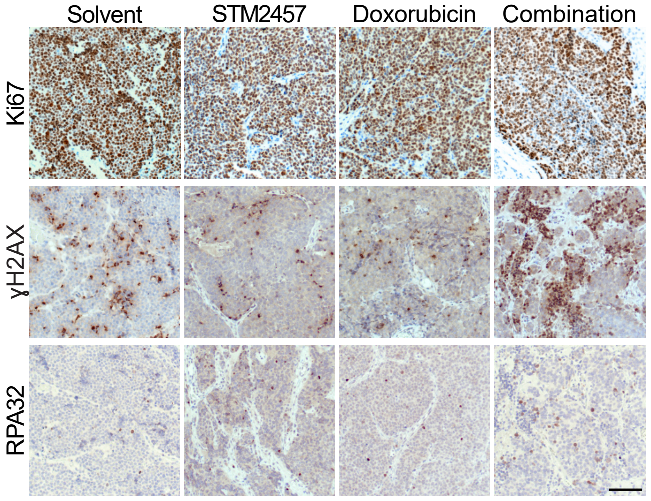

Solvent
STM2457
Doxorubicin
Combination
Ki67
γH2AX
RPA32

**Appendix Figure S6. Functional role of MYCN and METTL3 interaction on differentiation and METTL3 inhibition mediated therapeutic approaches in MNA NB.**

(**A**) Left: Schematic diagram showing a timeline of simultaneous induction of Flag- MYCN and METTL3 KD during differentiation (Dox+, from day 5 onwards). Right: Representative IF showing PRPH (green) and TUBB3 (red) expression in SN stage cells with simultaneous Flag-MYCN overexpression and METTL3 KD induced by Dox at the indicated day. Box-whisker plots showing either PRPH, TUBB3 intensity or quantification of neurite length. The median is indicated by a horizontal line, the boxes represent the 25$^{th}$ to 75$^{th}$ percentiles, the whiskers show the 10$^{th}$ to 90$^{th}$ percentiles, and any outliers beyond this range are displayed as individual dots. Data are from three independent biological replicates. Statistical significance was determined using a two-tailed unpaired *t*-test. Scale bar represents 100 µm. (**B**) Left: Representative IF images of TUBB3 (green) in NGP cells that were pre-treated with either DMSO or STM2457 (10 µM) for 24 h, followed by an RA treatment for another 3 days. Right: Box-whisker plots show quantification of neurite length. The median is indicated by a horizontal line, the boxes represent the 25$^{th}$ to 75$^{th}$ percentiles, the whiskers show the 10$^{th}$ to 90$^{th}$ percentiles, and any outliers beyond this range are displayed as individual dots. Data are from three independent experiments. Statistical significance was determined using a two-tailed unpaired *t*-test. Scale bar represents 50 µm. (**C**) Relative mRNA expression of *HOXC8*, *HOXC9* in SK-N-BE(2) cells treated with either DMSO or STM2457. *GAPDH* was used to normalize the qPCR data. Data are shown as mean±SEM of three replicates. Two-tailed unpaired *t*-test was used. (**D**) Stability of *HOXC8* and *HOXC9* transcripts detected by RT-qPCR after Actinomycin D (10 µg/ml) mediated transcription blocking for the time points indicated in SK-N-BE(2) cells treated with either DMSO or STM2457. Line plots presenting the quantification of remaining levels of *HOXC8* and *HOXC9* transcript at the indicated time points and $t_{½}$ values are also denoted. Two-way ANOVA with Šídák's multiple comparisons test was employed and data are shown as mean±SEM of three replicates. (**E**) Relative mRNA expression of *HOXC9* in hESC, tNCC, SAP, and SN. *GAPDH* was used to normalize the qPCR data. Data are presented as bar plots and shown as mean±SD of three replicates. Statistical analysis was performed using one-way ANOVA with Dunnett's multiple comparisons test. (**F**) Top enriched terms associated with upregulated genes in Flag-MYCN overexpressed (Dox+, from day 5 onwards) SN stage cells (D20 of differentiation) after DMSO or STM2457 (10 µM) treatment. STM2457 or DMSO was added from day 13 of differentiation. Enriched terms were identified using enrichGO, with *p*-values obtained through Fisher's exact test. (**G**) Top enriched terms associated with upregulated DEGs following Dox induced METTL3 KD and RA treatment in SK-N- BE(2) cells for 5 days. Enriched terms

were identified using enrichGO, with *p*-values obtained through Fisher's exact test. (**H**) Left: Representative IF showing expression of RPA32 (red) and gamma H2AX (green) expression in SN stage cells with simultaneous Flag-MYCN overexpression and METTL3 KD induced by Dox as indicated in panel (A) above. Right: Box-whisker plots show either RPA32 or gamma-H2AX intensity normalized to DAPI intensity. The median is indicated by a horizontal line, the boxes represent the $25^{th}$ to $75^{th}$ percentiles, the whiskers show the $10^{th}$ to $90^{th}$ percentiles, and any outliers beyond this range are displayed as individual dots. Data are from three independent experiments, with signal intensity measurements taken from over 70 cells. Statistical analysis was performed using a two-tailed unpaired *t*-test. Scale bar represents 10 µm. (**I**) Left: Dose-response inhibition matrix of STM2457 plus doxorubicin in COG-N-496h patient-derived xenograft (PDX) line, treated for 72 h with the combination of agents or agents alone; color gradients represent the % of inhibition in viability compared to the DMSO vehicle control treated cells. Right: Loewe synergy scores and *p*-value were calculated with SynergyFinder and are shown in the Synergy map. Scores >10 represent synergism in the activity of the drugs. Results shown are from the average of two independent experiments each with technical replicates. (**J**) Bar plots show the cell viability of tNCC treated with DMSO, STM2457, Doxorubicin, or a combination of STM2457 and Doxorubicin for 72 h. Data are presented as mean±SEM from three independent experiments. Statistical analysis was performed using one-way ANOVA with Dunnett's multiple comparisons test. (**K**) SK-N-BE(2)-GFP cells labeled with DiI (red dye) were injected into the perivitelline space of wild-type zebrafish, 48 h post fertilization. They received treatments with either solvent (0.2% DMSO), STM2457 100 µM, doxorubicin 5 µM, or a combination of both with the same doses for 72 h. Images were taken at 0 h and 72 h after treatment and were analyzed through ImageJ measuring tumor area using macro with the same settings on every image. Waterfall plots display the percent change in tumor size, while scatter dot plots show tumor growth as a fold change (72 h/0 h). The median is indicated by a horizontal line and a dotted line extends the control group's median for visual comparison. At least 19 zebrafish were used per condition. Statistical analysis was performed using one-way ANOVA followed by Dunnett’s multiple comparisons test. (**L**) Immunohistochemical staining of paraffin-embedded COG-N-415x PDX tumors grown in NSG mice. The mice were treated with vehicle, STM2457, doxorubicin, or a combination of both. Antibodies against Ki67, gamma-H2AX, or Phospho-RPA32 (Ser33) were used. Scale bar represents 100 µm.

**Appendix Supplementary Methods**

**Protein Stability Assay**

To inhibit protein synthesis, we employed the protein synthesis inhibitor cycloheximide (CHX) at a concentration of 50 µg/ml. Cells were collected at the specified time points, and protein was subsequently extracted. The METTL3 protein level was quantified through immunoblotting.

**Colony formation assay**

In total, $1x10^3$ SK-N-BE(2) shCtrl and shMETTL3 cells were plated in a 6-well plate. Dox induction was started after 24 h and Dox media was replaced every 2-3 days up to 14 days. Cells were then fixed with 10% formaldehyde for 10 min and then stained with 0.2% crystal violet solution for 1 h at RT. Excess crystal violet solution was carefully washed, and the plate was allowed to air dry and visualized using ChemiDoc.

**Polysome profiling**

Cells were treated with the addition of CHX (100 µg/ml) to the cell medium, followed by incubation at 37°C for 10 min. Cells were then washed twice with ice-cold PBS supplemented with CHX (100 µg/ml). After removal of PBS, cells were scraped and collected in 1ml ice-cold lysis buffer (20 mM Hepes pH 7.5, 50 mM KCl, 10 mM $Mg(CH_3COO)_2$, 1.5 mM Dithiothreitol (DTT; Merck), 100 µg/mL CHX, cOmplete EDTA-free Protease Inhibitor Cocktail (Merck), RNaseOUT recombinant ribonuclease inhibitor (200 U/mL; Thermo Fisher Scientific) and IGEPAL CA-630 (0.7 %; Merck)) and lysed for 30 minutes on ice. Lysate was cleared by 14000 rcf centrifugation for 5 min at 4°C. RNA content was quantified with a NanoDrop spectrophotometer (Thermo Fisher Scientific) and similar amounts of A260 units were loaded on a linear sucrose gradient (10-50% (w/v) Sucrose (Merck) 20 mM Hepes pH 7.5, 50 mM KCl, 10 mM $Mg(CH_3COO)_2$, 1.5 mM DTT, 100 µg/mL CHX, cOmplete™ EDTA-free Protease Inhibitor Cocktail, RNaseOUT recombinant ribonuclease inhibitor (4 U/mL)). Gradients were centrifuged at 36000rpm for 2 hrs at 4°C followed by polysome profiling and fraction collection using a BioComp Gradient Station (BioComp). Fractions were collected in volumes of 500µl, to which 750µl TRIzol Reagent was added before freezing at -80°C. RNA was isolated from polysome fractions and followed by RT-qPCR as mentioned before.

**Nascent RNA labeling assay**

SHEP$^{MYCN}$ cells were seeded on coverslips and allowed to grow overnight. The following day, MYCN overexpression was induced by adding Dox for 24 h. Subsequently, the cells were treated with 300 nM Flavopiridol (FP) for 1 h in the presence or absence of Dox, followed by the addition of 5-ethynyl uridine (EU) for 30 min. The cells were then fixed and permeabilized as previously described. RNA labeled with 5-ethynyl uridine was detected using the Click-iT RNA Imaging Kit (Life Technologies, catalog number C10329), following the manufacturer's protocol.

**dCasRx FTO system**

SK-N-BE(2) cells expressing CasRx/ dCasRx-FTO$^{WT}$ (FTO wild type)/ dCasRx-FTO$^{Mutant}$ (FTO catalytically dead mutant-H231A and D233A) with HOXC9 and non-template control (NTC) gRNAs were made as previously described (Vaid *et al*, 2024).

**Zebrafish xenograft tumors**

100-300 GFP transduced SK-N-BE(2) cells were injected into the perivitelline space of 48 h post fertilization embryos. The zebrafish embryos were exposed to either 0.2% DMSO, 100 µM STM2457, 5 µM doxorubicin, or a combination of 100 µM STM2457 and 5 µM doxorubicin in E3 water containing 30 mg/L phenylthiourea (PTU) for 72 h. Images of the zebrafish embryos were taken at 2 days post fertilization (dpf) (day 0 after treatment) and 5 dpf (day 3 after treatment) using the Acquifer imaging machine (Bruker). The embryos were kept at 33°C during imaging. The images from the green channel were quantified using analyzed particles in Image J (v. 2.14.0/1.54f) using a macro that we developed to measure the tumor areas. The same settings were used on all images. According to the EU directive 2010/63/EU and the Swedish legislation on animal experimentation (L150), zebrafish embryos younger than 5 days are not considered research animals. The breeding animals were kept under ethical license #15591/2023.

**Immunohistochemistry**

Paraffin-embedded COG-N-415x PDX tumors were embedded in paraffin, sectioned, and subjected to immunostaining. Citrate buffer pH 6 (Sigma, #C-9999) was used for antigen retrieval. Primary antibodies that were used were anti-gamma H2A.X (phospho S139) (1:5000), anti-Ki67 (1:100), and anti-Phospho-RPA32 (1:5000), incubated at 4°C overnight. The sections were washed with TBST (Tris Buffered Saline with 0.1% Tween-20) and incubated with HRP-conjugated anti-rabbit secondary antibody (ImmPRESS Polymer Kit; Vector Laboratories, # MP-7451) for 30 min. ImmPACT DAB

(Vector Laboratories, #SK-4105) was used for development, and Mayer's Hematoxylin for counterstaining.

**Chemical synthesis of STM2457**

***N*-[(6-{[(cyclohexylmethyl)amino]methyl}imidazo[1,2-a]pyridin-2-yl)methyl]-4-oxo-4*H*pyrido[1,2-a]pyrimidine-2-carboxamide – STM2457**

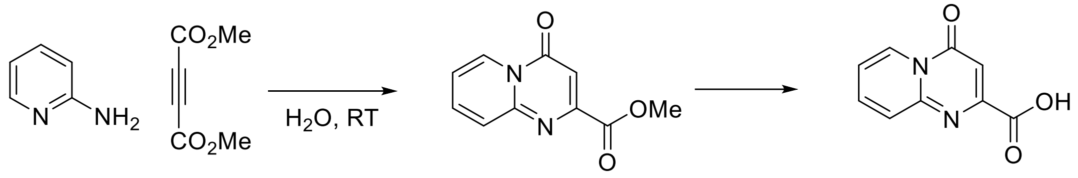


**Scheme 1. Synthesis of 4-oxo-4*H*-pyrido[1,2-a]pyrimidine-2-carboxylic acid**

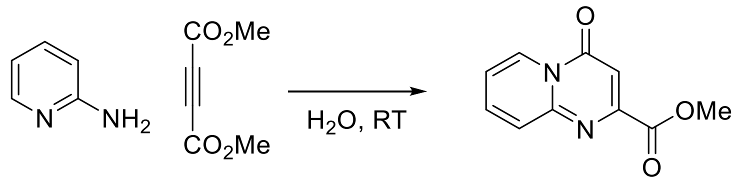


**Step 1: Methyl 4-oxopyrido[1,2-a]pyrimidine-2-carboxylate.**

Dimethyl but-2-ynedioate (16 mL, 0.126 mol) was added slowly to a vigorously stirred solution of pyridin-2-amine (10.00 g, 0.105 mol) in water (1000 mL), and the reaction mixture was stirred at room temperature for 18 h. After completion of the reaction, the reaction mixture was extracted with DCM (3x100 mL), the organic layer was collected and dried over $MgSO_4$, and volatiles were removed under reduced pressure. The crude product was purified by column chromatography on silica gel using eluent ethyl acetate in hexane to obtain methyl 4-oxopyrido[1,2-a]pyrimidine-2-carboxylate (8.7 g, 40%) as an off-white solid.

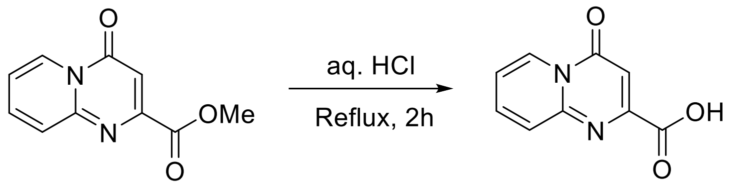


**Step 2: 4-oxo-4*H*-pyrido[1,2-a]pyrimidine-2-carboxylic acid.**

Methyl 4-oxopyrido[1,2-a]pyrimidine-2-carboxylate (8.7 g, 42.9 mmol) was dissolved in aq. HCl (~8M, 9.5 mL) at room temperature, and the solution was heated at reflux for 2 h. The mixture was cooled to room temperature and the precipitate was collected by filtration and dried on the filter to give the title compound 4-oxo-4*H*-pyrido[1,2-a]pyrimidine-2-carboxylic acid. (6.5 g, 79 %). $^1$H NMR (600 MHz,

DMSO-$d_6$) $\delta$ 9.00 (d, J = 6.3 Hz, 1H), 8.05 (ddd, J = 8.6, 6.7, 1.6 Hz, 1H), 7.83 (dt, J = 8.8, 1.1 Hz, 1H), 7.46 (td, J = 6.9, 1.4 Hz, 1H), 6.87 (s, 1H).

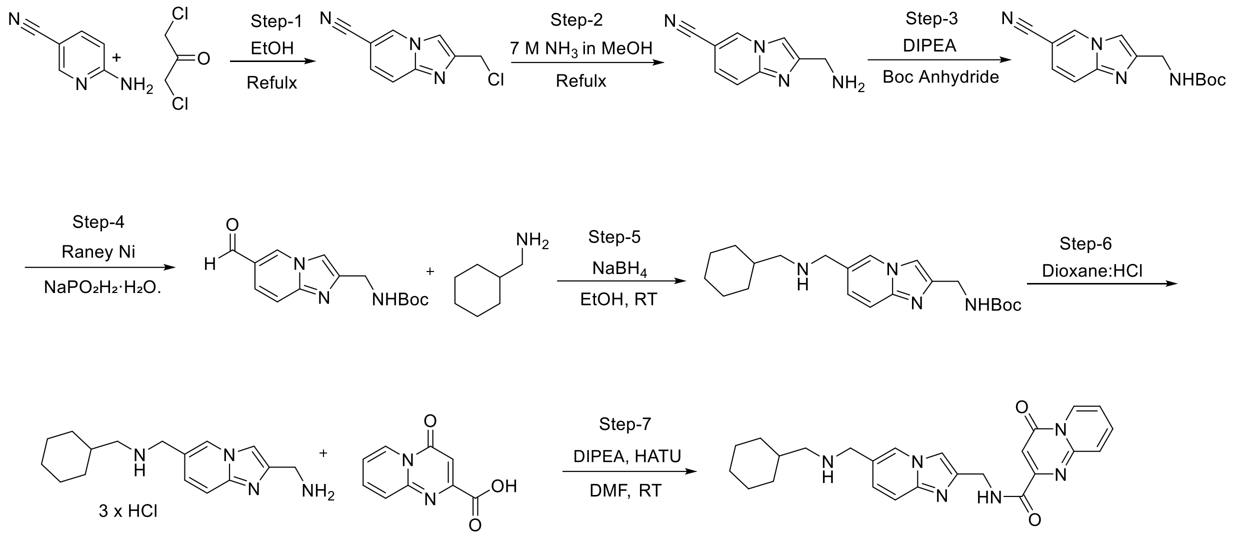


**Scheme 2: Synthesis of STM 2457**

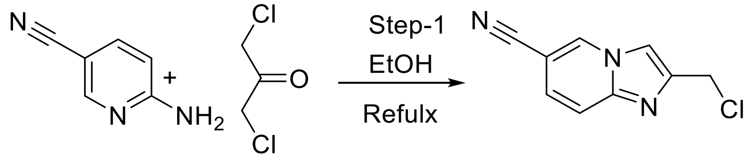


**Step 1: 2-(Chloromethyl)imidazo[1,2-*a*]pyridine-6-carbonitrile**

To a stirred solution of 6-aminonicotinonitrile (15 g, 126 mmol) in ethanol (40 mL), 1,3-dichloropropan-2-one (45g, 378 mmol) was added under nitrogen atmosphere and stirred for 16 h at 60 °C. After completion of the reaction, the reaction mixture was poured into ice-cold saturated solution of sodium bicarbonate (160 mL), then filtered to obtain a crude product. The crude product was purified by column chromatography on silica gel using eluent ethyl acetate in hexane to obtain 2-(Chloromethyl)imidazo[1,2-a]pyridine-6-carbonitrile (20 g, 104 mmol, 83 % yield). $^1$H NMR (600 MHz, $CDCl_3$) $\delta$ 8.53 (dd, $J$ = 1.7, 1.0 Hz, 1H), 7.71 (d, $J$ = 0.8 Hz, 1H), 7.64 (dt, $J$ = 9.4, 0.9 Hz, 1H), 7.31 – 7.26 (m, 1H), 4.74 (d, $J$ = 0.7 Hz, 2H).

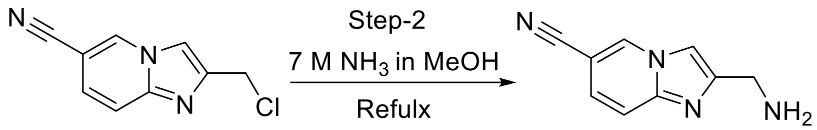


**Step 2: 2-(Aminomethyl)imidazo[1,2-*a*]pyridine-6-carbonitrile**

To a stirred solution of 2-(chloromethyl)imidazo[l,2-a]pyridine-6-carbonitrile (5 g, 26.17 mmol) in methanol (50 mL), (7N) $NH_3$ in methanol (4.47 mL, 67.6 mmol) was added and stirred for 4 h at 80 °C. The volatiles were removed under reduced pressure to obtain a 2-(aminomethyl)imidazo[1,2-a]pyridine-6-carbonitrile (4.2 g, 83 % yield).

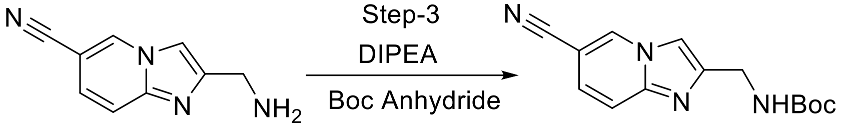


**Step 3: *tert*-Butyl ((6-cyanoimidazo[1,2-*a*]pyridin-2-yl)methyl)carbamate**.

A suspension of 2-(aminomethyl)imidazo[1,2-*a*]pyridine-6-carbonitrile (4.2 g, 24.41 mmol) and *N*-ethyl-*N*-isopropyl-propan-2-amine (3.7 mL, 26.0 mmol) in THF (35 mL) and DCM (35 mL) was treated with *N*,*N*-dimethylpyridin-4-amine (0.085 g, 0.697 mmol) and *tert*-butoxy carbonyl *tert*-butyl carbonate (3.40 g, 15.6 mmol) at 0 °C and the mixture was allowed to warm to room temperature for 10 h. The reaction mixture was concentrated under vacuum, the residue was suspended in water- $CH_3CN$ (1:1, 15mL), and sonicated for 20 min. The solid was filtered and then treated with water-MeCN again. The obtained solid was dried under vacuum to give the title compound as an off-white solid, which was further purified by the column chromatography on silica gel using eluent ethyl acetate in hexane to obtain *tert*-Butyl ((6-cyanoimidazo[1,2-a]pyridin-2-yl)methyl)carbamate (5.1 g, 86% yield).

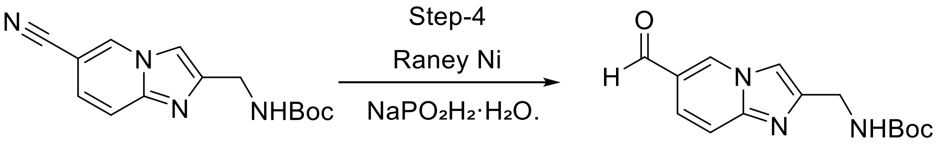


**Step 4: *tert*-Butyl ((6-formylimidazo[1,2-*a*]pyridin-2-yl)methyl)carbamate.**

A suspension of *tert*-butyl *N*-[(6-cyanoimidazo[1,2-a]pyridin-2- yl)methyl]carbamate (5.00 g, 18.4 mmol) and sodium phosphinate hydrate (15.57 g, 0.147 mol) in a mixture of water (50 mL), Pyridine (100 mL) and Acetic acid (50 mL) was treated with Raney nickel (50%, 18.97 g, 0.162 mol) and the mixture was heated at 100 °C under a nitrogen atmosphere for 1 h. The Raney nickel was removed by hot filtration through a bed of Celite (washing with water, followed by methanol). The filtrate was concentrated under

a vacuum to remove the methanol and the blue solution was extracted with DCM (50 mL x 4). The extracts evaporated under a vacuum to afford a beige gum which was triturated with water to furnish a white solid. The solid was collected by filtration, washed with water followed by ether, and dried under vacuum overnight to provide the title compound *tert*-Butyl ((6-formylimidazo[1,2-a]pyridin-2-yl)methyl)carbamate (4.13 g, 82%) as an off white solid. $^1$H NMR (600 MHz, DMSO-$d_6$) $\delta$ 9.27 (s, 1H), 7.78 (s, 1H), 7.59 (dt, $J$ = 9.3, 0.9 Hz, 1H), 7.42 (dd, $J$ = 9.3, 1.7 Hz, 1H), 7.38 (t, $J$ = 6.1 Hz, 1H), 6.09 (s, 1H), 4.23 (d, $J$ = 6.0 Hz, 2H), 1.37 (s, 10H).

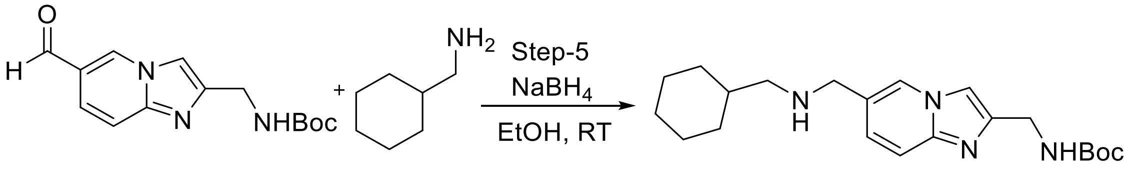


**Step 5: *tert*-Butyl ((6-(((cyclohexylmethyl)amino)methyl)imidazo[1,2-*a*]pyridin-2-yl)methyl)carbamate.**

A solution of *tert*-butyl *N*-[(6-formylimidazo[1,2-a]pyridin-2-yl)methyl]carbamate (340 mg, 0.988 mmol) and cyclohexyl methanamine (224 mg, 1.98 mmol) in ethanol (6.8 mL) was stirred at 50 °C for 1 h. The reaction mixture was cooled to 0 °C and $NaBH_4$ (75 mg, 1.98 mmol) was added in portions. The reaction mixture was allowed to warm to room temperature and stirring was continued for 2 h. The reaction mixture was partitioned between DCM (80 ml) and saturated aqueous sodium hydrogen carbonate (30 ml). The organic layer was separated, washed with brine (30 ml), dried over $MgSO_4$, and concentrated under reduced pressure. The residue was purified by column chromatography on silica gel with 0-20% methanol in DCM to afford the title compound *tert*-Butyl ((6-(((cyclohexylmethyl)amino)methyl)imidazo[1,2-a]pyridin-2-yl)methyl)carbamate (360 mg, 97%) as a yellow oil. $^1$H NMR (600 MHz, DMSO-$d_6$) $\delta$ 8.37 (s, 1H), 7.65 (s, 1H), 7.39 (d, $J$ = 9.2 Hz, 1H), 7.27 (t, $J$ = 6.2 Hz, 1H), 7.19 (dd, $J$ = 9.2, 1.7 Hz, 1H), 4.21 (d, $J$ = 6.0 Hz, 2H), 3.63 (d, $J$ = 1.0 Hz, 2H), 2.31 (d, $J$ = 6.6 Hz, 2H), 1.75 – 1.71 (m, 2H), 1.66 – 1.59 (m, 3H), 1.40 (s, 9H), 1.37-1.34 (m, 1H), 1.26 – 1.01 (m, 4H), 0.87– 0.80 (m, 2H).

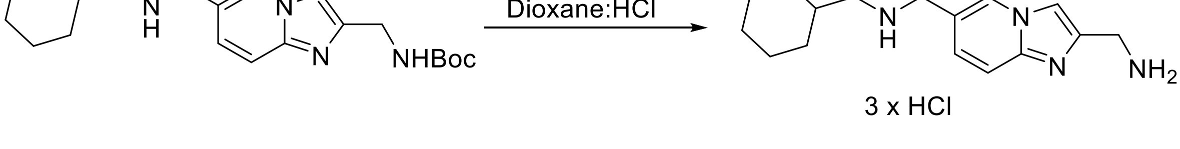

**Step 6. 1-(2-(Aminomethyl)imidazo[1,2-*a*]pyridin-6-yl)-*N*-(cyclohexylmethyl)methanamine** To a solution of *tert*-butyl *N*-[[6-[(cyclohexylmethylamino)methyl]imidazo[1,2-a]pyridin-2-yl]methyl]carbamate (360 mg, 0.908 mmol) in methanol (4.1379 mL) was added HCl (4M in dioxane, 2.4 mL, 9.6 mmol) at room temperature The solution was stirred at 50 °C for 1 h. The solution was cooled to room temperature, and concentrated to dryness under reduced pressure. The residue (pale yellow glass) was dissolved in methanol (~3 mL) and diethyl ether (~ 20 mL) was added drop-wise to the stirred solution. The resulting precipitate was collected by filtration and dried in the vacuum oven at 40 °C for 4 h to afford the title compound 1-(2-(Aminomethyl)imidazo[1,2-a]pyridin-6-yl)-*N*-(cyclohexylmethyl)methanamine trihydrochloride salt (339 mg, 95%) as white solid.

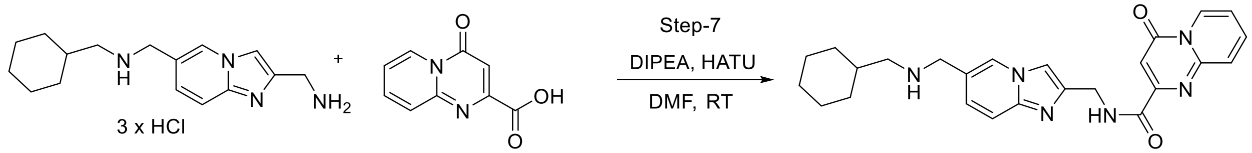


**Step 7. *N*-((6-(((Cyclohexylmethyl)amino)methyl)imidazo[1,2-*a*]pyridin-2-yl)methyl)-2-oxo-2*H*-pyrido[1,2-*a*]pyrimidine-4-carboxamide**

A solution of 4-oxopyrido[1,2-a]pyrimidine-2-carboxylic acid (242 mg, 1.2 mmol), HATU (724 mg, 1.9 mmol), and 1-(2-(aminomethyl)imidazo[1,2-a]pyridin-6-yl)-*N*-(cyclohexylmethyl) methanamine trihydrochloride (414 mg, 1.5 mmol) in DMF (7 mL) was treated with *N*-ethyl-*N*-isopropyl-propan-2-amine (1.1 mL, 8 mmol) and the suspension was stirred at room temperature for 16 h. The suspension was concentrated under vacuum and the residue was diluted with water. The solids were then collected by filtration, washed with water followed by ether, and dried under vacuum to give the title compound (203 mg, 36%) as an off-white solid. $^{1}$H NMR (600 MHz, DMSO-$D_6$) $\delta$ 9.14 (t, $J$ = 6.0 Hz, 1H), 9.02 (d, $J$ = 6.5 Hz, 1H), 8.37 (s, 1H), 8.07 (ddd, $J$ = 8.6, 6.7, 1.6 Hz, 1H), 7.80 (dt, $J$ = 8.9, 1.1 Hz, 1H), 7.78 – 7.76 (m, 1H), 7.51 – 7.41 (m, 2H), 7.21 (dd, $J$ = 9.3, 1.7 Hz, 1H), 6.90 (s, 1H), 4.61 (d, $J$ = 5.9 Hz, 2H), 3.64 (s, 2H), 2.30 (d, $J$ = 6.6 Hz, 2H), 1.76 – 1.70 (m, 2H), 1.66 – 1.58 (m, 3H), 1.43 – 1.34 (m, 1H), 1.19 – 1.08 (m, 3H), 0.86 – 0.81 (m, 2H). $^{13}$C NMR (150 MHz, DMSO-$D_6$) $\delta$ 162.66, 157.85, 153.68, 150.45, 143.70, 143.39, 138.42, 127.36, 126.18, 126.03, 124.92, 124.34, 117.21, 115.75, 110.08, 100.72, 55.12, 50.13, 37.67, 37.50, 31.01, 26.25, 25.58.

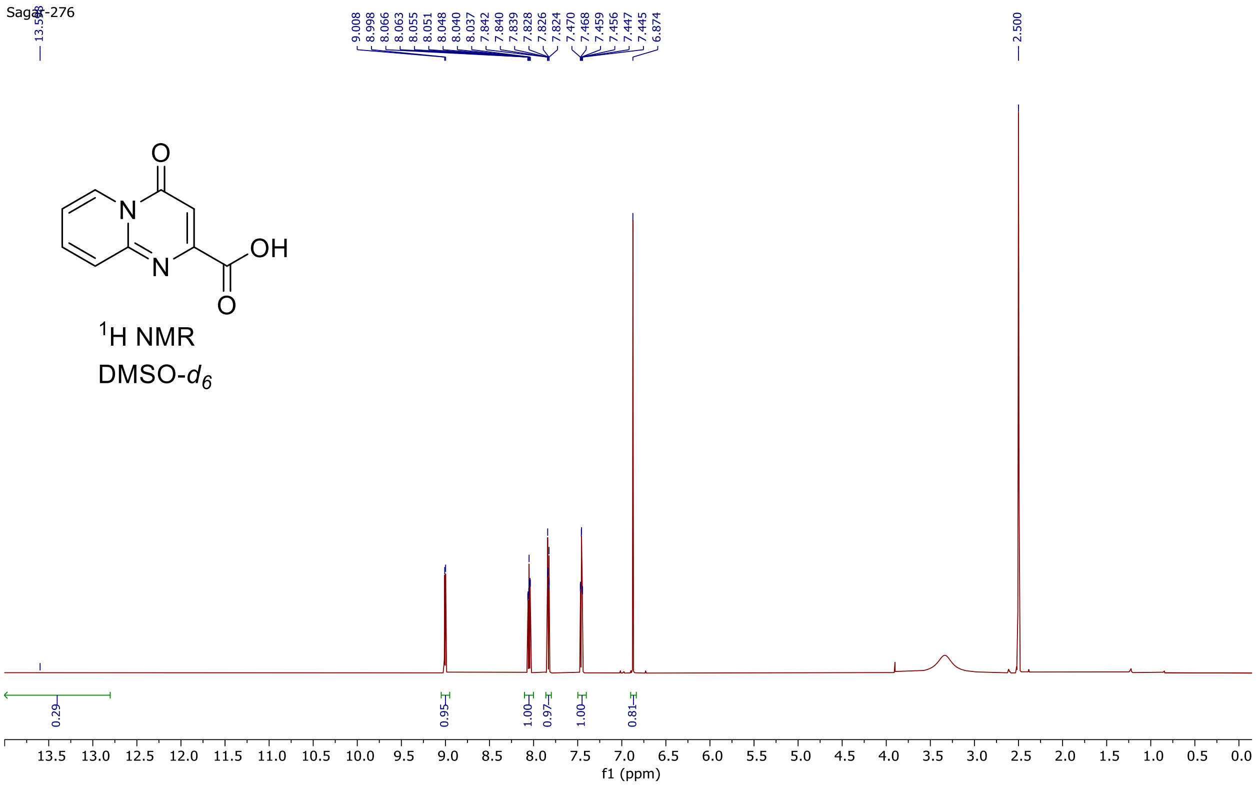
Sagar-276
OH
O
N
$^1$H NMR
DMSO-$d_6$
f1 (ppm)

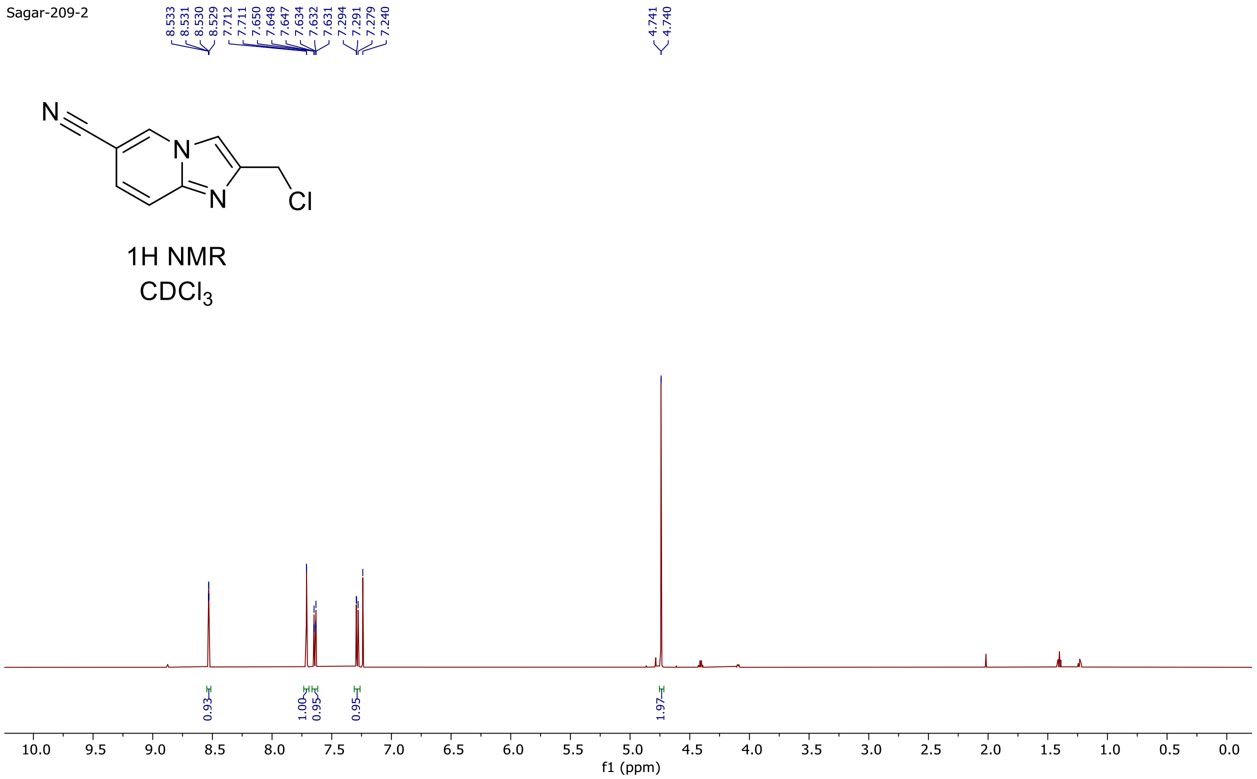
Sagar-209-2
N
Cl
1H NMR
$CDCl_3$
f1 (ppm)

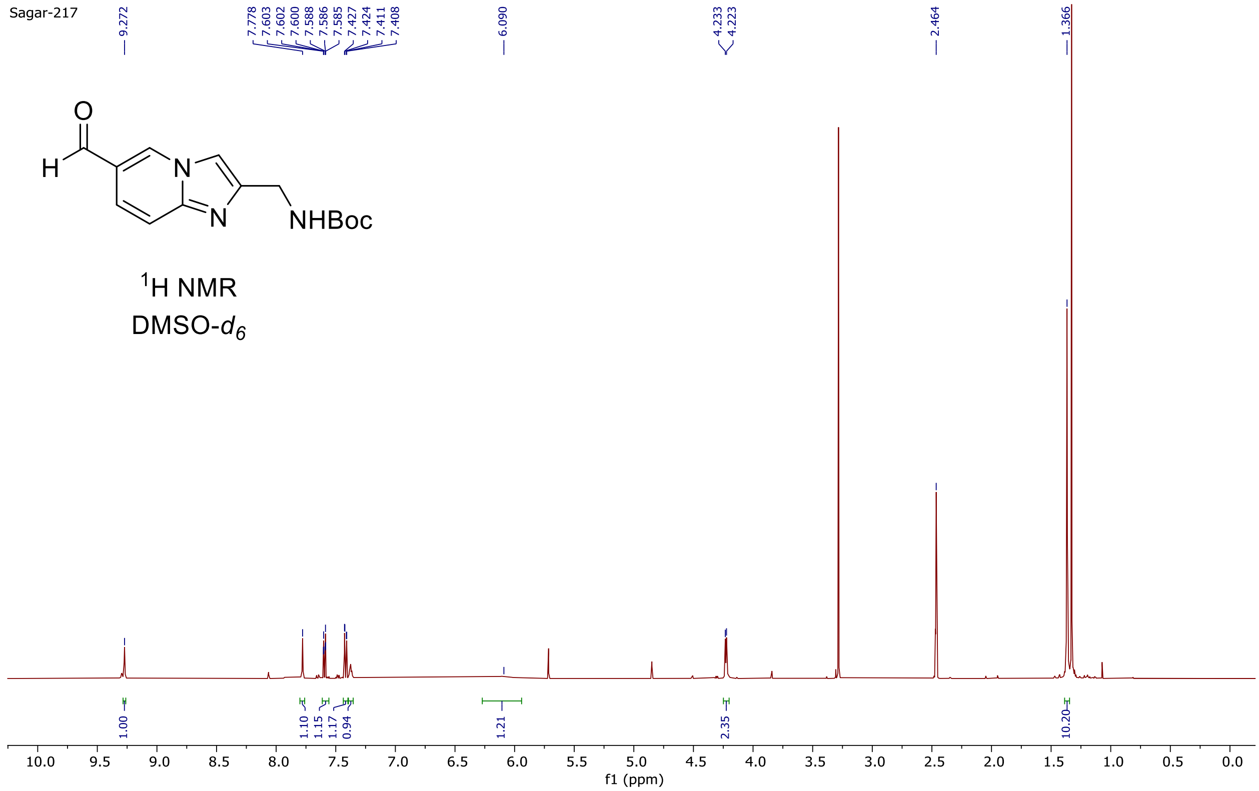
Sagar-217
NHBoc
¹H NMR
DMSO-d6
f1 (ppm)

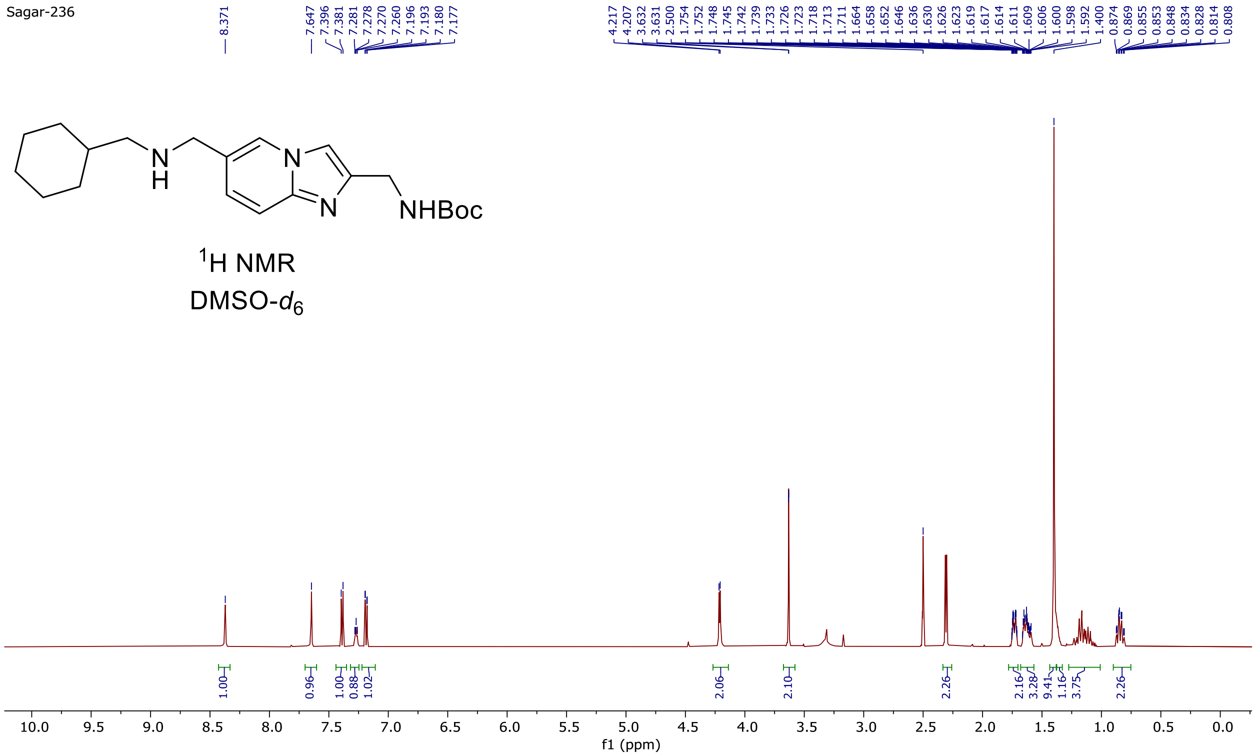
Sagar-236
NHBoc
¹H NMR
DMSO-d6
f1 (ppm)

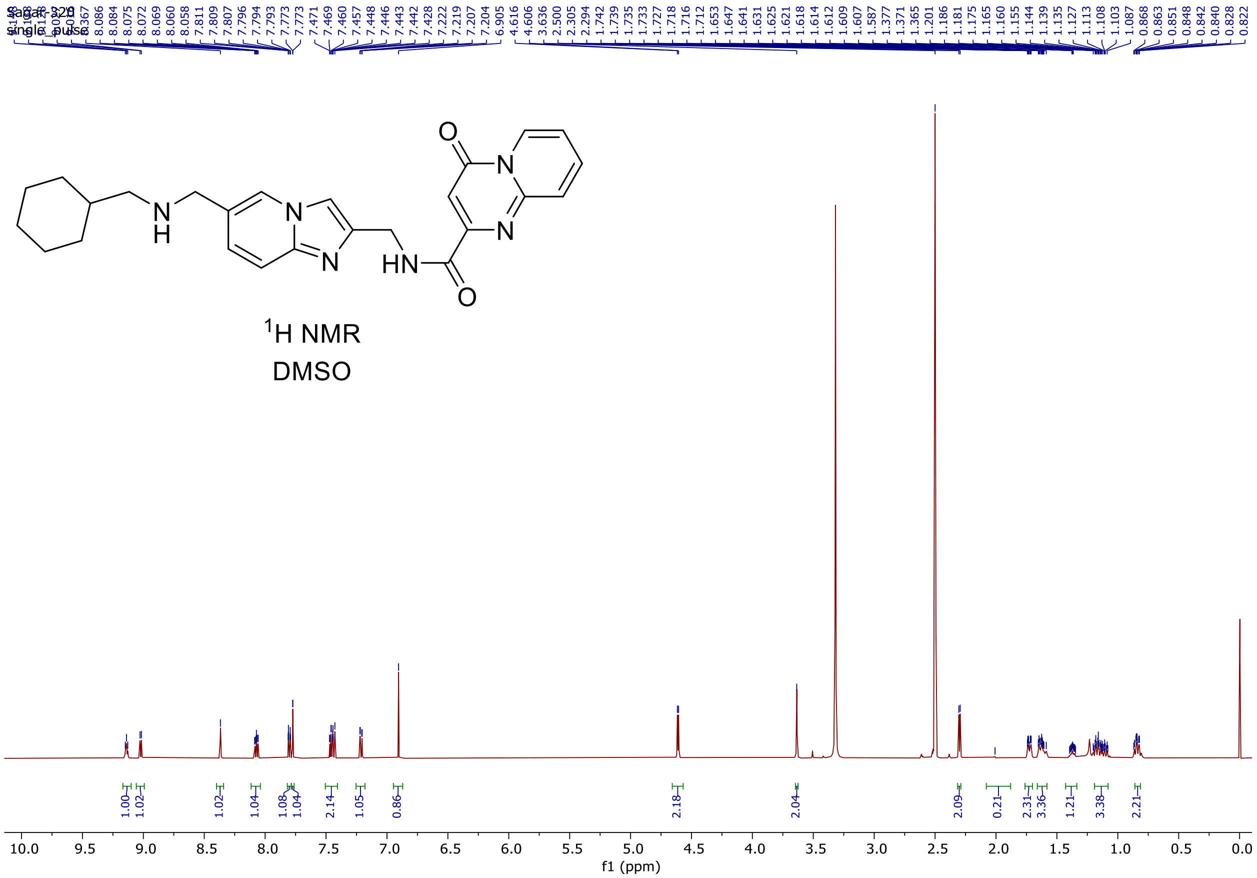

Sagar-320
single_pulse
$^{1}$H NMR
DMSO
8.086 8.084 8.075 8.072 8.069 8.060 8.058 7.811 7.809 7.807 7.796 7.794 7.793 7.773 7.773 7.471 7.469 7.460 7.457 7.448 7.446 7.443 7.442 7.428 7.222 7.219 7.207 7.204 6.905 4.616 4.606 3.636 2.500 2.305 2.294 1.742 1.739 1.735 1.733 1.727 1.718 1.716 1.712 1.653 1.647 1.641 1.631 1.625 1.621 1.618 1.614 1.612 1.609 1.607 1.587 1.377 1.371 1.365 1.201 1.186 1.181 1.175 1.165 1.160 1.155 1.144 1.139 1.135 1.127 1.113 1.108 1.103 1.087 0.868 0.863 0.851 0.848 0.842 0.840 0.828 0.822
1.00 1.02 1.02 1.04 1.08 1.04 2.14 1.05 0.86 2.18 2.04 2.09 0.21 2.31 3.36 1.21 3.38 2.21
f1 (ppm)


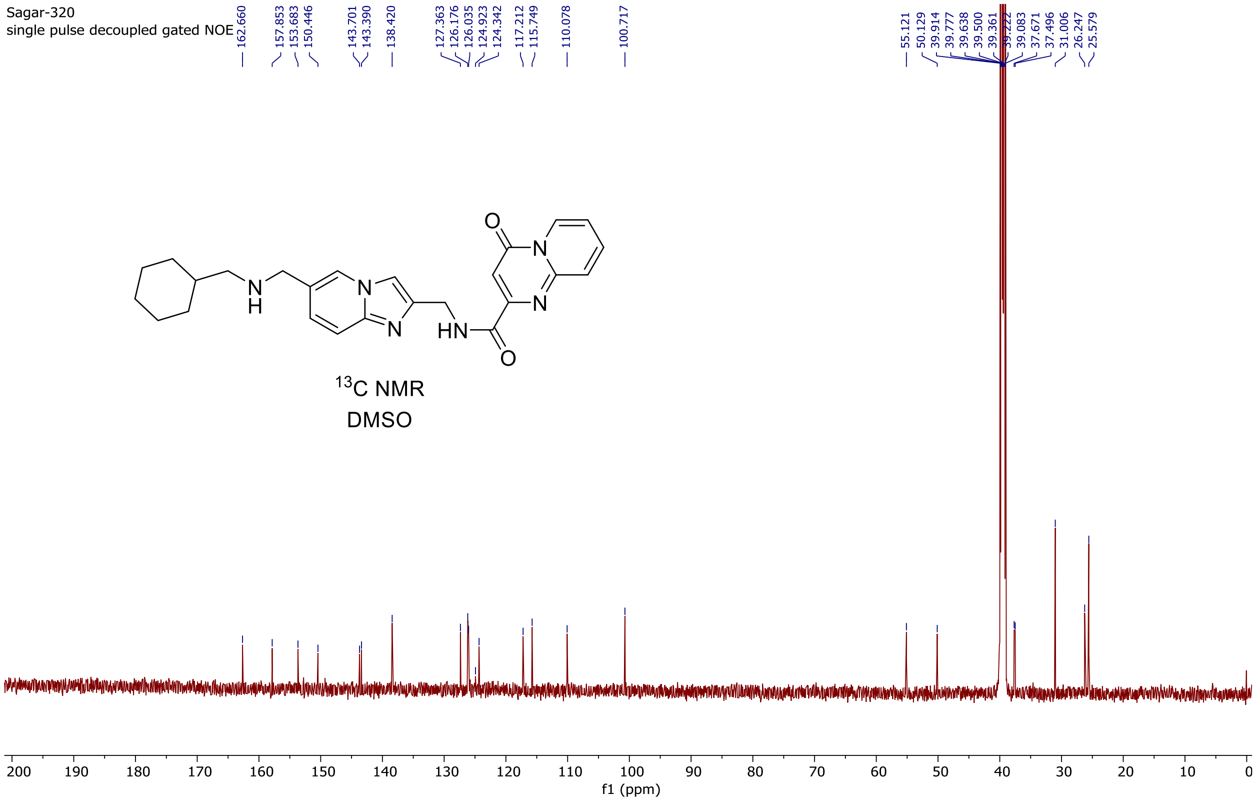

Sagar-320
single pulse decoupled gated NOE
162.660 157.853 153.683 150.446 143.701 143.390 138.420 127.363 126.176 126.035 124.923 124.342 117.212 115.749 110.078 100.717 55.121 50.129 39.914 39.777 39.638 39.500 39.361 39.222 39.083 37.671 37.496 31.006 26.247 25.579
$^{13}$C NMR
DMSO
f1 (ppm)

## References

Vaid R, Thombare K, Mendez A, Burgos-Panadero R, Djos A, Jachimowicz D, Lundberg KI, Bartenhagen C, Kumar N, Tummler C *et al* (2024) METTL3 drives telomere targeting of TERRA lncRNA through m6A-dependent R-loop formation: a therapeutic target for ALT-positive neuroblastoma. *Nucleic Acids Res*

Wang X, Choi JH, Ding J, Yang L, Ngoka LC, Lee EJ, Zha Y, Mao L, Jin B, Ren M *et al* (2013) HOXC9 directly regulates distinct sets of genes to coordinate diverse cellular processes during neuronal differentiation. *BMC Genomics* 14: 830
