# Supplementary figures and images for: METTL3/MYCN cooperation drives neural crest differentiation and provides therapeutic vulnerability in neuroblastoma
Source: EMBO J. 2024 Nov 11;43(24):6310–35. doi: 10.1038/s44318-024-00299-8 (PMC11649786; doi:10.1038/s44318-024-00299-8)

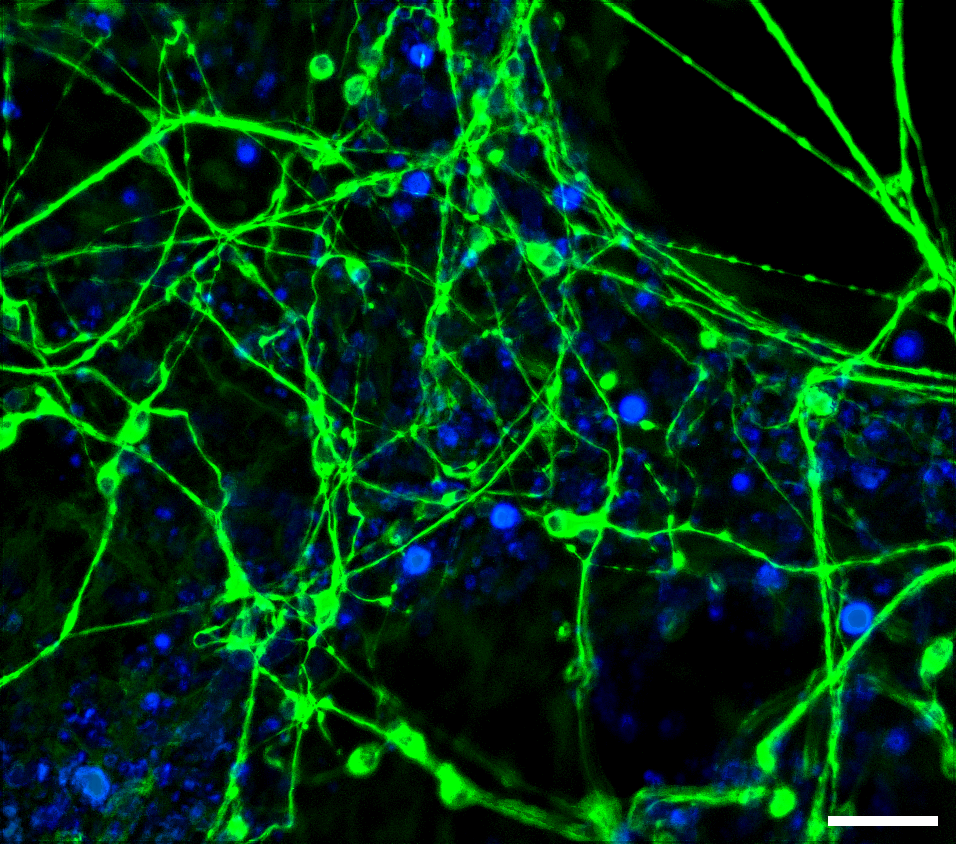

Supplement: Supplementary file 4 — Source data Fig. 1 [file 44318_2024_299_MOESM4_ESM.zip › Figure 1/1B/ES H9_PRPH.tif]

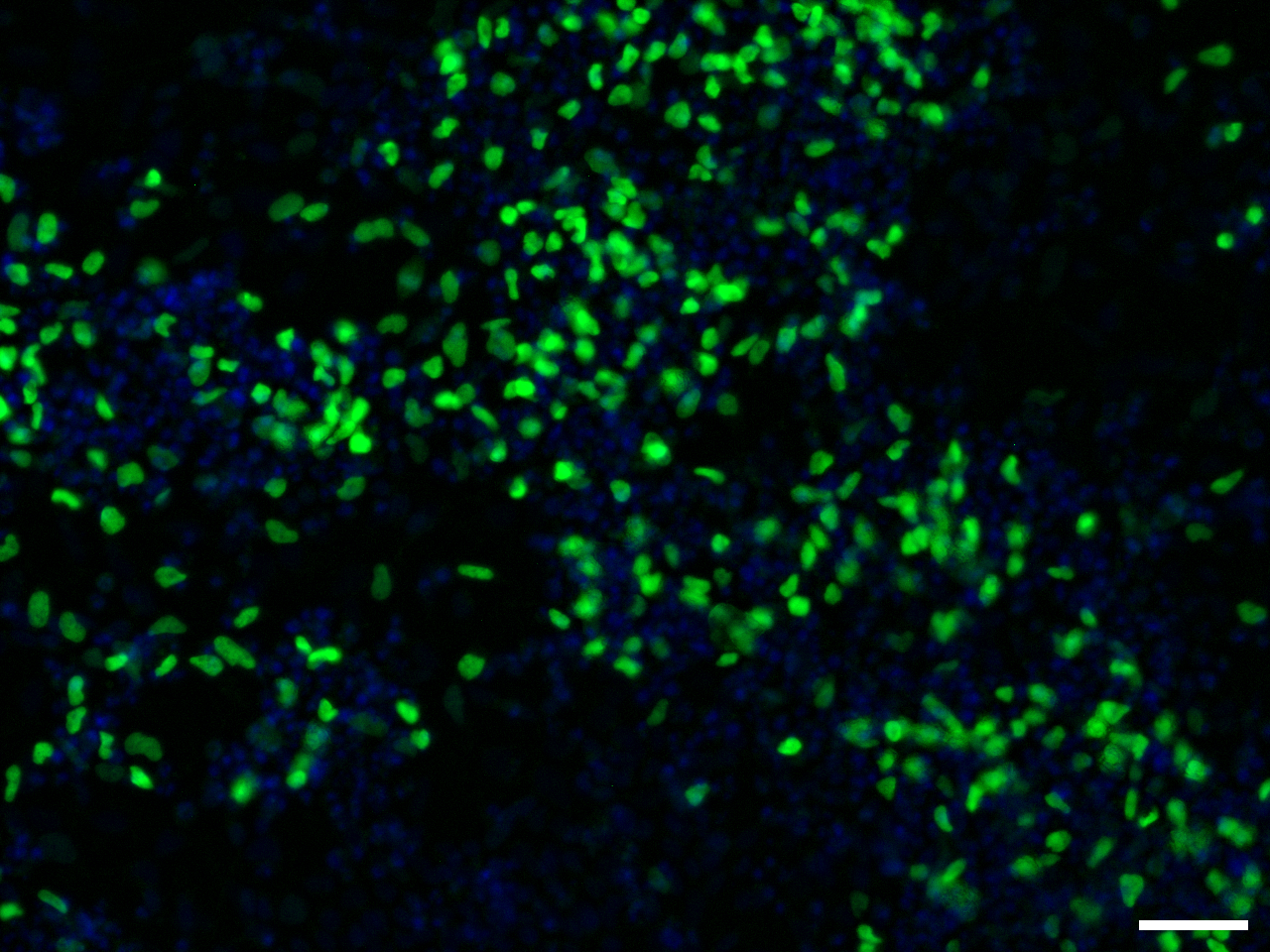

Supplement: Supplementary file 4 — Source data Fig. 1 [file 44318_2024_299_MOESM4_ESM.zip › Figure 1/1B/SAP_PHOX2B.tif]

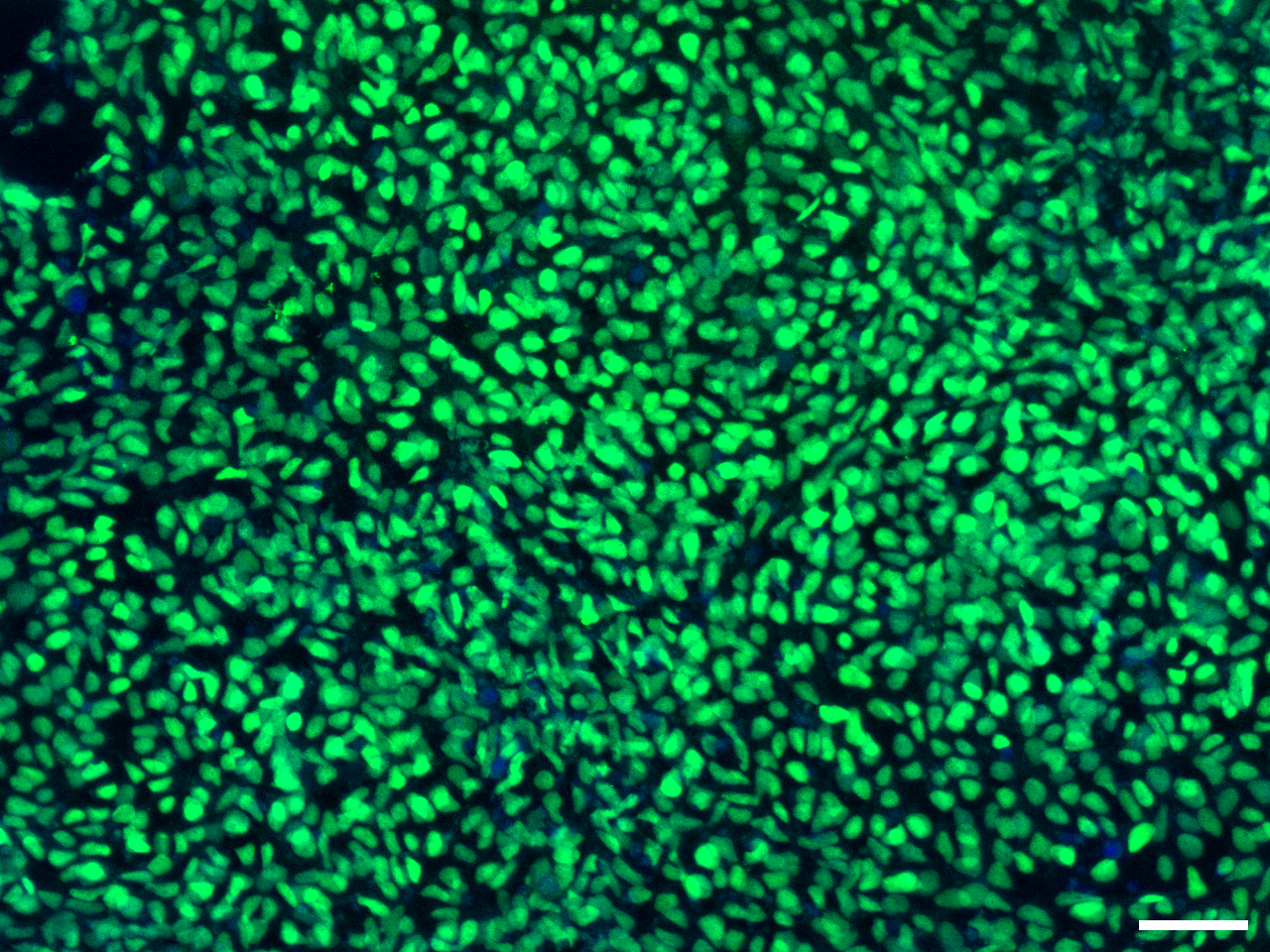

Supplement: Supplementary file 4 — Source data Fig. 1 [file 44318_2024_299_MOESM4_ESM.zip › Figure 1/1B/tNCC_HOXC9.tif]

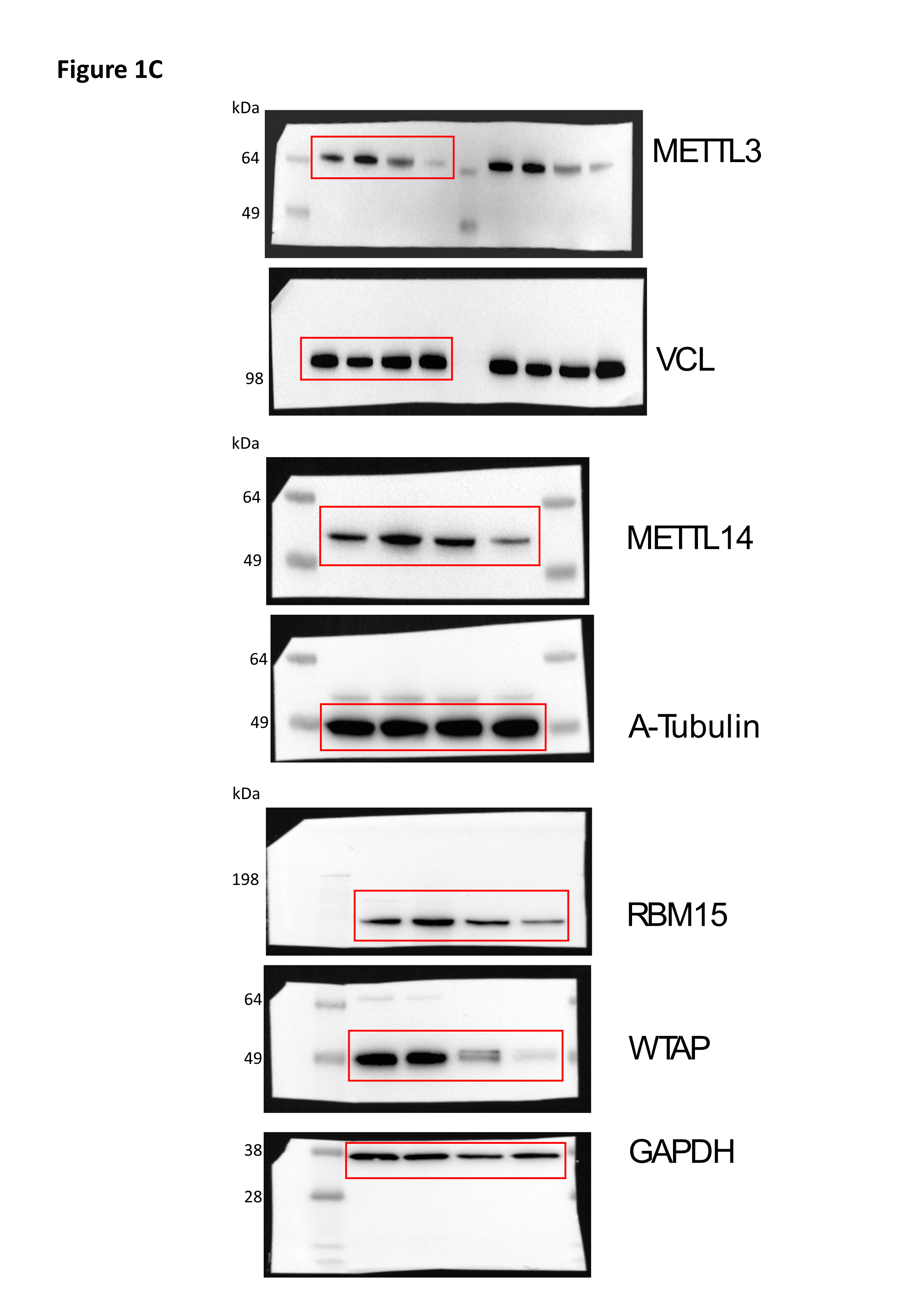

Supplement: Supplementary file 4 — Source data Fig. 1 [file 44318_2024_299_MOESM4_ESM.zip › Figure 1/1C/Source data for Figure 1C.tiff]

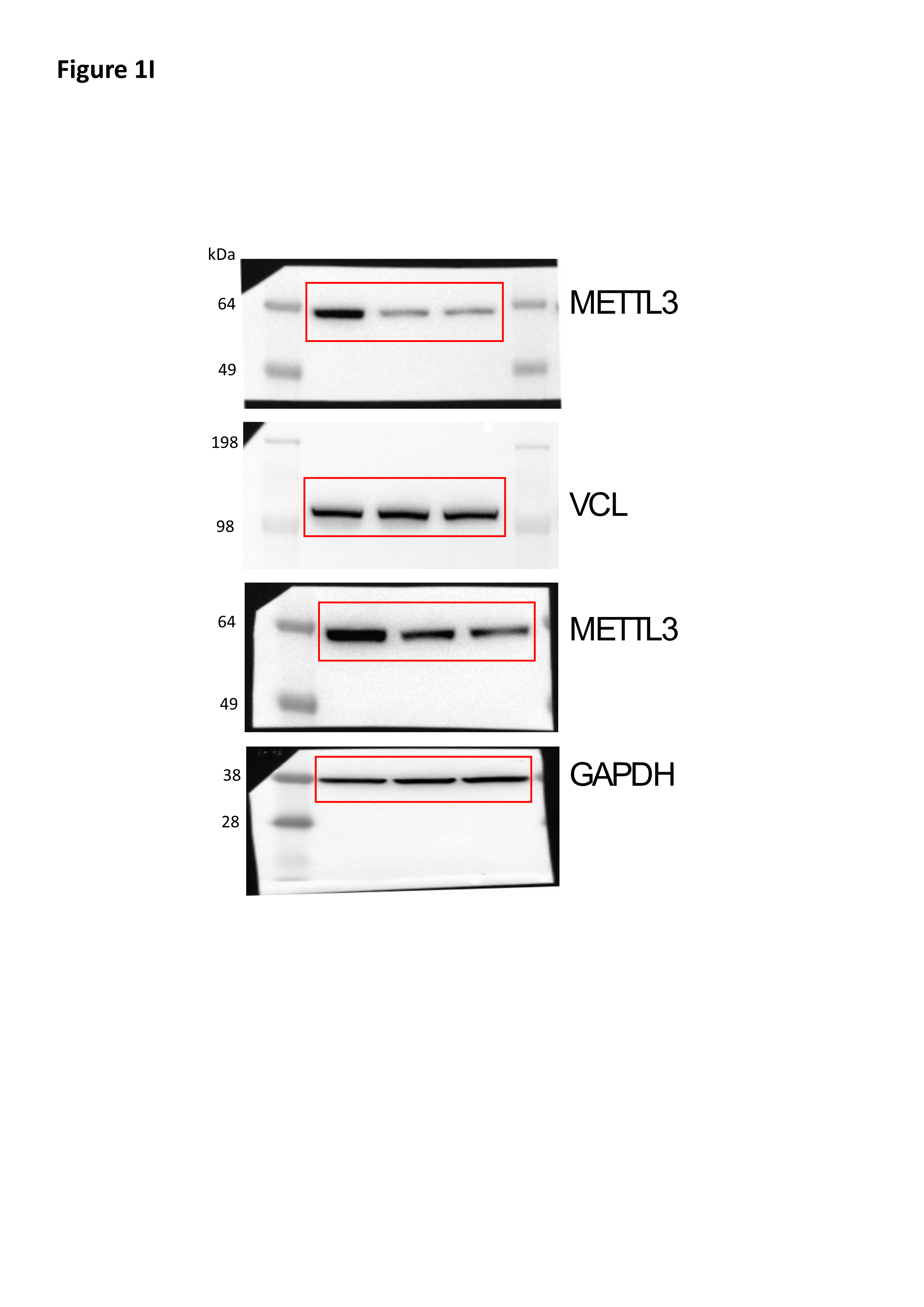

Supplement: Supplementary file 4 — Source data Fig. 1 [file 44318_2024_299_MOESM4_ESM.zip › Figure 1/1I/Source data for Figure 1I.tiff]

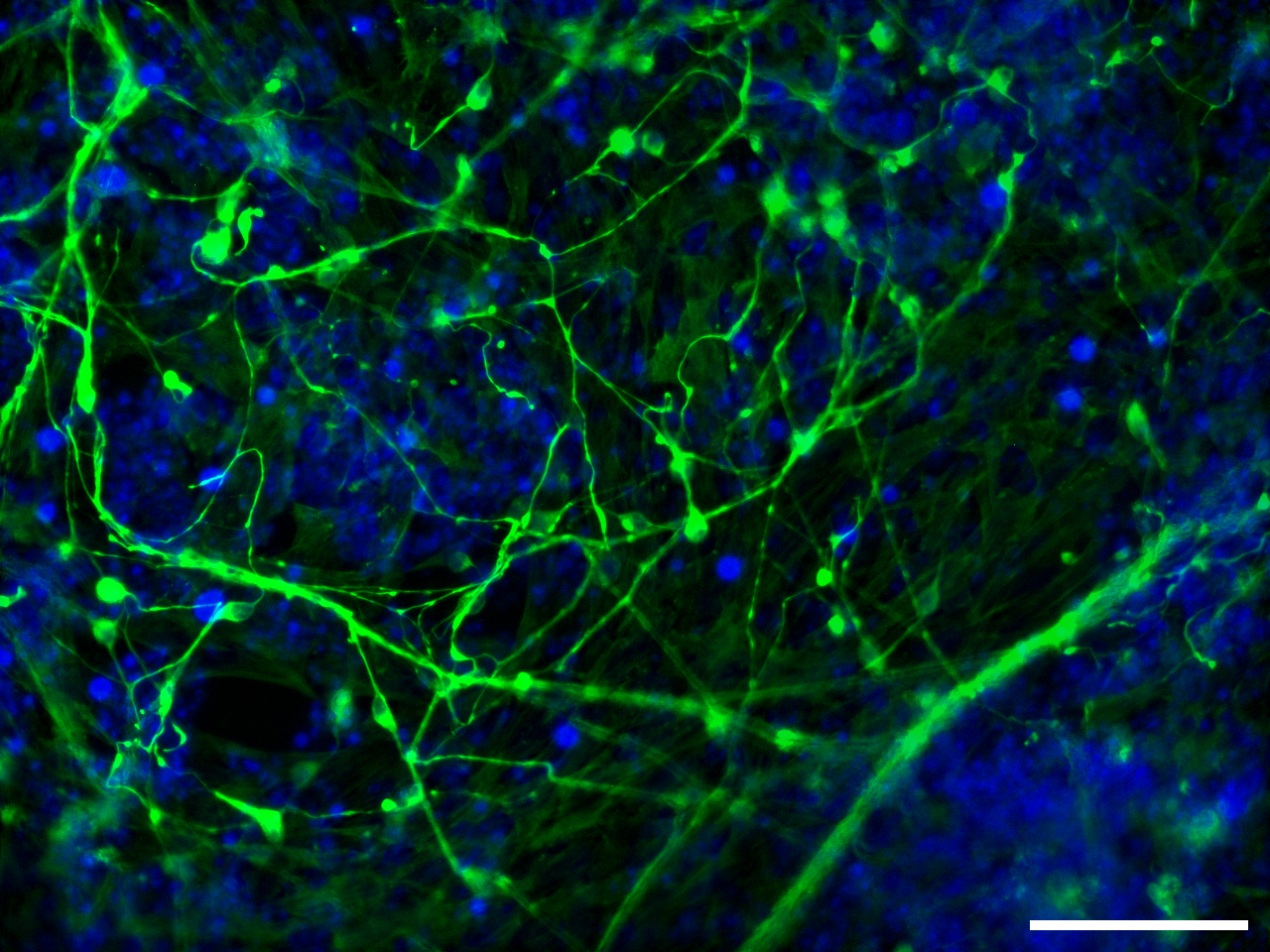

Supplement: Supplementary file 4 — Source data Fig. 1 [file 44318_2024_299_MOESM4_ESM.zip › Figure 1/1M/shCtrl_PRPH.tif]

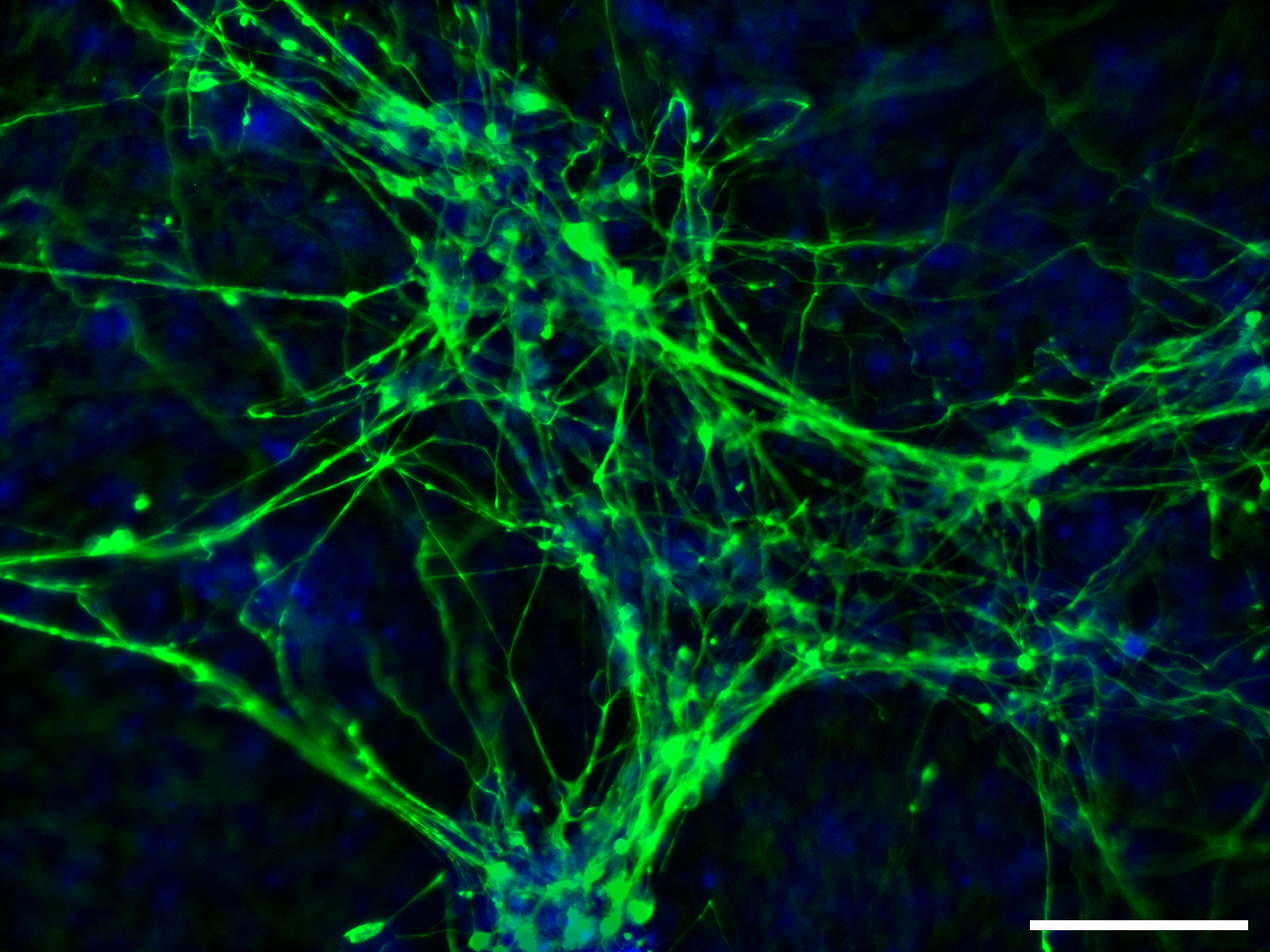

Supplement: Supplementary file 4 — Source data Fig. 1 [file 44318_2024_299_MOESM4_ESM.zip › Figure 1/1M/shMETTL3-1_PRPH.tif]

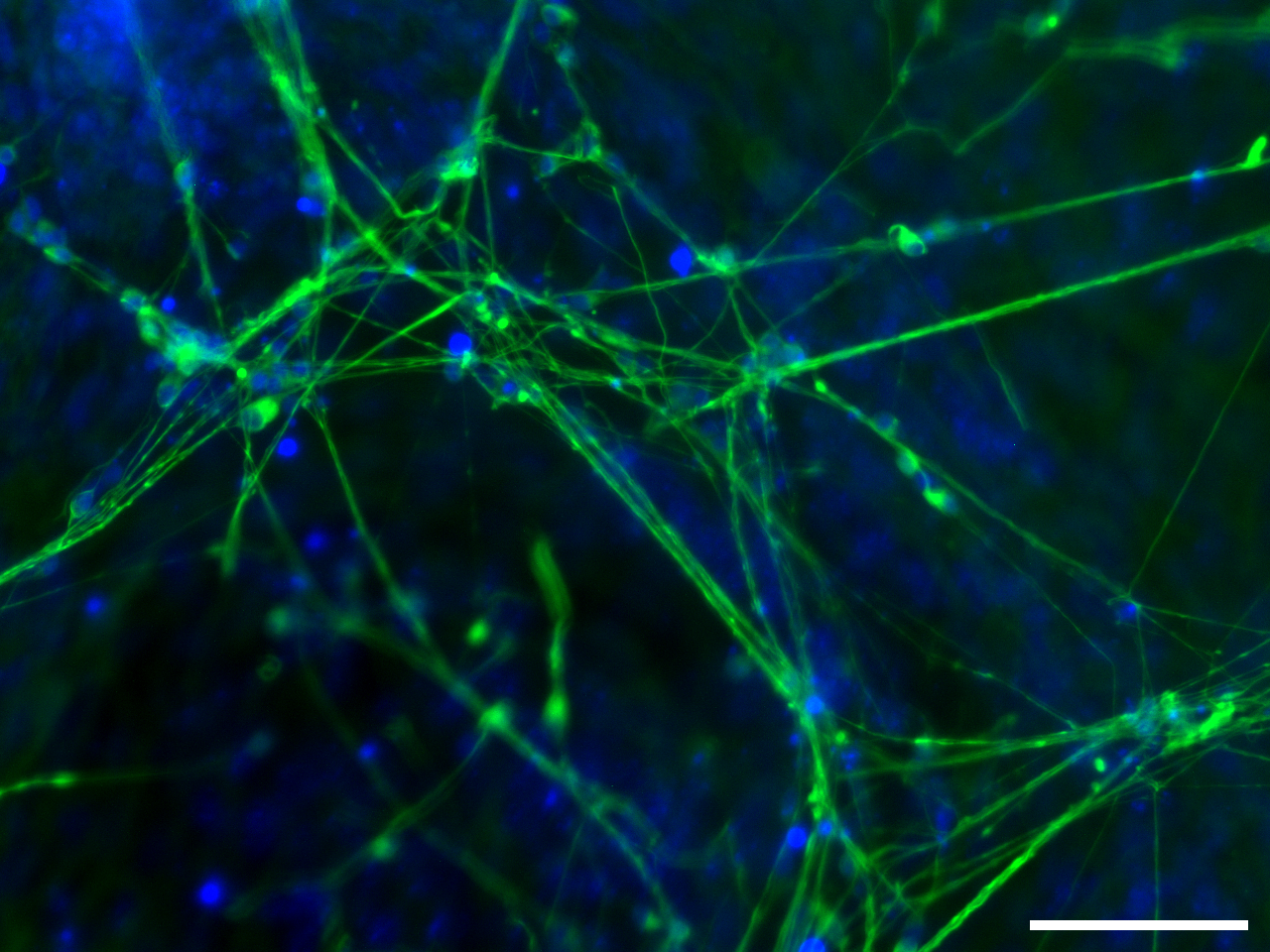

Supplement: Supplementary file 4 — Source data Fig. 1 [file 44318_2024_299_MOESM4_ESM.zip › Figure 1/1M/shMETTL3-2_PRPH.tif]

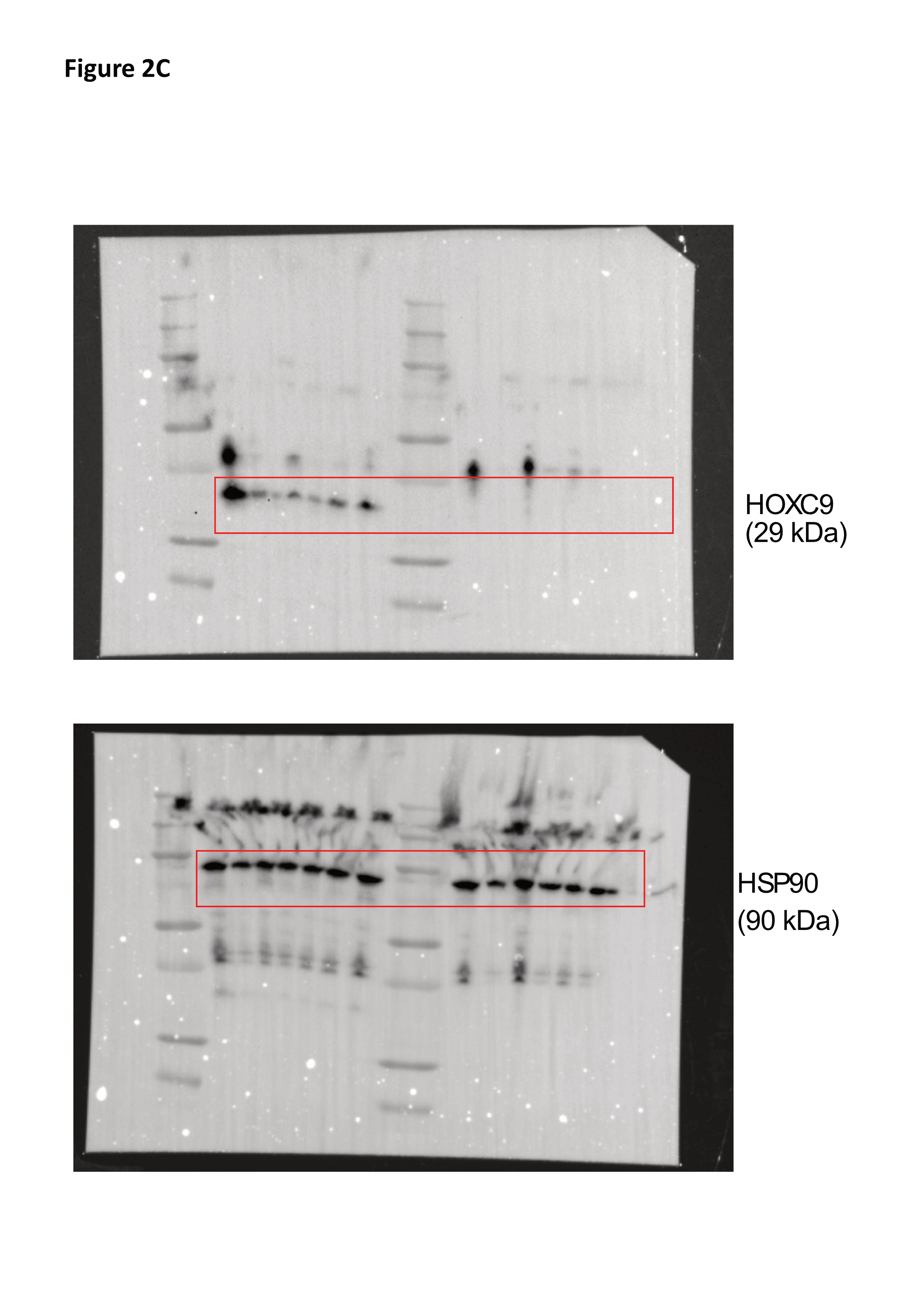

Supplement: Supplementary file 5 — Source data Fig. 2 [file 44318_2024_299_MOESM5_ESM.zip › Figure 2/2C/Source data for Figure 2C.tiff]

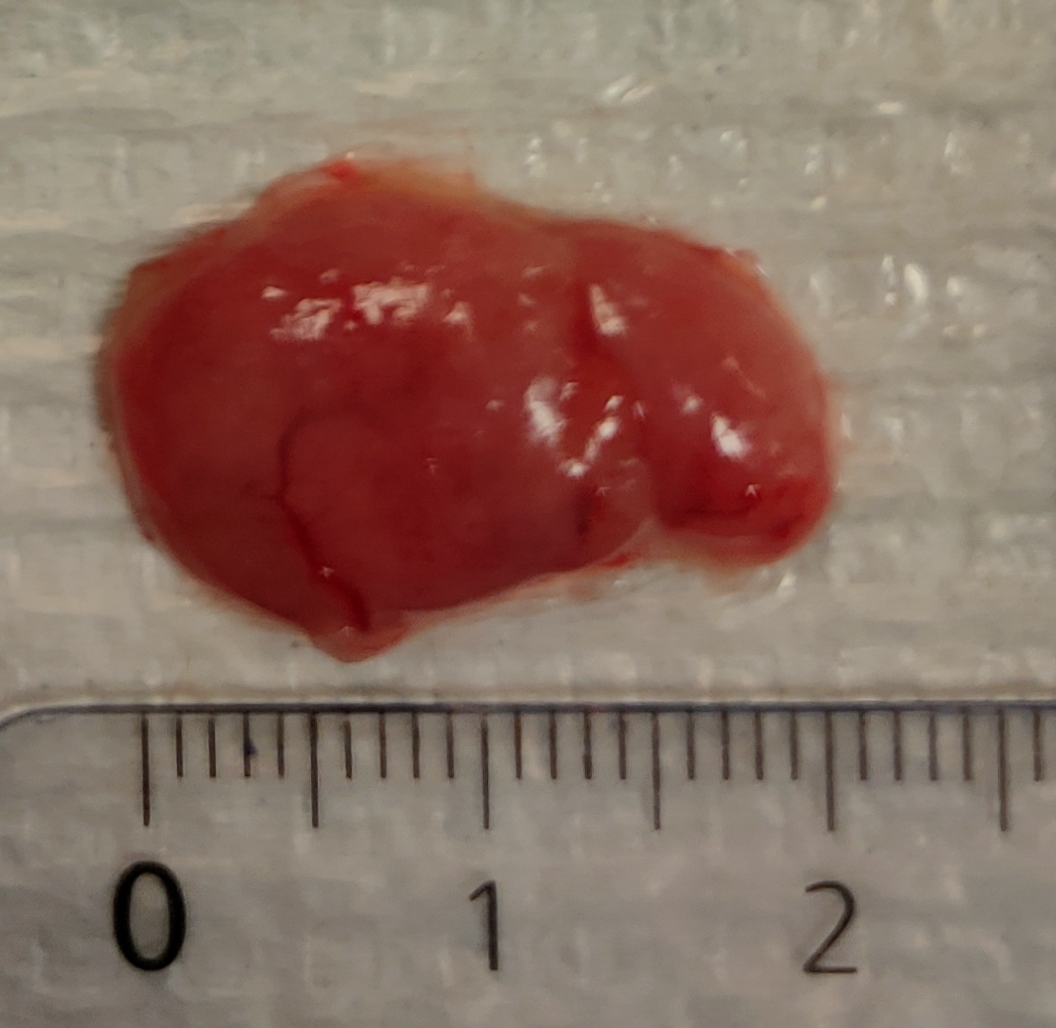

Supplement: Supplementary file 5 — Source data Fig. 2 [file 44318_2024_299_MOESM5_ESM.zip › Figure 2/2I/IMG_20230903_183756 BE2 CTRL.tif]

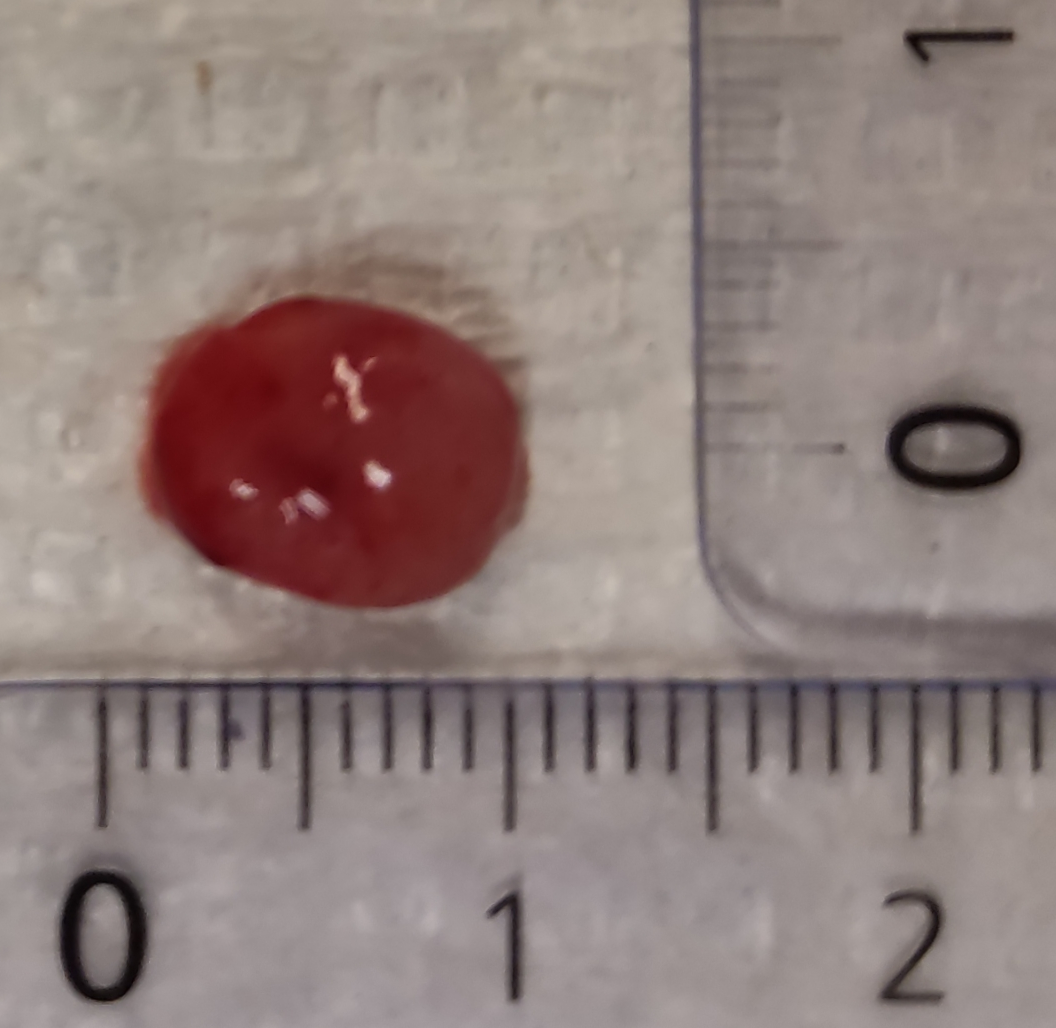

Supplement: Supplementary file 5 — Source data Fig. 2 [file 44318_2024_299_MOESM5_ESM.zip › Figure 2/2I/IMG_20230903_183930 BE2 M3 KD.tif]

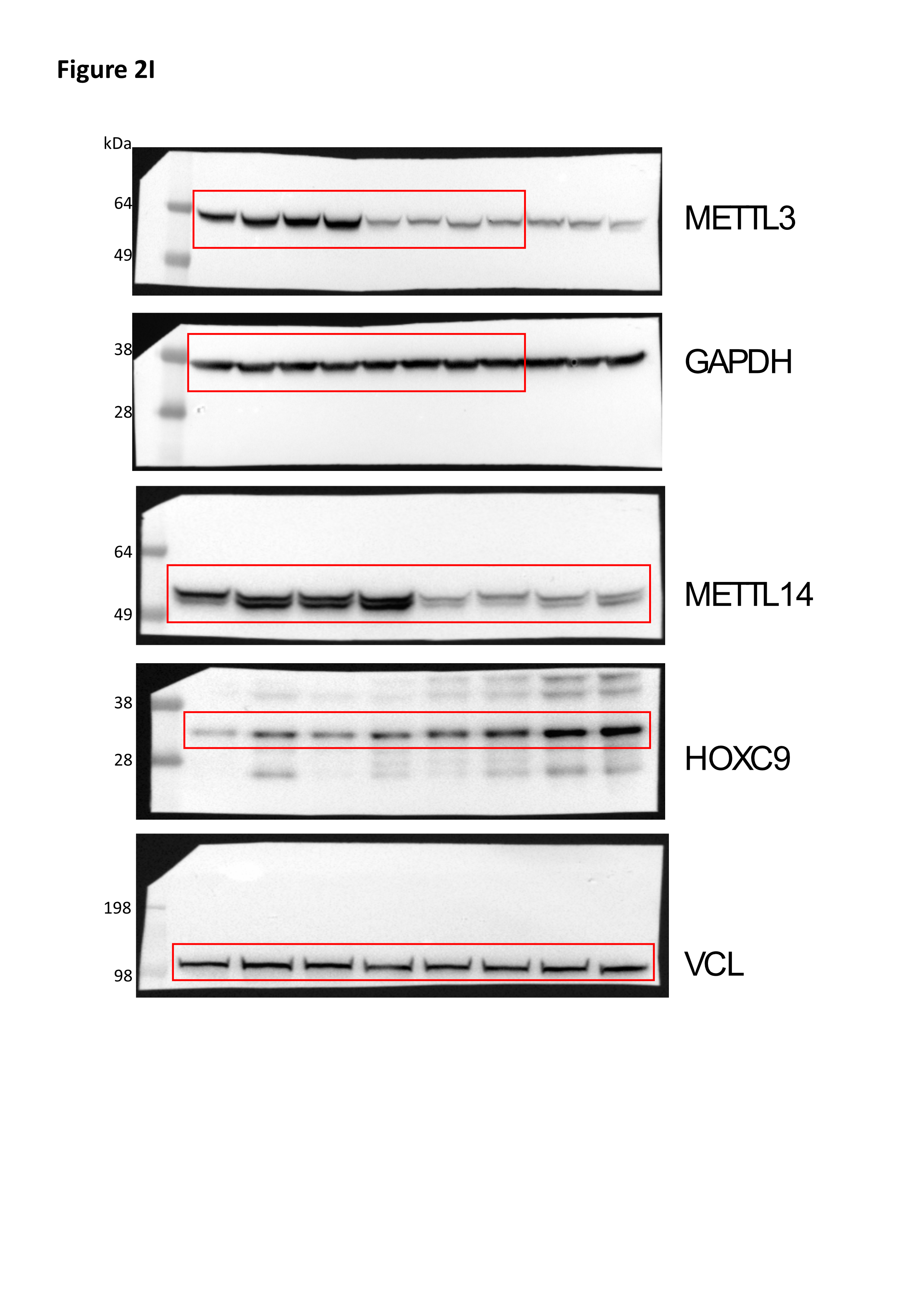

Supplement: Supplementary file 5 — Source data Fig. 2 [file 44318_2024_299_MOESM5_ESM.zip › Figure 2/2I/Source data for Figure 2I.tiff]

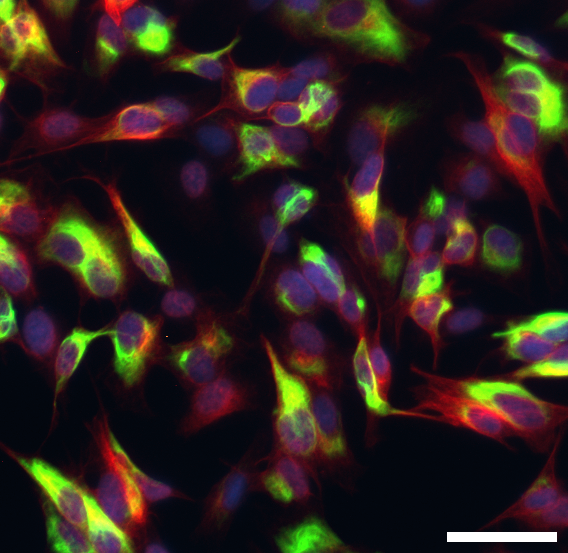

Supplement: Supplementary file 5 — Source data Fig. 2 [file 44318_2024_299_MOESM5_ESM.zip › Figure 2/2J/BE2_shCtrl_PRPH_g_TUUB3_r_merge.tif]

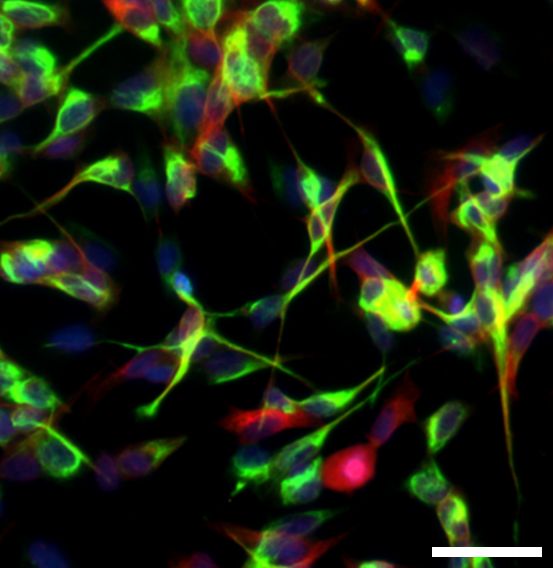

Supplement: Supplementary file 5 — Source data Fig. 2 [file 44318_2024_299_MOESM5_ESM.zip › Figure 2/2J/BE2_shMETTL3-1_PRPH_g_TUUB3_r_merge.tif]

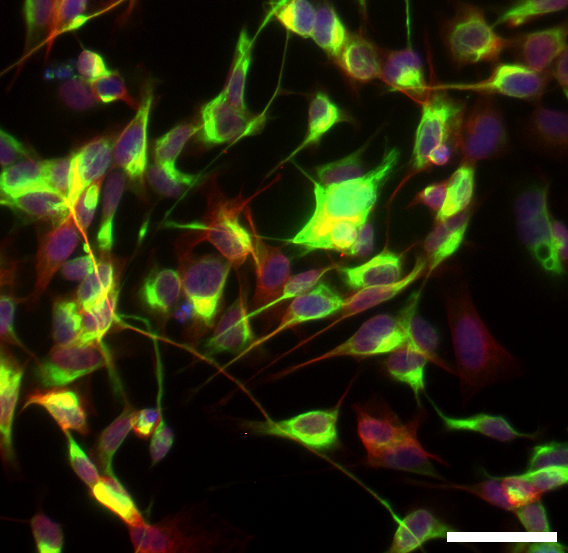

Supplement: Supplementary file 5 — Source data Fig. 2 [file 44318_2024_299_MOESM5_ESM.zip › Figure 2/2J/BE2_shMETTL3-2_PRPH_g_TUUB3_r_merge.tif]

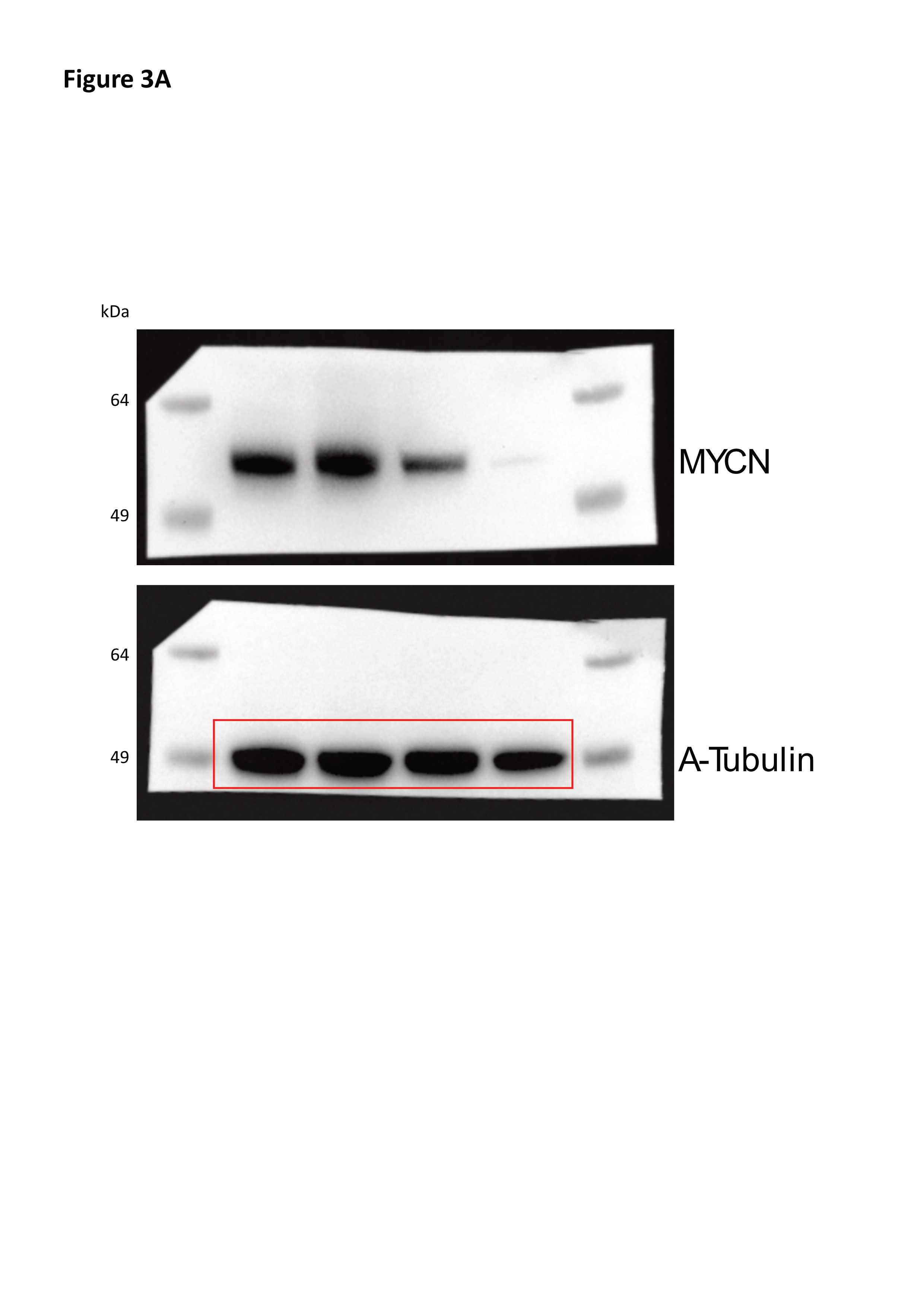

Supplement: Supplementary file 6 — Source data Fig. 3 [file 44318_2024_299_MOESM6_ESM.zip › Figure 3/3A/Source data for Figure 3A.tiff]

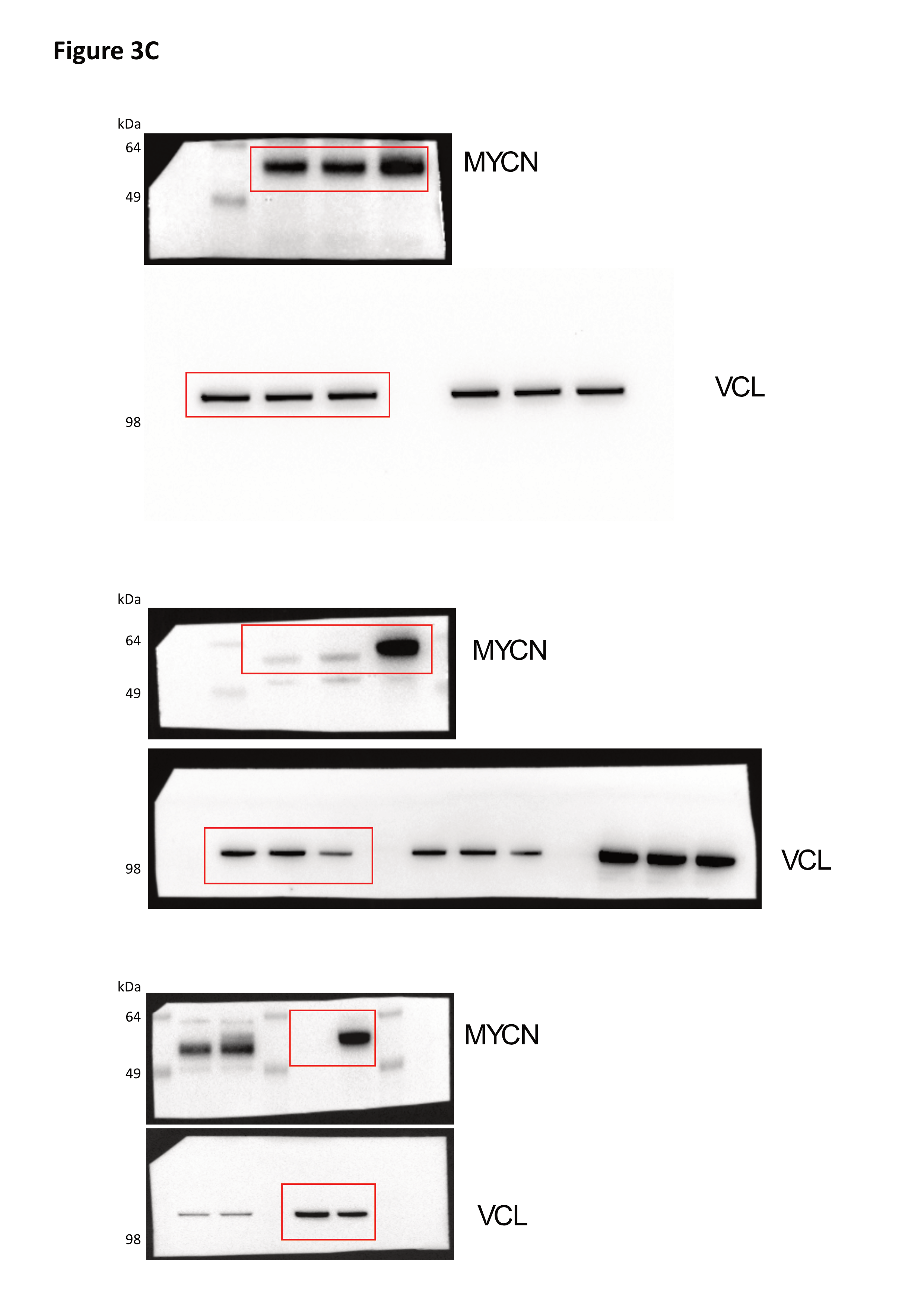

Supplement: Supplementary file 6 — Source data Fig. 3 [file 44318_2024_299_MOESM6_ESM.zip › Figure 3/3C/Source data for Figure 3C.tiff]

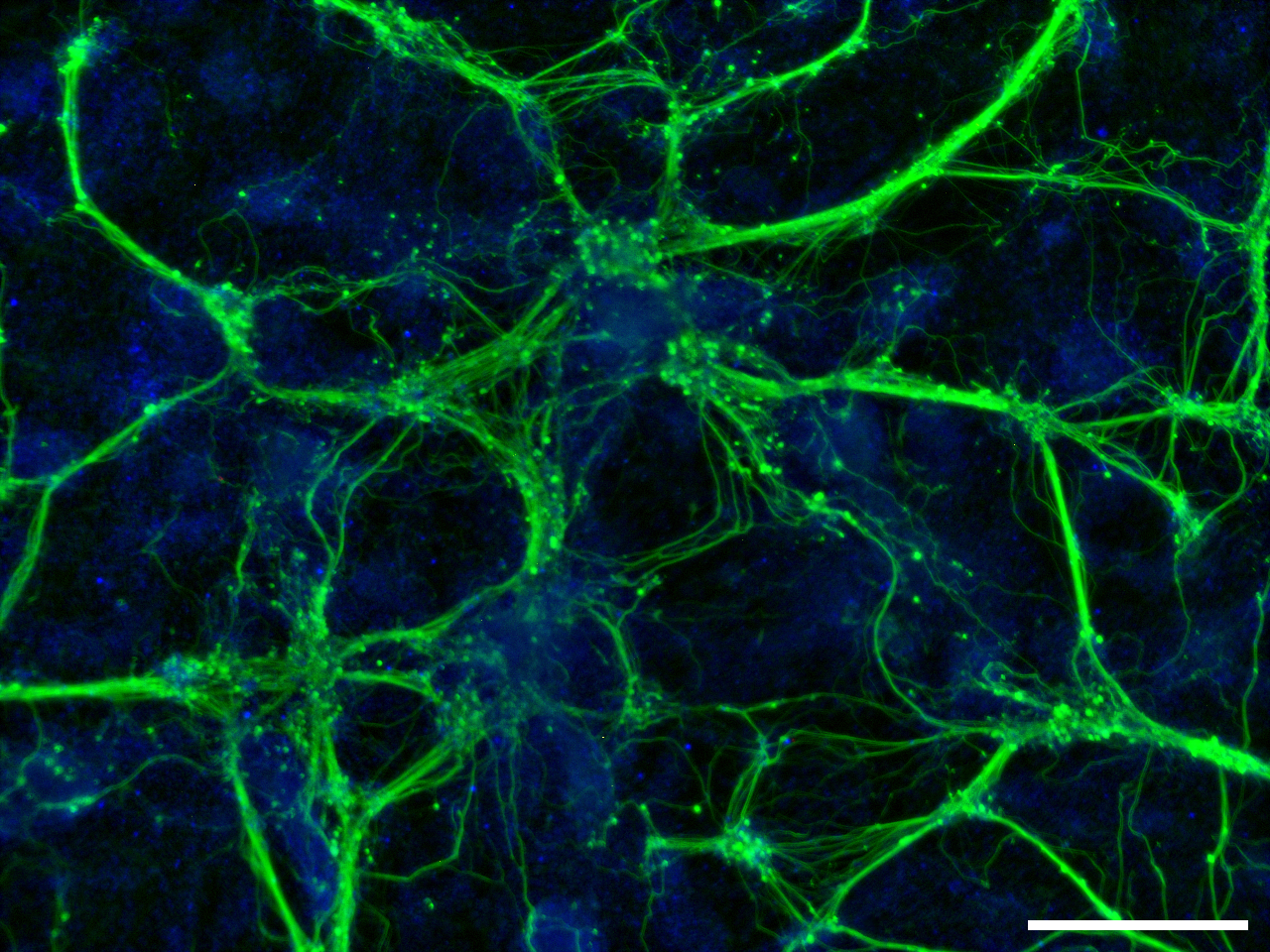

Supplement: Supplementary file 6 — Source data Fig. 3 [file 44318_2024_299_MOESM6_ESM.zip › Figure 3/3D/no_dox_SN_PRPH_g_MYCN_r_merge.tif]

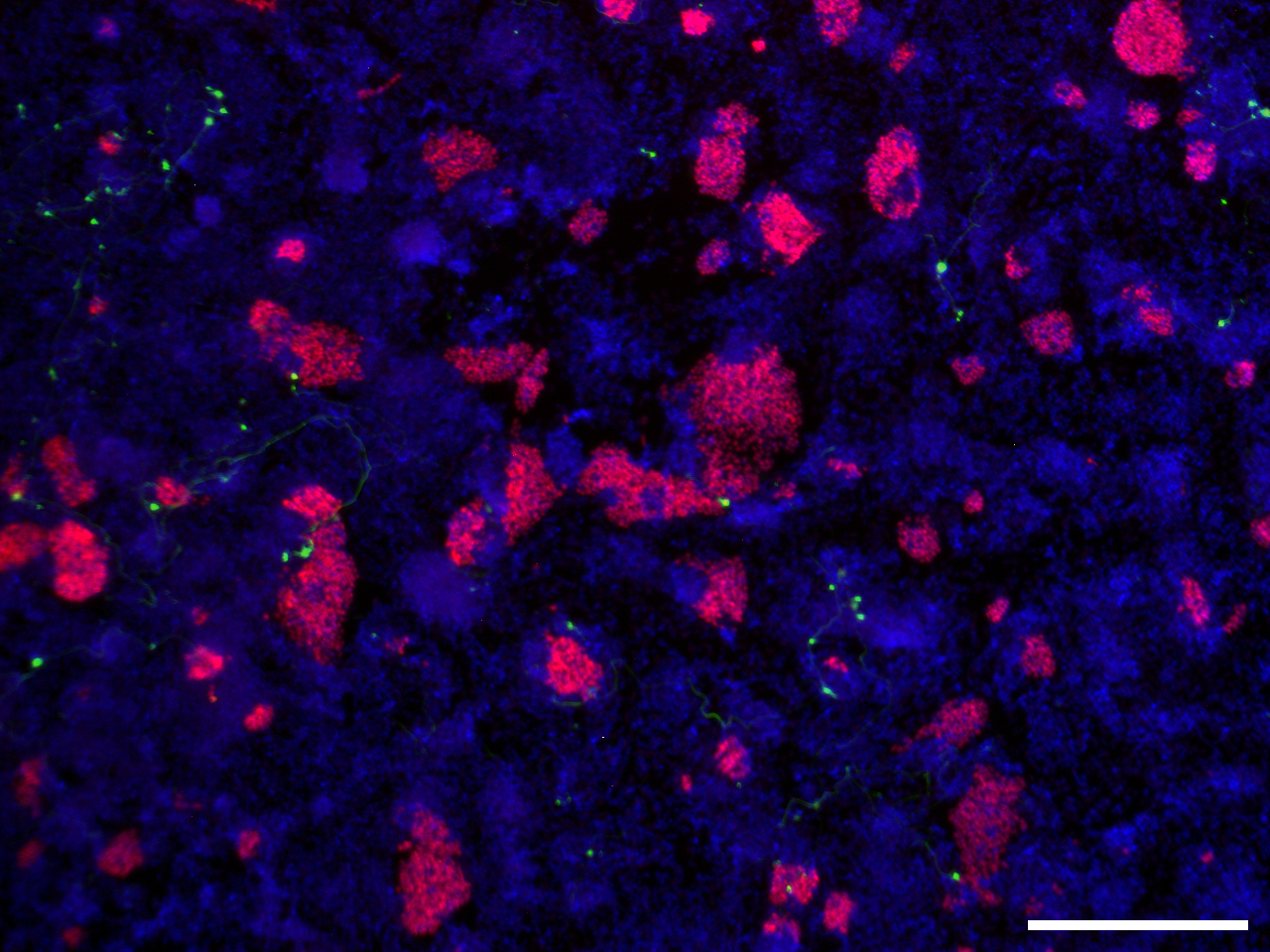

Supplement: Supplementary file 6 — Source data Fig. 3 [file 44318_2024_299_MOESM6_ESM.zip › Figure 3/3D/plus_dox_SN_PRPH_g_MYCN_r_merge.tif]

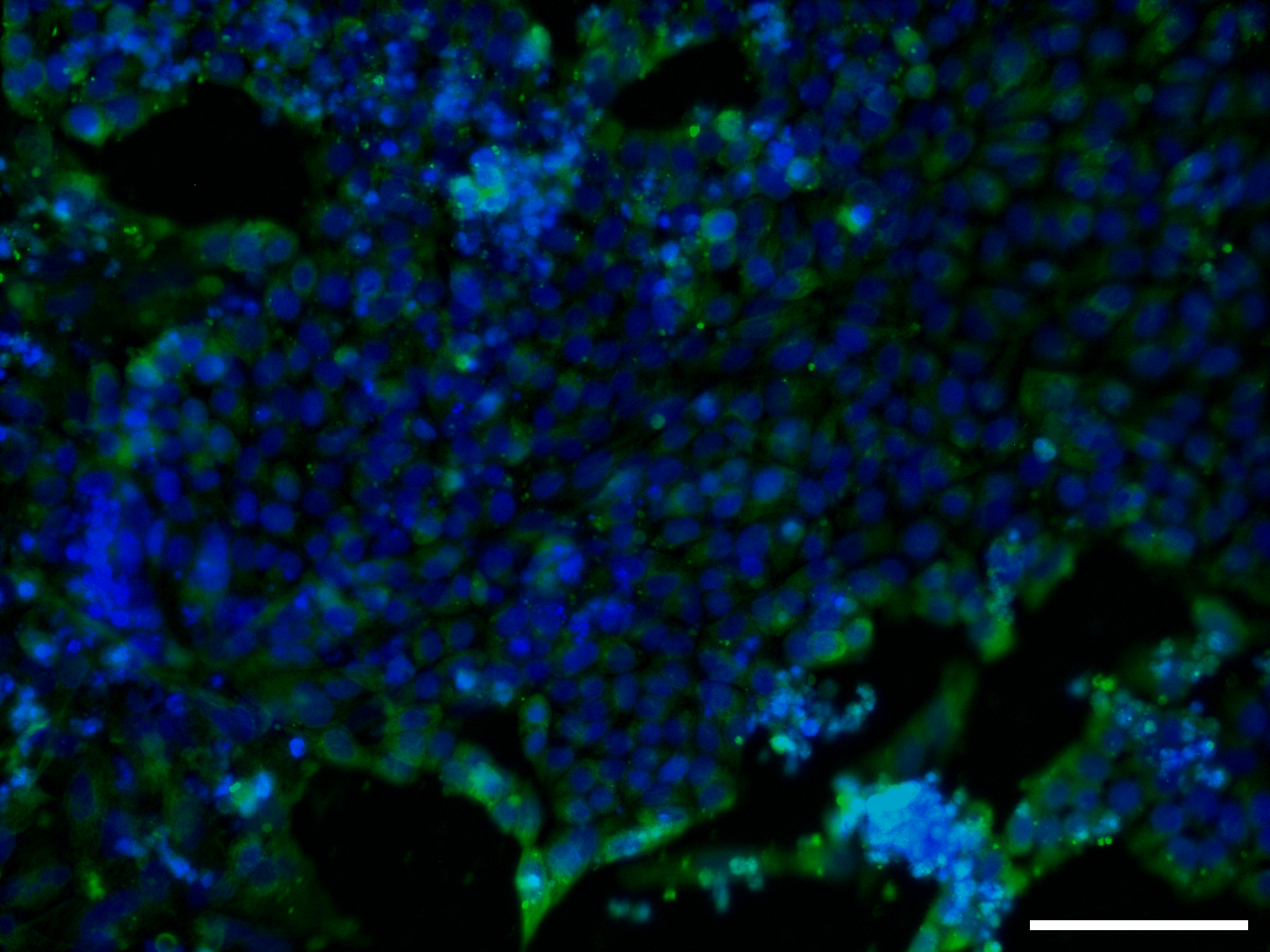

Supplement: Supplementary file 6 — Source data Fig. 3 [file 44318_2024_299_MOESM6_ESM.zip › Figure 3/3E/+dox_BetaIII_g_DAPI_merge.tif]

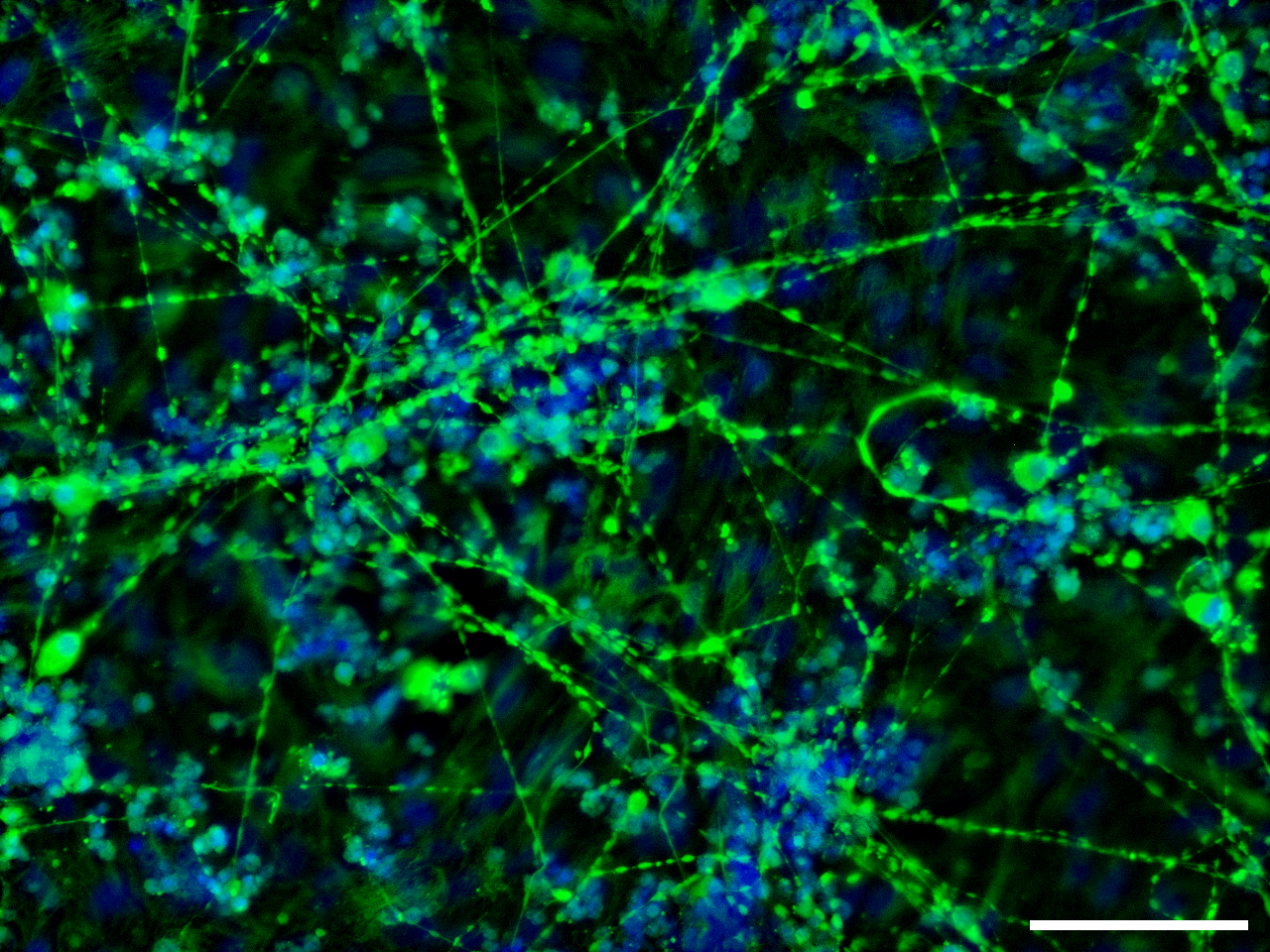

Supplement: Supplementary file 6 — Source data Fig. 3 [file 44318_2024_299_MOESM6_ESM.zip › Figure 3/3E/-dox_BetaIII_g_DAPI_merge.tif]

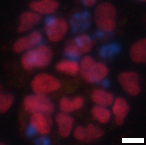

Supplement: Supplementary file 6 — Source data Fig. 3 [file 44318_2024_299_MOESM6_ESM.zip › Figure 3/3F/no dox_Flag_g_hoxc8_r_merge.tif]

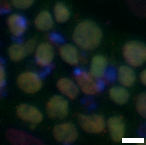

Supplement: Supplementary file 6 — Source data Fig. 3 [file 44318_2024_299_MOESM6_ESM.zip › Figure 3/3F/plus dox_HOXC8_r_Flag_g_merge.tif]

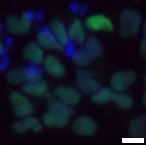

Supplement: Supplementary file 6 — Source data Fig. 3 [file 44318_2024_299_MOESM6_ESM.zip › Figure 3/3G/no dox_HOXC9_g_Flag_r_merge.tif]

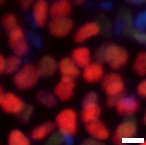

Supplement: Supplementary file 6 — Source data Fig. 3 [file 44318_2024_299_MOESM6_ESM.zip › Figure 3/3G/plus dox_HOXC9_g_Flag_r_merge.tif]

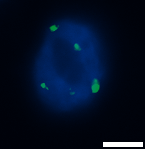

Supplement: Supplementary file 7 — Source data Fig. 4 [file 44318_2024_299_MOESM7_ESM.zip › Figure 4/4A/PLA_METTL3_MYCN_SHEP_dox plus_MYCN.tif]

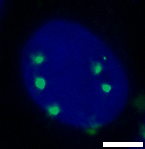

Supplement: Supplementary file 7 — Source data Fig. 4 [file 44318_2024_299_MOESM7_ESM.zip › Figure 4/4A/PLA_METTL3_MYCN_tNCC.tif]

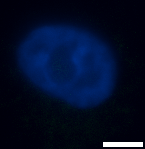

Supplement: Supplementary file 7 — Source data Fig. 4 [file 44318_2024_299_MOESM7_ESM.zip › Figure 4/4A/PLA_Neg_control_only MYCN_SHEP_dox plus_MYCN.tif]

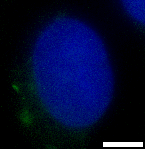

Supplement: Supplementary file 7 — Source data Fig. 4 [file 44318_2024_299_MOESM7_ESM.zip › Figure 4/4A/PLA_Neg_control_only MYCN_tNCC.tif]

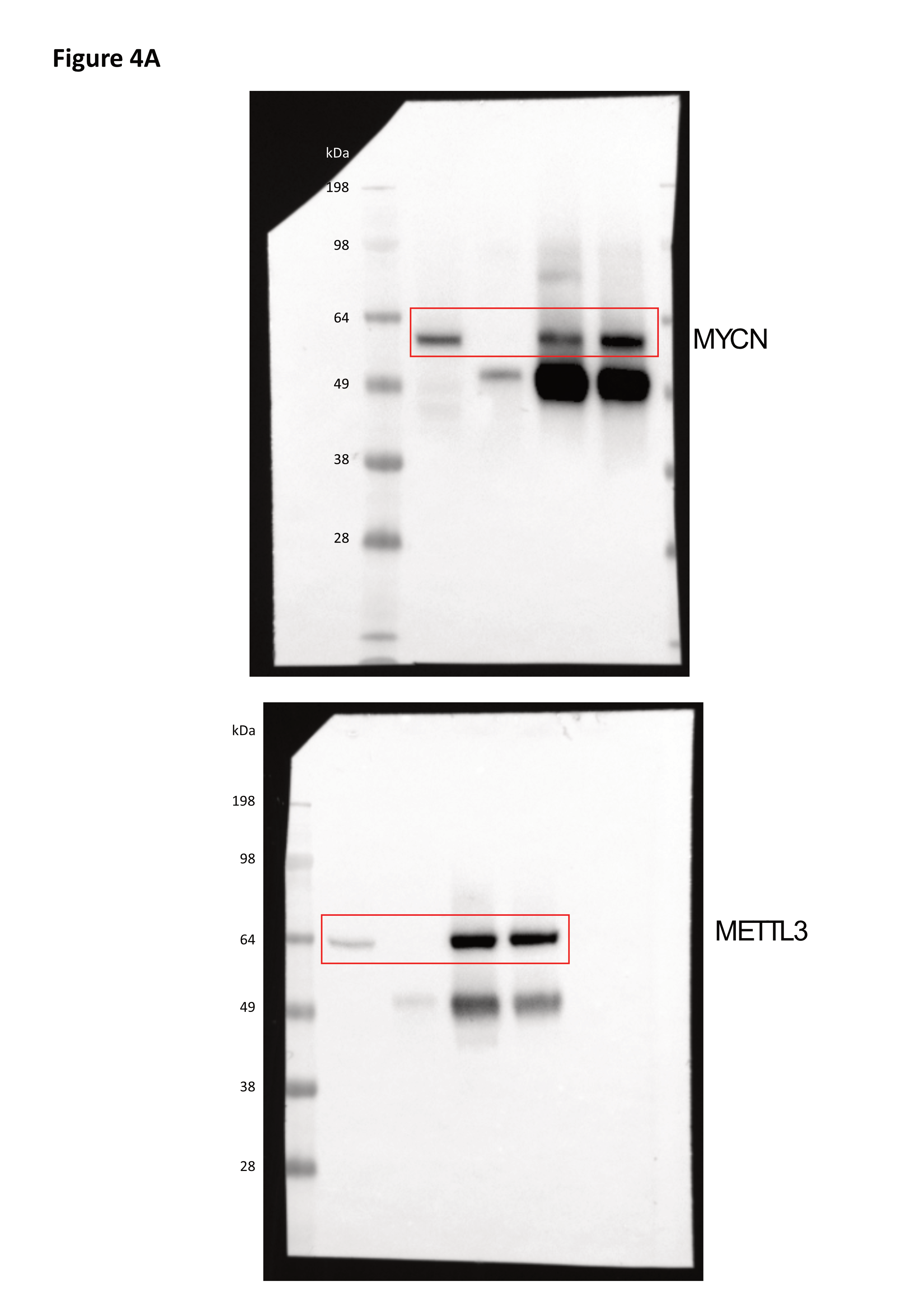

Supplement: Supplementary file 7 — Source data Fig. 4 [file 44318_2024_299_MOESM7_ESM.zip › Figure 4/4A/Source data for Figure 4A.tiff]

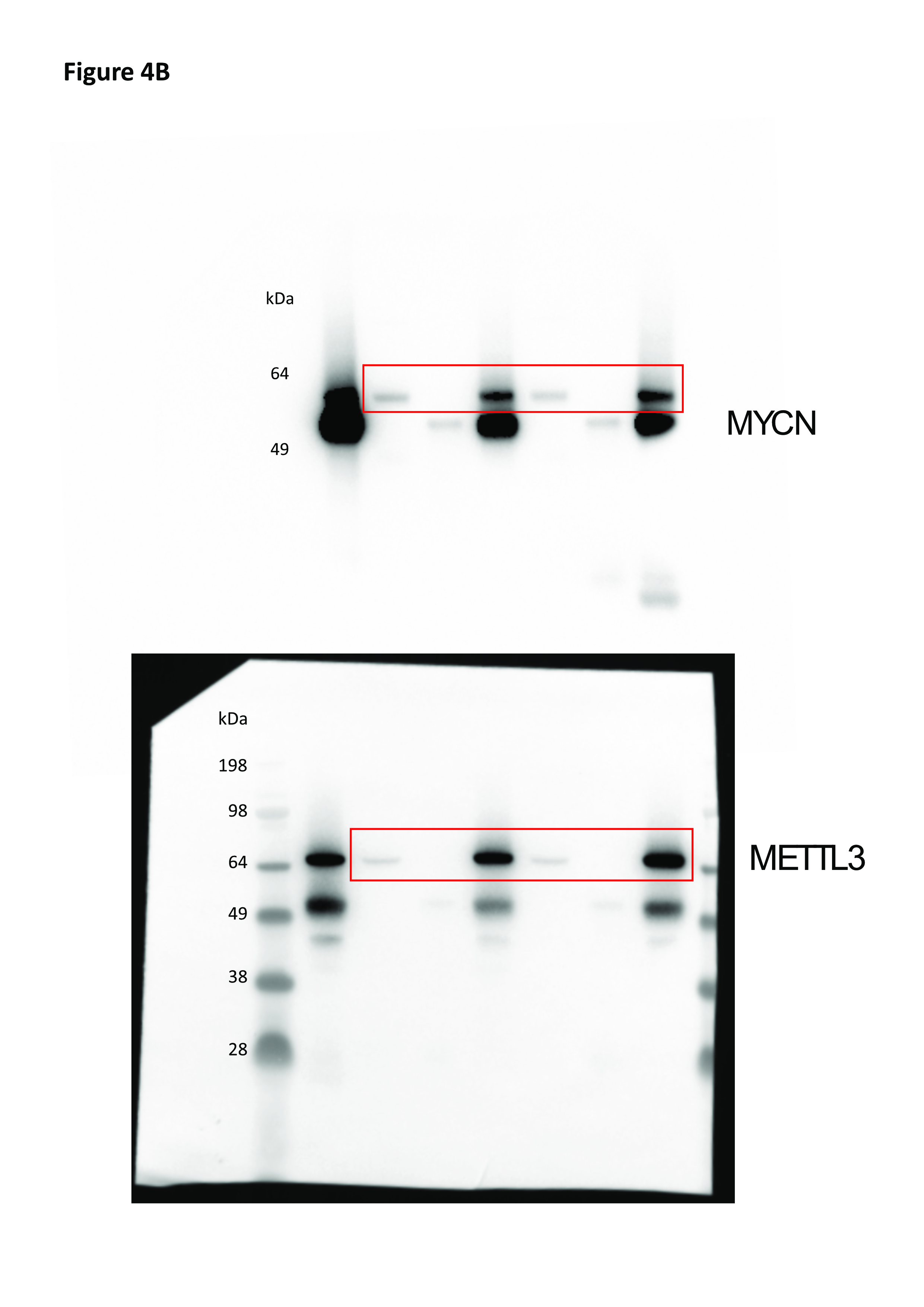

Supplement: Supplementary file 7 — Source data Fig. 4 [file 44318_2024_299_MOESM7_ESM.zip › Figure 4/4B/Source data for Figure 4B.tiff]

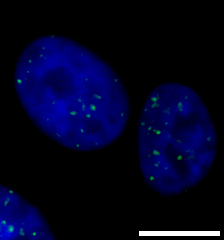

Supplement: Supplementary file 7 — Source data Fig. 4 [file 44318_2024_299_MOESM7_ESM.zip › Figure 4/4O/PLA_METTL14-H3K36me3_minus Dox.tif]

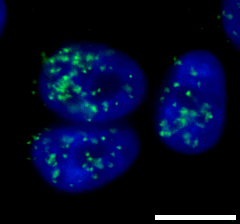

Supplement: Supplementary file 7 — Source data Fig. 4 [file 44318_2024_299_MOESM7_ESM.zip › Figure 4/4O/PLA_METTL14-H3K36me3_plus Dox.tif]

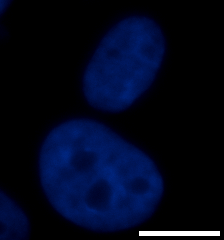

Supplement: Supplementary file 7 — Source data Fig. 4 [file 44318_2024_299_MOESM7_ESM.zip › Figure 4/4O/PLA_Neg_control_only H3K36me3.tif]

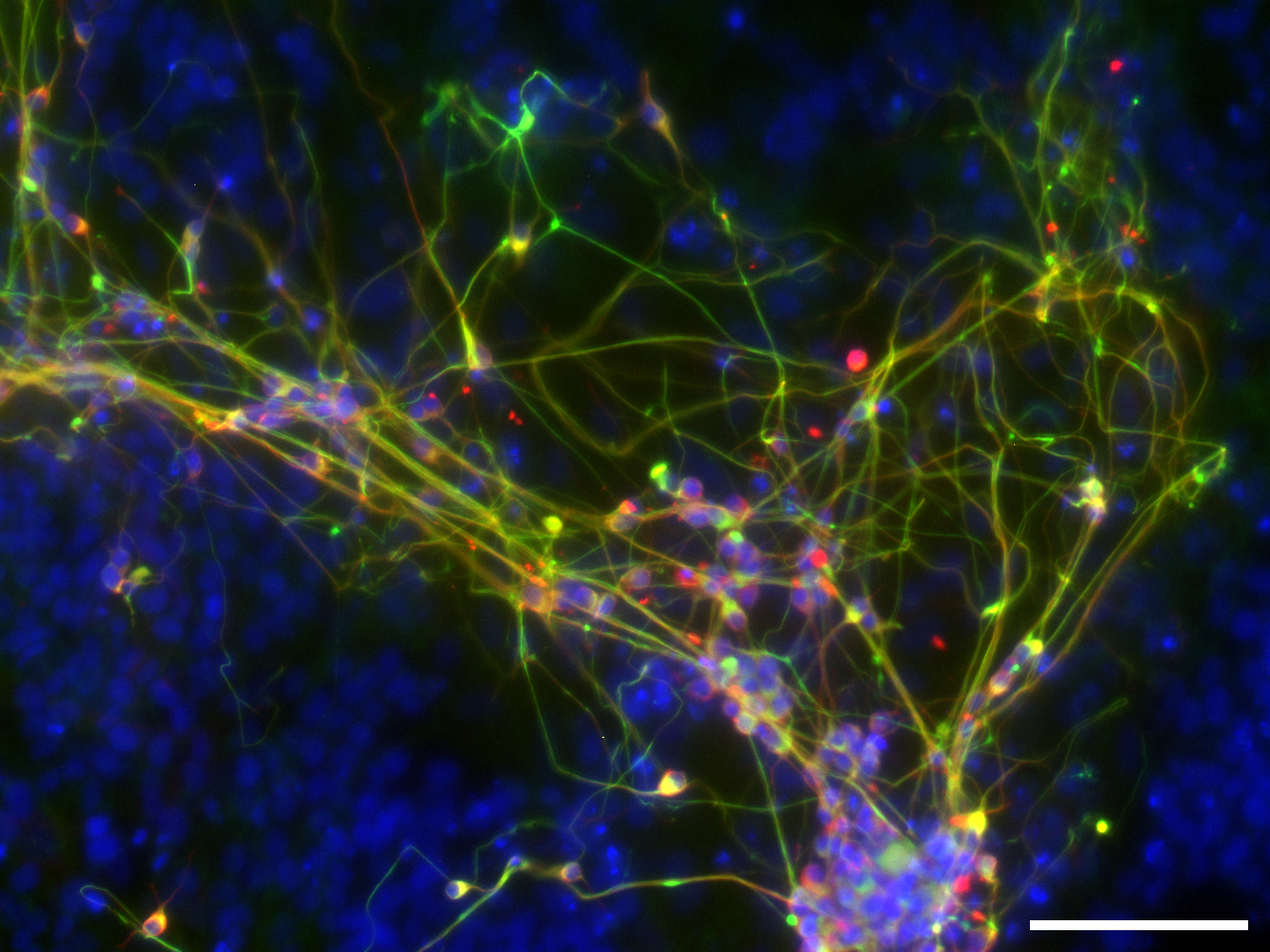

Supplement: Supplementary file 8 — Source data Fig. 5 [file 44318_2024_299_MOESM8_ESM.zip › Figure 5/5B/SN_HOXC9 OE LV_PRPH_g_Beta tub_r_merge.tif]

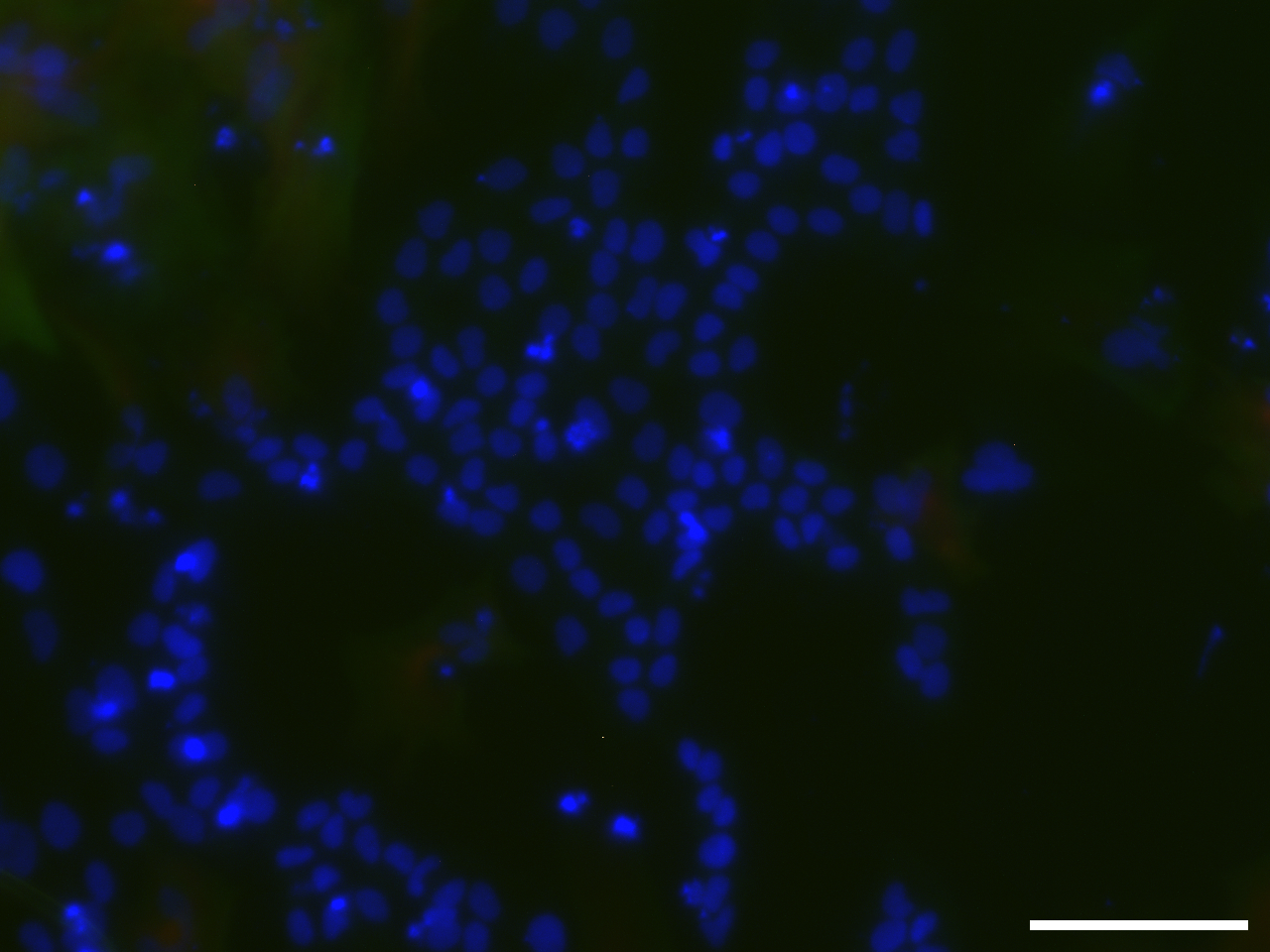

Supplement: Supplementary file 8 — Source data Fig. 5 [file 44318_2024_299_MOESM8_ESM.zip › Figure 5/5B/SN_Mock LV_PRPH_g_Beta tub_r_merge.tif]

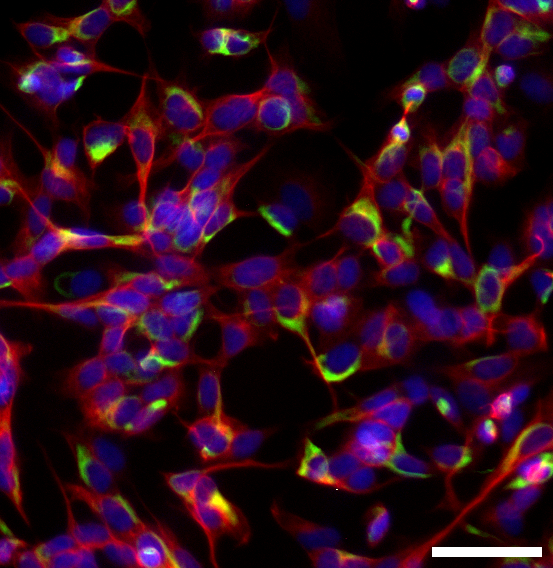

Supplement: Supplementary file 8 — Source data Fig. 5 [file 44318_2024_299_MOESM8_ESM.zip › Figure 5/5C/HOXC9_gRNA1_mutFTO_RA_PRPH_g_TUBB3_r_merge.tif]

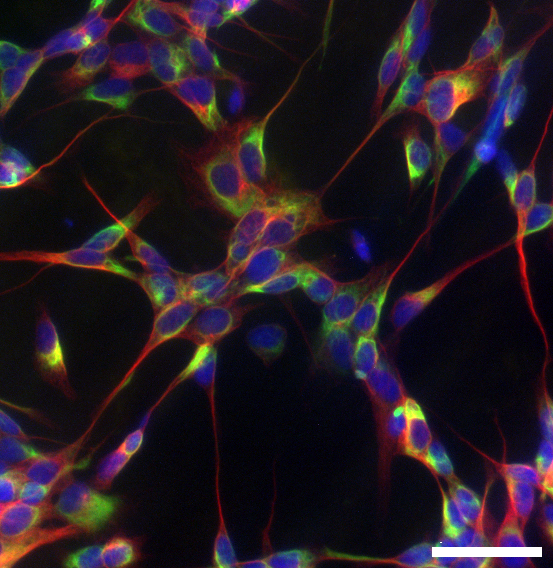

Supplement: Supplementary file 8 — Source data Fig. 5 [file 44318_2024_299_MOESM8_ESM.zip › Figure 5/5C/HOXC9_gRNA1_wtFTO_RA_PRPH_g_TUBB3_r_merge.tif]

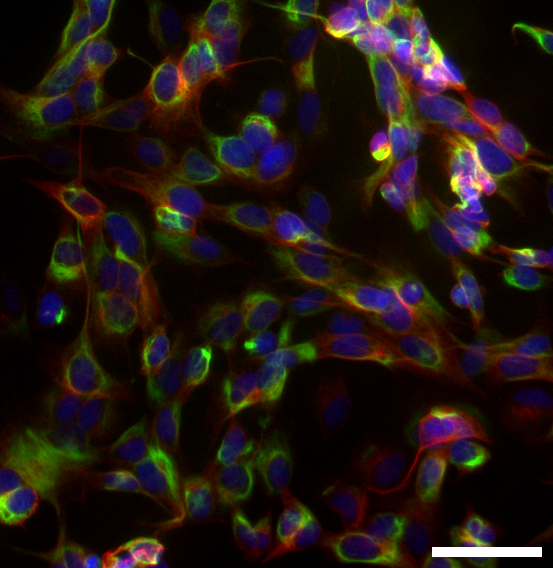

Supplement: Supplementary file 8 — Source data Fig. 5 [file 44318_2024_299_MOESM8_ESM.zip › Figure 5/5C/HOXC9_gRNA2_mutFTO_RA_PRPH_g_TUBB3_r_merge.tif]

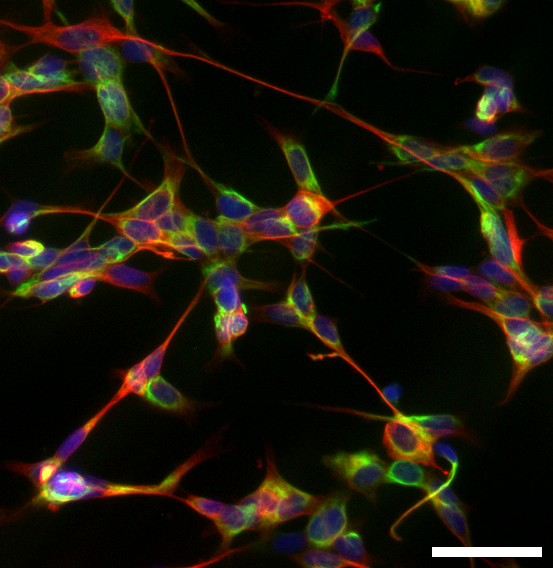

Supplement: Supplementary file 8 — Source data Fig. 5 [file 44318_2024_299_MOESM8_ESM.zip › Figure 5/5C/HOXC9_gRNA2_wtFTO_RA_PRPH_g_TUBB3_r_merge.tif]

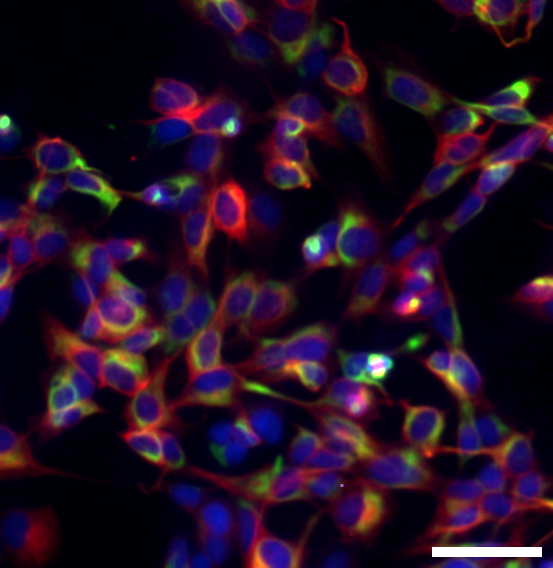

Supplement: Supplementary file 8 — Source data Fig. 5 [file 44318_2024_299_MOESM8_ESM.zip › Figure 5/5C/NTC_gRNA_mutFTO_RA_PRPH_g_TUBB3_r_merge.tif]

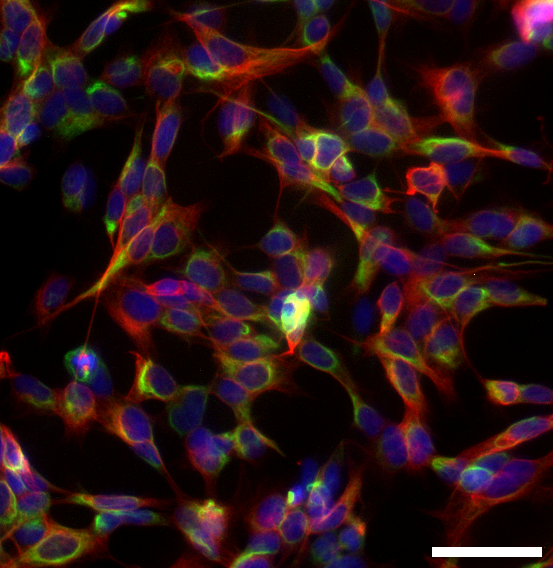

Supplement: Supplementary file 8 — Source data Fig. 5 [file 44318_2024_299_MOESM8_ESM.zip › Figure 5/5C/NTC_gRNA_wtFTO_RA_PRPH_g_TUBB3_r_merge.tif]

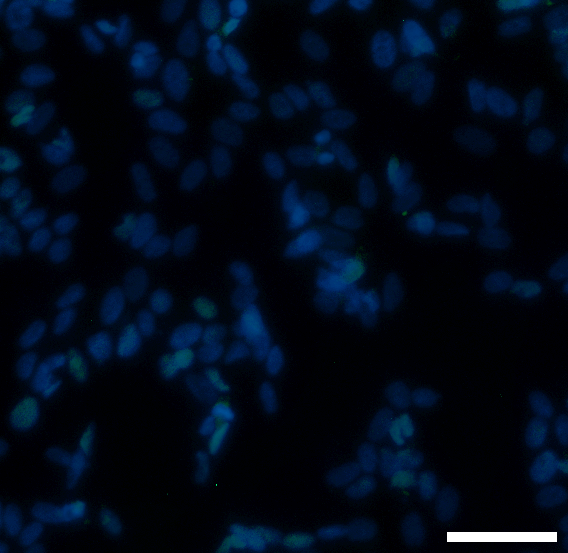

Supplement: Supplementary file 8 — Source data Fig. 5 [file 44318_2024_299_MOESM8_ESM.zip › Figure 5/5D/gRNA1_mutFTO_HOXC9_g.tif]

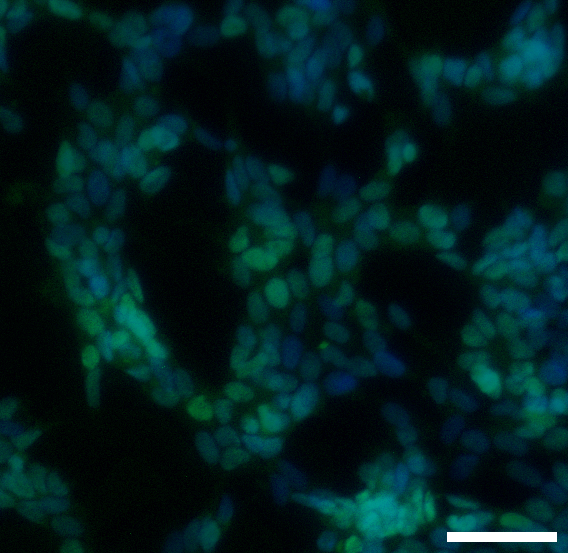

Supplement: Supplementary file 8 — Source data Fig. 5 [file 44318_2024_299_MOESM8_ESM.zip › Figure 5/5D/gRNA1_wtFTO_HOXC9_g.tif]

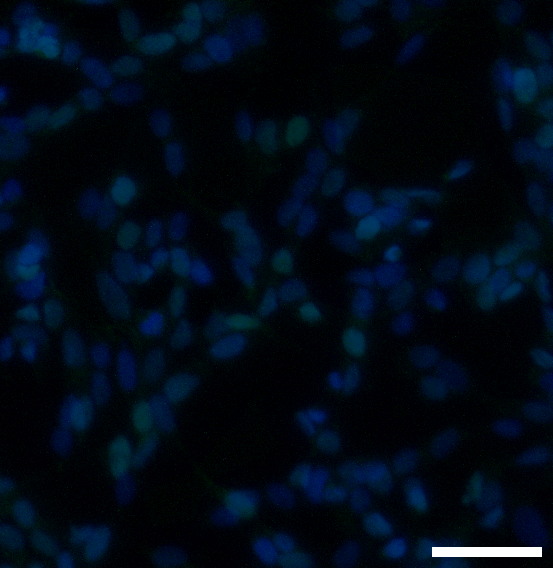

Supplement: Supplementary file 8 — Source data Fig. 5 [file 44318_2024_299_MOESM8_ESM.zip › Figure 5/5D/gRNA2_mutFTO_HOXC9_g.tif]

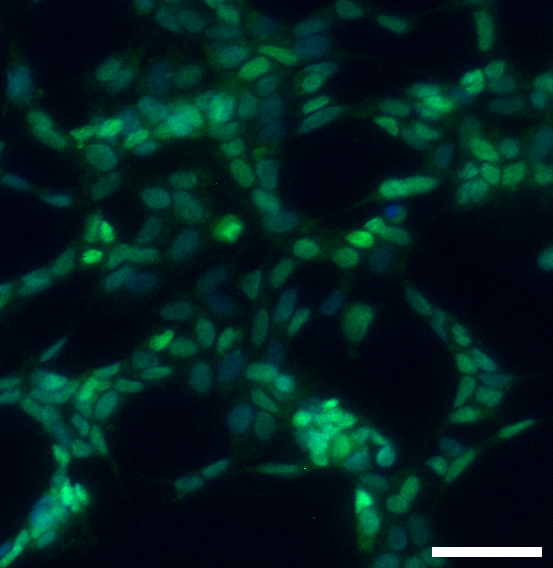

Supplement: Supplementary file 8 — Source data Fig. 5 [file 44318_2024_299_MOESM8_ESM.zip › Figure 5/5D/gRNA2_wtFTO_HOXC9_g.tif]

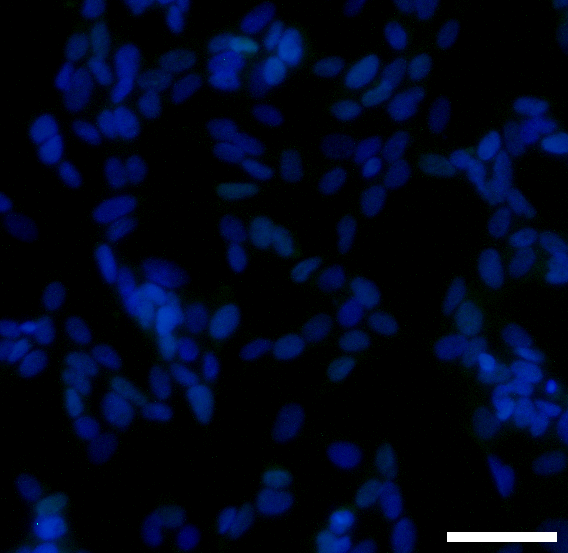

Supplement: Supplementary file 8 — Source data Fig. 5 [file 44318_2024_299_MOESM8_ESM.zip › Figure 5/5D/NTC_mutFTO_HOXC9_g.tif]

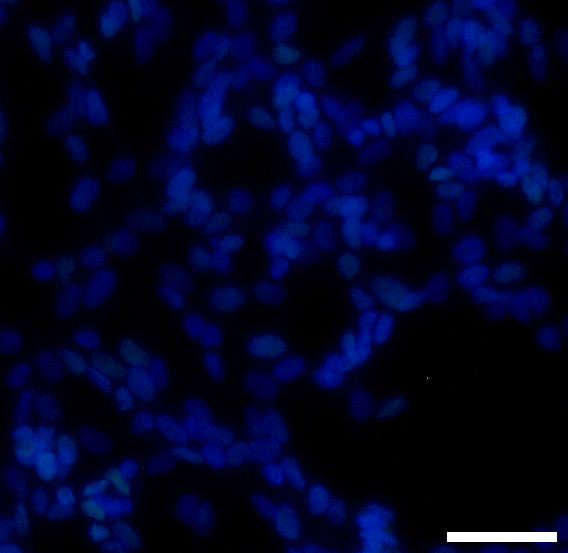

Supplement: Supplementary file 8 — Source data Fig. 5 [file 44318_2024_299_MOESM8_ESM.zip › Figure 5/5D/NTC_wtFTO_HOXC9_g.tif]

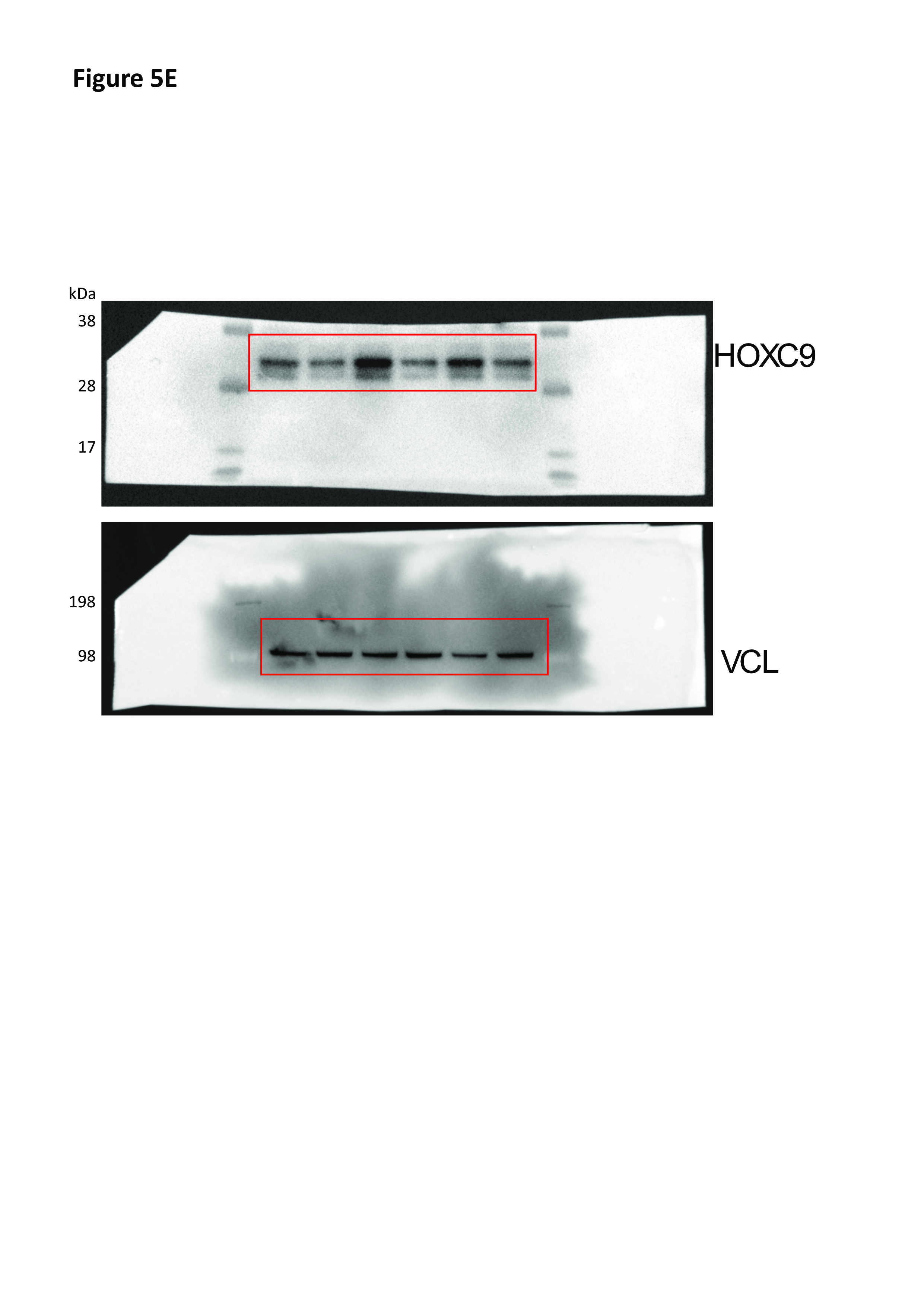

Supplement: Supplementary file 8 — Source data Fig. 5 [file 44318_2024_299_MOESM8_ESM.zip › Figure 5/5E/Source data for Figure 5E.tiff]

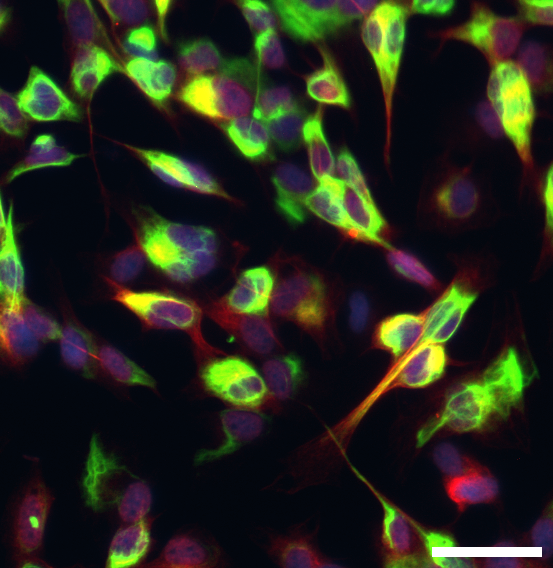

Supplement: Supplementary file 8 — Source data Fig. 5 [file 44318_2024_299_MOESM8_ESM.zip › Figure 5/5F/BE2_TetOshCtrl+CshPLKO_PRPH_g_TUBB3_r_merge.tif]

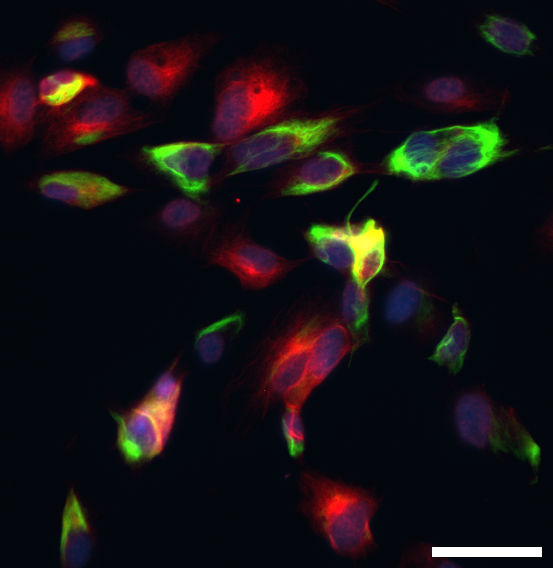

Supplement: Supplementary file 8 — Source data Fig. 5 [file 44318_2024_299_MOESM8_ESM.zip › Figure 5/5F/BE2_TetOshCtrl+shHOXC9_PRPH_g_TUBB3_r_merge.tif]

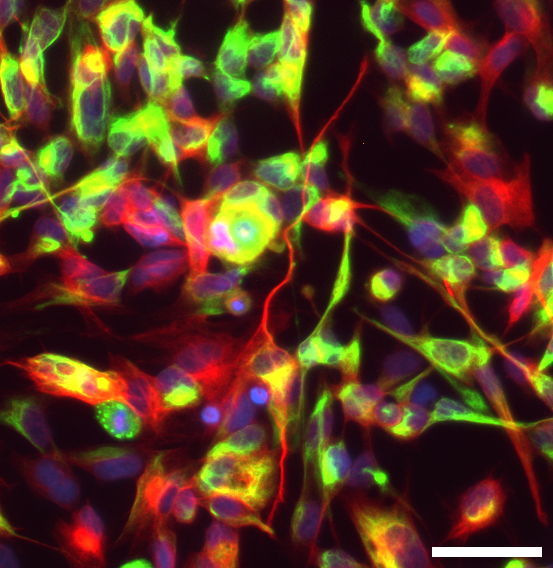

Supplement: Supplementary file 8 — Source data Fig. 5 [file 44318_2024_299_MOESM8_ESM.zip › Figure 5/5F/BE2_TetOshM3-1+CshPLKO_PRPH_g_TUBB3_r_merge.tif]

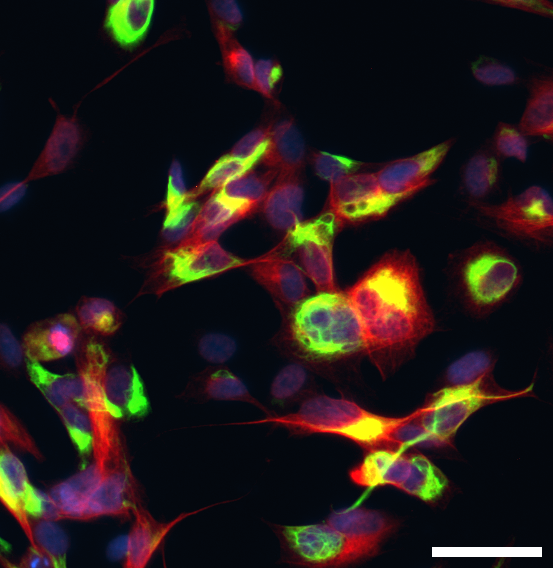

Supplement: Supplementary file 8 — Source data Fig. 5 [file 44318_2024_299_MOESM8_ESM.zip › Figure 5/5F/BE2_TetOshM3-1+shHOXC9_PRPH_g_TUBB3_r_merge.tif]

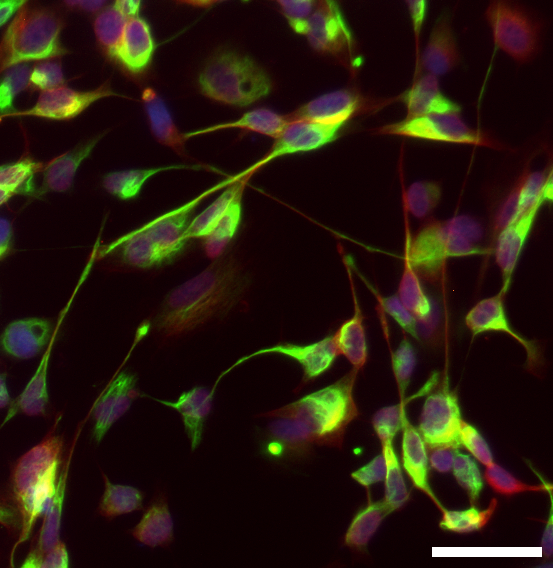

Supplement: Supplementary file 8 — Source data Fig. 5 [file 44318_2024_299_MOESM8_ESM.zip › Figure 5/5F/BE2_TetOshM3-2+CshPLKO_PRPH_g_TUBB3_r_merge.tif]

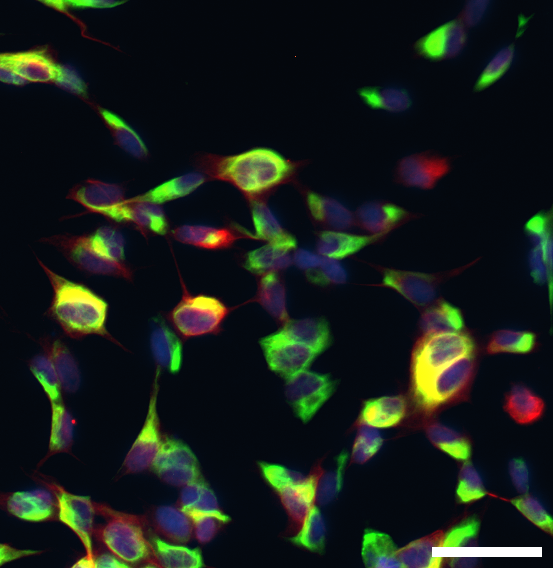

Supplement: Supplementary file 8 — Source data Fig. 5 [file 44318_2024_299_MOESM8_ESM.zip › Figure 5/5F/BE2_TetOshM3-2+shHOXC9_PRPH_g_TUBB3_r_merge.tif]

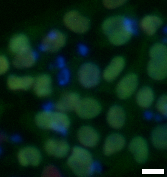

Supplement: Supplementary file 9 — Source data Fig. 6 [file 44318_2024_299_MOESM9_ESM.zip › Figure 6/6A/DMSO_with dox_Flag_g_hoxc8_r_merge.tif]

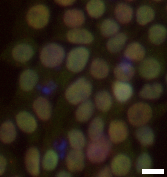

Supplement: Supplementary file 9 — Source data Fig. 6 [file 44318_2024_299_MOESM9_ESM.zip › Figure 6/6A/STM_with dox_Flag_g_hoxc8_r_merge.tif]

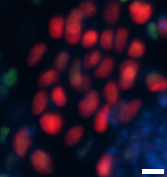

Supplement: Supplementary file 9 — Source data Fig. 6 [file 44318_2024_299_MOESM9_ESM.zip › Figure 6/6B/DMSO_with dox_Hoxc9_g_MYCN_r_merge.tif]

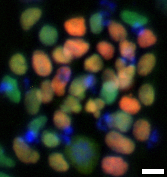

Supplement: Supplementary file 9 — Source data Fig. 6 [file 44318_2024_299_MOESM9_ESM.zip › Figure 6/6B/STM_with dox_Hoxc9_g_MYCN_r_merge.tif]

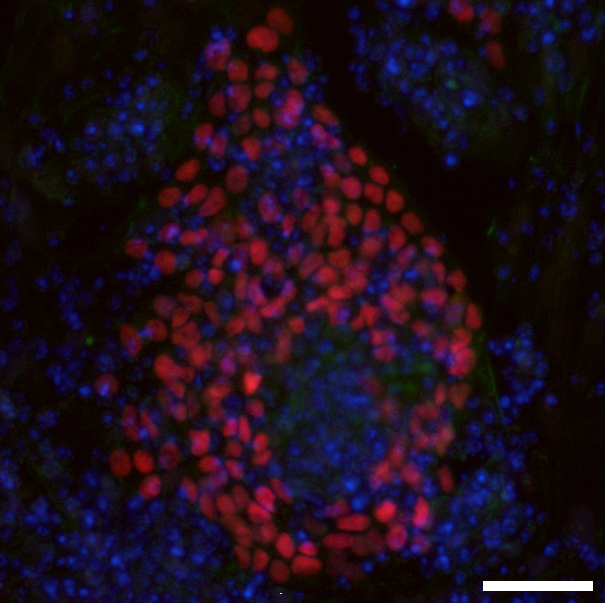

Supplement: Supplementary file 9 — Source data Fig. 6 [file 44318_2024_299_MOESM9_ESM.zip › Figure 6/6C/Dox_plus_DMSO_PRPH_g_MYCN_r_merge.tif]

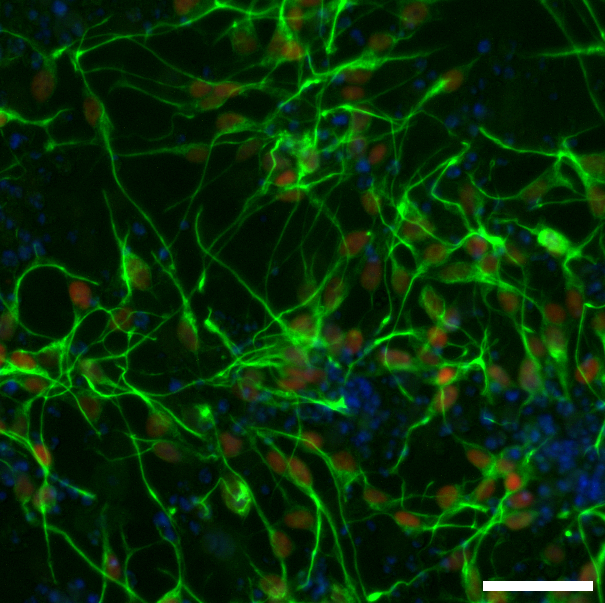

Supplement: Supplementary file 9 — Source data Fig. 6 [file 44318_2024_299_MOESM9_ESM.zip › Figure 6/6C/Dox_plus_STM_PRPH_g_MYCN_r_merge.tif]

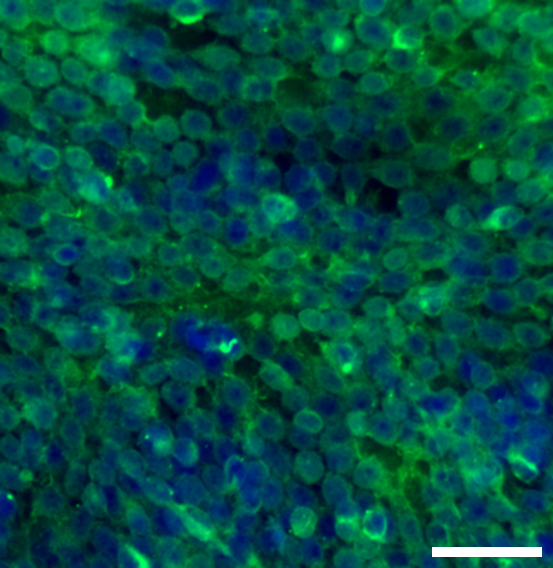

Supplement: Supplementary file 9 — Source data Fig. 6 [file 44318_2024_299_MOESM9_ESM.zip › Figure 6/6D/DMSO_Beta tub_merge.tif]

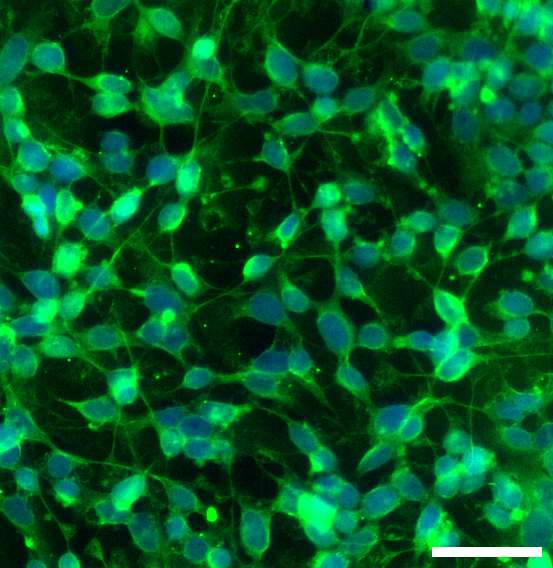

Supplement: Supplementary file 9 — Source data Fig. 6 [file 44318_2024_299_MOESM9_ESM.zip › Figure 6/6D/STM_Beta tub_merge.tif]

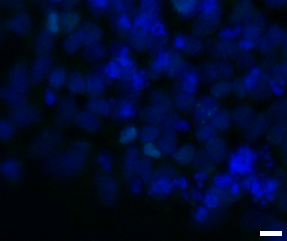

Supplement: Supplementary file 9 — Source data Fig. 6 [file 44318_2024_299_MOESM9_ESM.zip › Figure 6/6E/Dox_plus_DMSO_H2AX_g.tif]

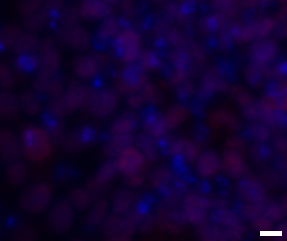

Supplement: Supplementary file 9 — Source data Fig. 6 [file 44318_2024_299_MOESM9_ESM.zip › Figure 6/6E/Dox_plus_DMSO_RPA32_r.tif]

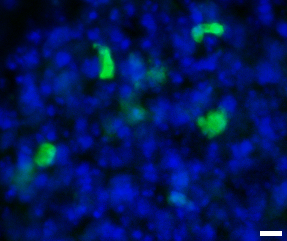

Supplement: Supplementary file 9 — Source data Fig. 6 [file 44318_2024_299_MOESM9_ESM.zip › Figure 6/6E/Dox_plus_STM_H2AX_g.tif]

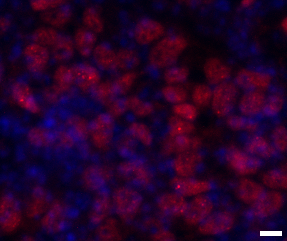

Supplement: Supplementary file 9 — Source data Fig. 6 [file 44318_2024_299_MOESM9_ESM.zip › Figure 6/6E/Dox_plus_STM_RPA32_r.tif]

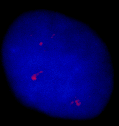

Supplement: Supplementary file 9 — Source data Fig. 6 [file 44318_2024_299_MOESM9_ESM.zip › Figure 6/6F/TetOshCtrl_dox+_RPA32_r.tif]

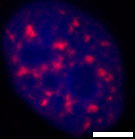

Supplement: Supplementary file 9 — Source data Fig. 6 [file 44318_2024_299_MOESM9_ESM.zip › Figure 6/6F/TetOshM3-1_dox+_RPA32_r.tif]

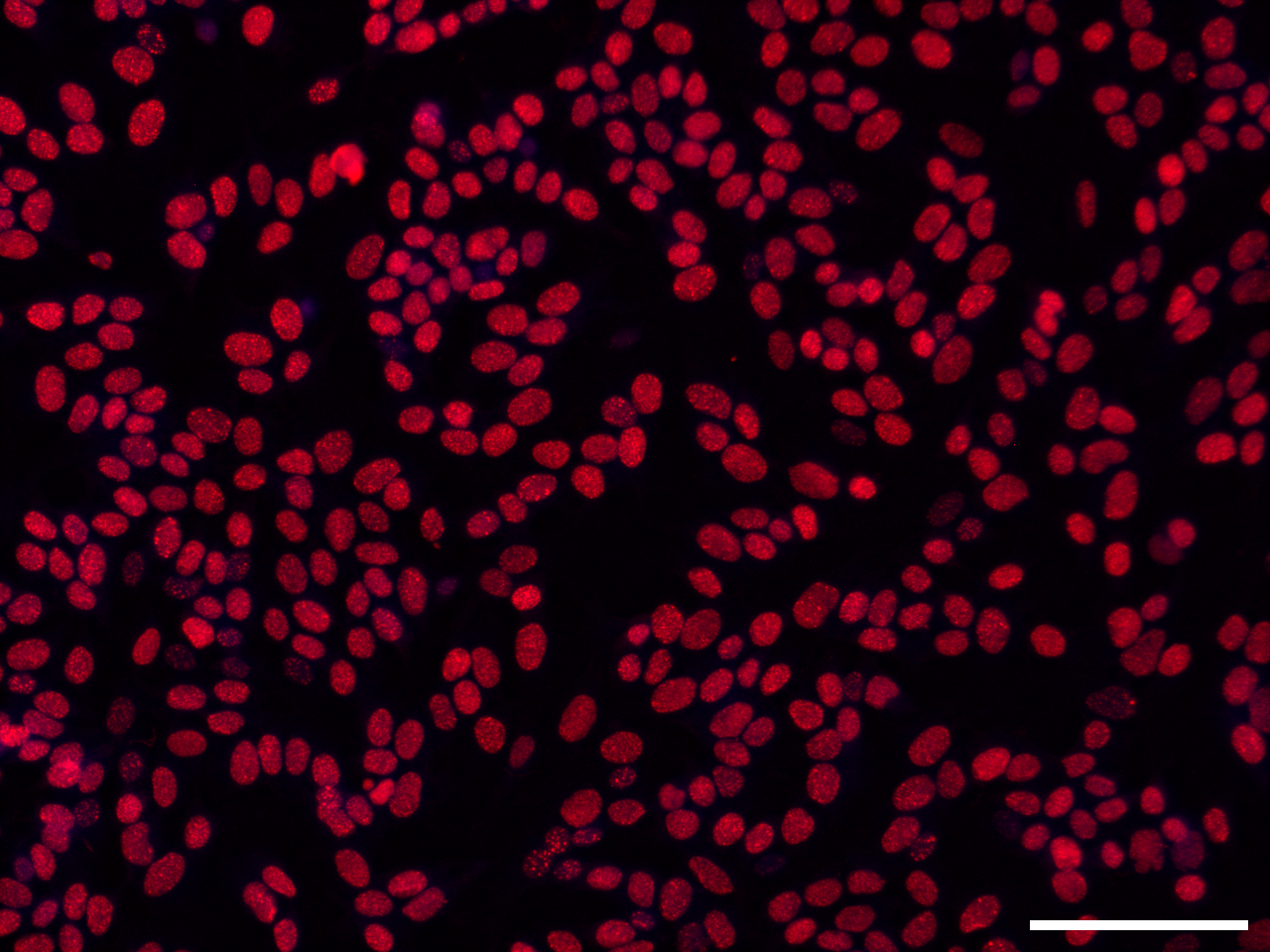

Supplement: Supplementary file 9 — Source data Fig. 6 [file 44318_2024_299_MOESM9_ESM.zip › Figure 6/6G/Combo_Doxorubicin_STM2457_24h_RPA32_r.tif]

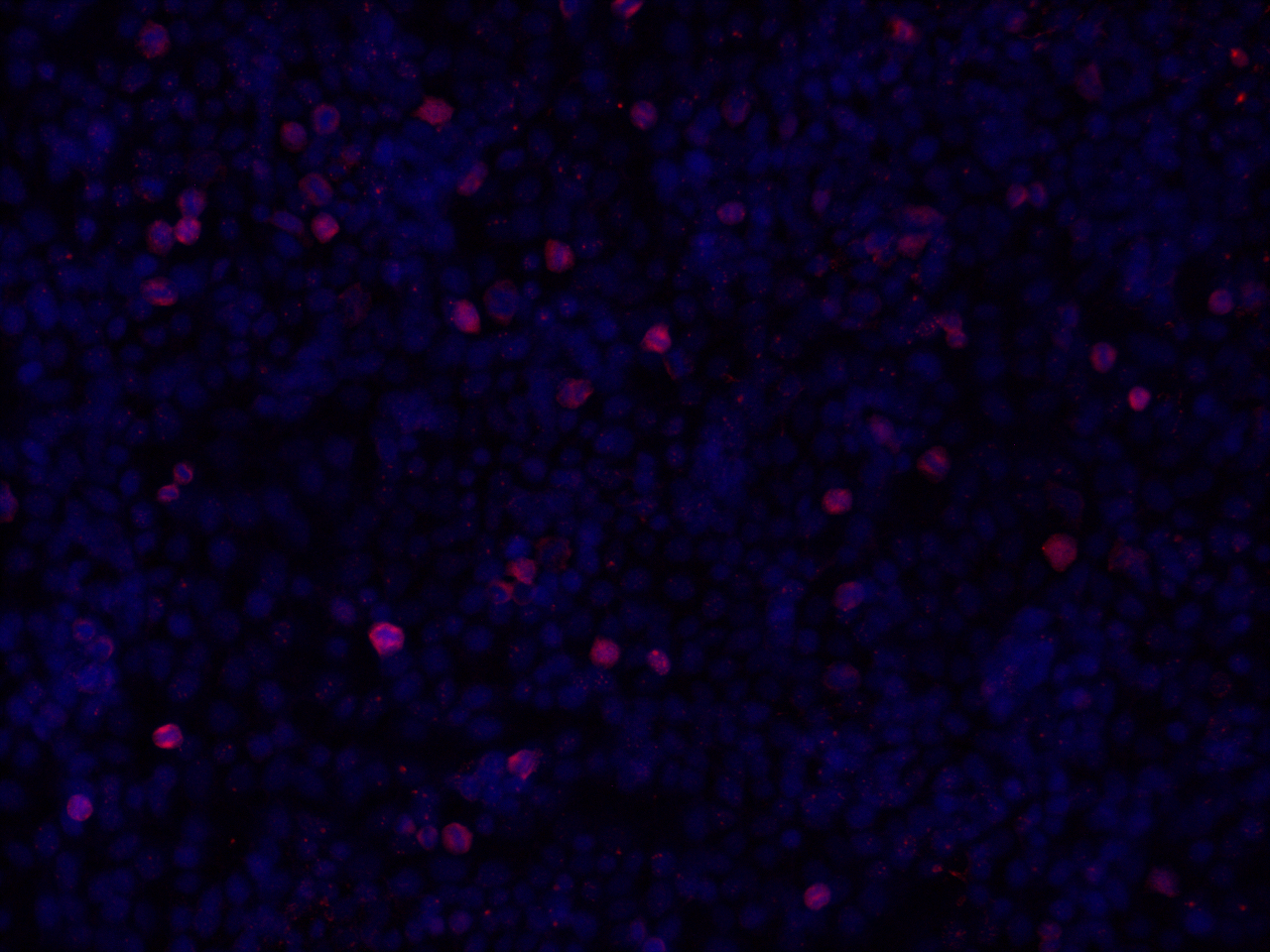

Supplement: Supplementary file 9 — Source data Fig. 6 [file 44318_2024_299_MOESM9_ESM.zip › Figure 6/6G/DMSO_24h_RPA32_r.tif]

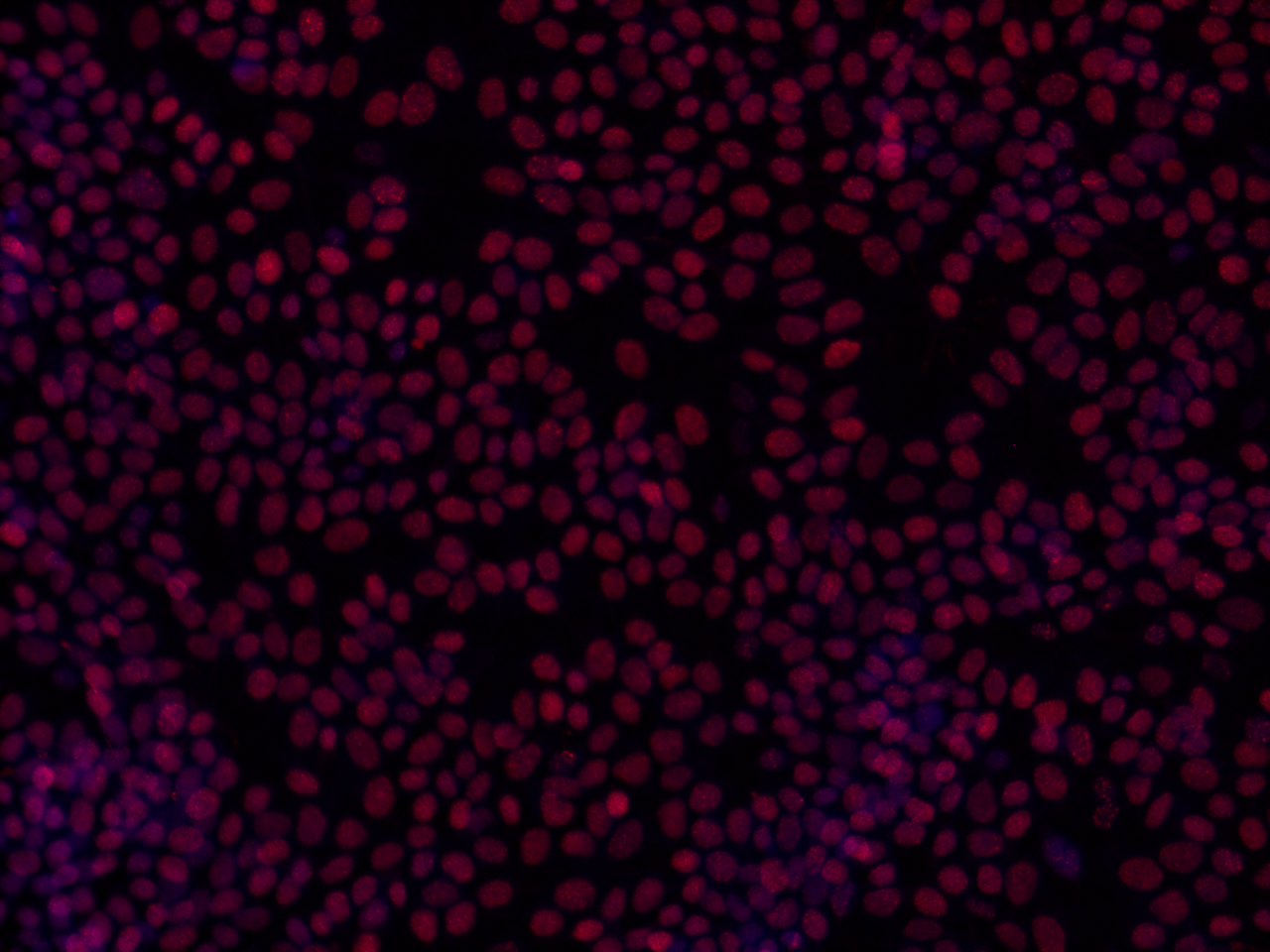

Supplement: Supplementary file 9 — Source data Fig. 6 [file 44318_2024_299_MOESM9_ESM.zip › Figure 6/6G/Doxorubicin_24h_RPA32_r.tif]

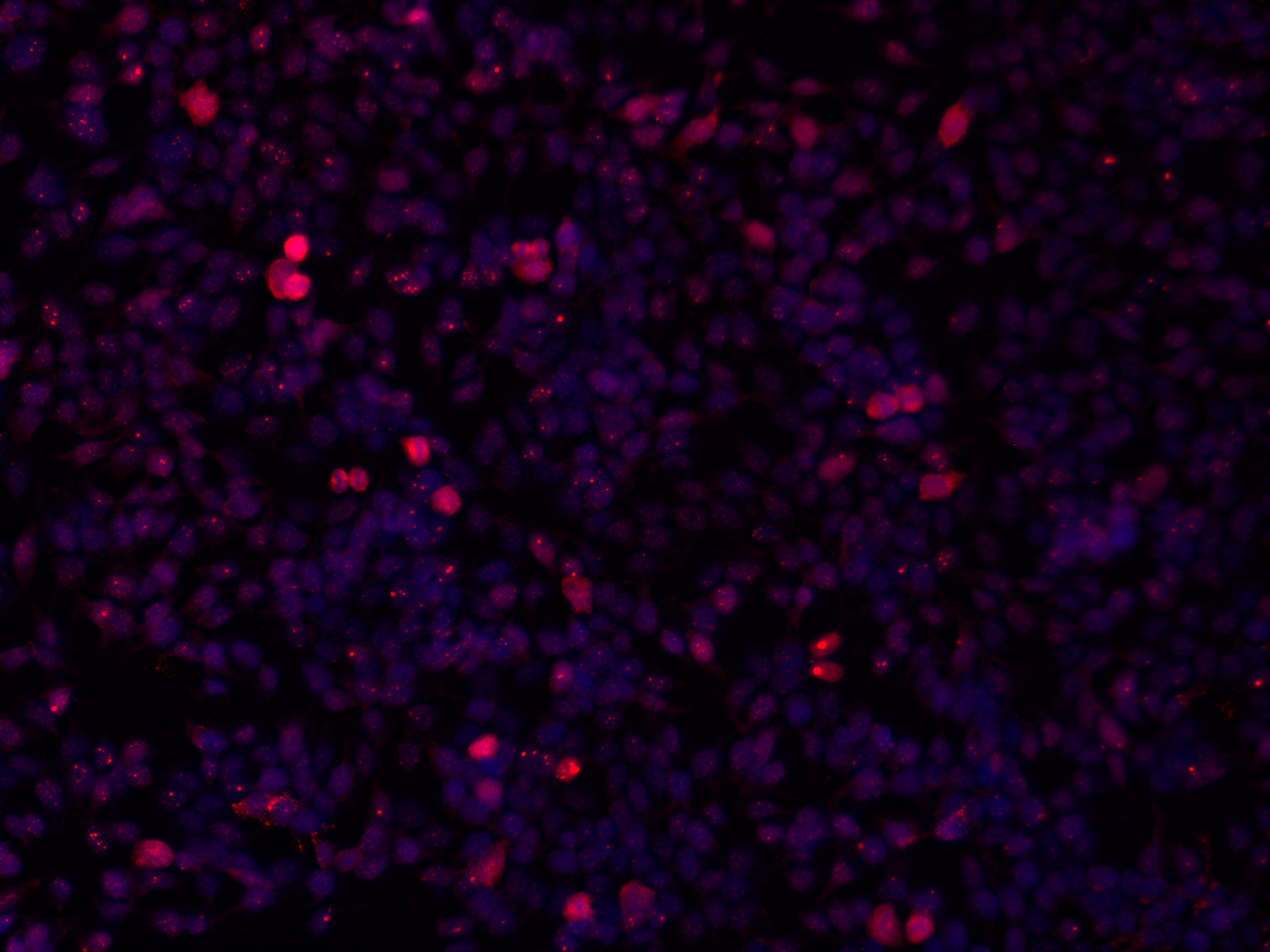

Supplement: Supplementary file 9 — Source data Fig. 6 [file 44318_2024_299_MOESM9_ESM.zip › Figure 6/6G/STM2457_24h_RPA32_r.tif]
